# Synthesis of Benzo[*b*]azocin‐2‐ones by Aryl Amination and Ring‐Expansion of α‐(Iodophenyl)‐β‐oxoesters

**DOI:** 10.1002/chem.201903139

**Published:** 2019-10-28

**Authors:** Anna Dierks, Jan Tönjes, Marc Schmidtmann, Jens Christoffers

**Affiliations:** ^1^ Institut für Chemie Carl von Ossietzky Universität Oldenburg 26111 Oldenburg Germany

**Keywords:** aryl amination, lactams, medium sized rings, ring expansion, scaffolds

## Abstract

Transformation of β‐oxoesters with PhI(OCOCF_3_)_2_ leads to α‐(*ortho*‐iodophenyl)‐β‐oxoesters. These materials are the starting point for the synthesis of 6‐carboxybenzo[*b*]azocin‐2‐ones by a sequence of aryl amination and ring transformation. This reaction sequence starts with copper‐catalyzed formation of *N*‐alkyl anilines from the iodoarenes and primary amines in the presence of K_3_PO_4_ as stoichiometric base. The intermediate products underwent ring transformation by addition of the nitrogen into the carbonyl group of the cycloalkanone, furnishing benzo‐annulated eight‐membered ring lactams. Under the same reaction conditions, the cyclohexanone and cycloheptanone derivatives gave no aminated products, but ring‐transformed to benzofuran derivatives. The title compounds of this investigation contain two points for further diversification (the lactam nitrogen and the carboxylate function), thus, the suitability of this compound class as a scaffold was proven by appropriate functionalizations. The first series of derivatizations of the scaffold was initiated by hydrogenolytic debenzylation of N‐benzyl derivative to provide the NH‐congener, which could be deprotonated with LDA and alkylated at nitrogen to give further examples of this compound class. Secondly, the ester function was submitted to saponification and the resulting carboxylic acid could be amidated using HATU as coupling reagent to furnish different amides.

## Graphical abstract

**Expanding rings**: A sequence of aryl amination of α‐(iodophenyl)‐β‐oxoesters followed by ring transformation is the key to accessing hexahydrobenzo[*b*]azocin‐2‐on‐6‐carboxylic esters.

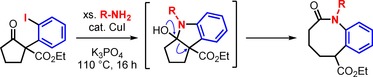

## Introduction

**Figure 1:**
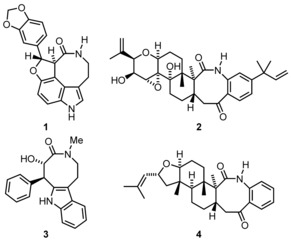
Four naturally occurring benzazocinone derivatives.

Organic compounds that contain a benzannulated eight‐membered lactam ring (i.e. benzazocin‐2‐ones)^1^ have been considered as potential drugs, for example as inhibitors of the angiotensin converting enzyme (ACE).^2^ Furthermore, they show affinity as ligands for the dopamine D_3_ 3 or the GABA_A_ receptor.^4^ Moreover, some natural products possess this structural motif (Figure 1): Decursivine (**1**), an antimalarial indole alkaloid from *Rhaphidophora decursiva*,^5^ sulpinine C (**2**), an antiinsectan metabolite from *Aspergillus sulphureus*,^6^ the tryptamine derived balasubramide (**3**) from *Clausena indica* 7 and asporyzin A (**4**) from the fungus *Aspergillus oryzae* associated with the red alga *Heterosiphonia japonica*.^8^ Benzazocinones possess two chiral boat‐like conformations and the phenylene ring in them defines an element of planar chirality; the inversion barrier has been studied by NMR investigations and DFT calculations to be in the range of 30–100 kJ mol^−1^.^9^, ^10^

Synthetic routes to the azocane ring system were recently reviewed by Voskressensky.^11^, ^12^ An obvious synthetic access to hexahydrobenzo[*b*]azocin‐2‐one derivatives is provided by Beckmann rearrangement of oximes from benzosuberones.^13^ A less evident, though very efficient access to the target structure is achieved by oxidative cleavage of cyclopenta[*b*]indole derivatives with periodate.^14^ Not necessarily most effective, but very interesting routes to eight‐membered ring lactams involve ring expanding transformations.^15^ For example, Tan et al.^16^ accessed the target structure by ring expansion of indanones in a reaction sequence, which started with an aldol reaction with the ester enolate of ethyl acetate followed by Weinreb‐amide formation. The ring expanding transformation was then initiated by oxidation of the aromatic ring with PIFA, which led to intramolecular *ipso*‐substitution with *trans*‐annular C−C‐bond cleavage. Very similar was the route published by Liu et al.,^17^ who have replaced the PIFA‐oxidation step by photocatalysis with a Ru complex. A formal [6+2] cyclization of silyloxy alkynes and vinylazetidines leading to monocyclic azocanones was very recently reported by Wu et al.^18^

**Scheme 1:**
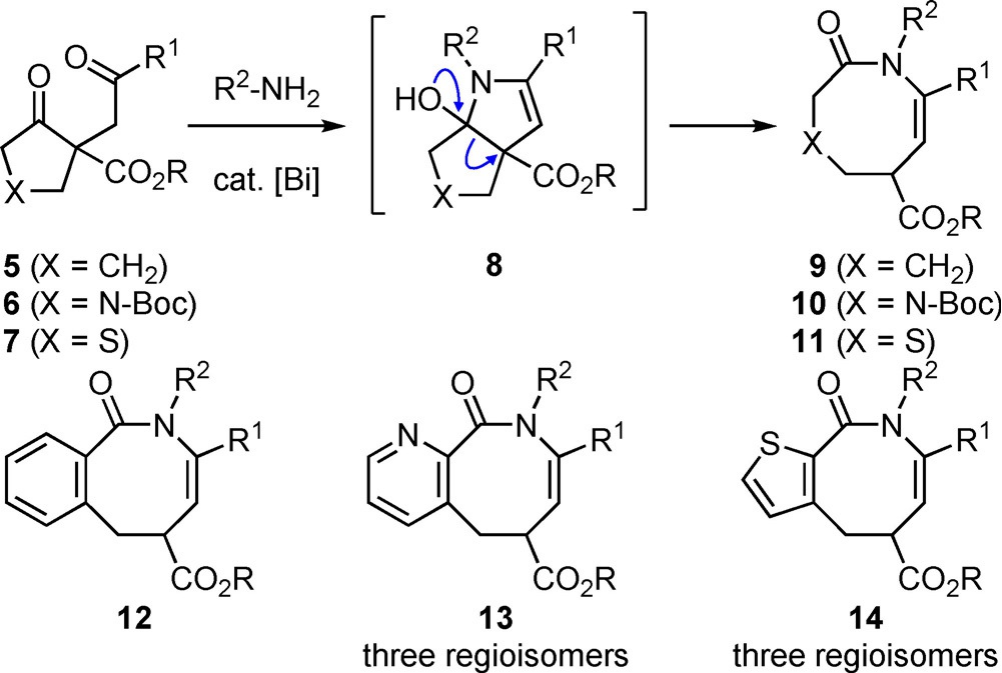
Preparation of eight‐membered ring lactams **9**–**14** by Bi‐catalyzed ring transformation of 1,4‐diketones **5**–**7** with primary amines; [Bi]=Bi(NO_3_)_3_⋅5 H_2_O.

We have reported an access to eight‐membered ring lactams by ring transformation of ten different β‐oxoesters **5** with 1,4‐dicarbonyl motif (Scheme 1). Bi‐catalyzed conversion with 25 primary amines R^2^‐NH_2_ via azabicyclo[3.3.0]‐intermediates **8** furnished a library of about 250 hexahydroazocinones **9**.^19^ This transformation was then applied to pyrrolidine **6** and tetrahydrothiophene derivatives **7** to furnish diazocanes **10** 20 and thiazocanes **11**.^21^ Furthermore, benzo‐ (products **12**),^10^ pyrido‐ (products **13** and two regioisomers)^22^ and thienoannulated congeners **14** (and two regioisomers)^23^ were prepared.

**Scheme 2:**
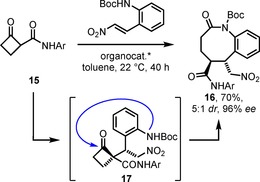
Ring transformation after organocatalyzed Michael addition; Ar=4‐C_6_H_4_CO_2_Et.

A very elegant, asymmetric organocatalytic approach to benzo[*b*]azocinones **16** was recently published by Rodrigues, Coquerel and co‐workers, who ring‐expanded cyclobutanone derivatives.^24^ An illustrative example is given in Scheme 2. Cyclobutanoncarboxamide **15** was converted in an organocatalyzed Michael addition with *ortho*‐(Boc‐amino)‐ω‐nitrostyrene to furnish the lactam **16** with good yield and remarkable stereoselectivity. The transformation proceeded via the product of the conjugated addition, compound **17**, which underwent ring transformation via an azabicyclo[4.2.0]intermediate after addition of the carbamate nitrogen to the carbonyl group within the four‐membered ring.

**Scheme 3:**
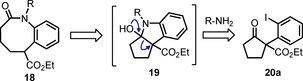
Preparation of hexahydrobenzo[*b*]azocin‐2‐on‐6‐carboxylates **18** from β‐oxoesters **20** with an α‐(*ortho*‐iodophenyl)‐residue.

In the present work we propose the preparation of hexahydrobenzo[*b*]azocin‐2‐on‐6‐carboxylates **18** by ring transformation of β‐oxoesters **20** with an α‐(*ortho*‐iodophenyl)‐residue (Scheme 3). Our plan is to perform aryl amination with primary amines R‐NH_2_. Expected products would undergo cyclization to azabicyclo[3.3.0]‐intermedates **19** similar to intermediates **8** in Scheme 1. The project is actually based on the availability of compounds **20**, which can be conveniently accessed by iodophenylation of a β‐oxoester with PhI(OCOCF_3_)_2_ [PIFA, phenyliodobis(trifluoroacetate)], which was recently reported by Shafir and co‐workers.^25^

## Results and Discussion

**Scheme 4:**
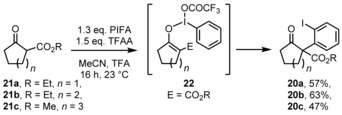
Literature known preparation of starting materials **20 a**–**20 c** from oxoesters **20 a**–**20 c** and PIFA.

The starting materials of this study, α‐(*ortho*‐iodophenyl)‐β‐oxoesters **20 a**–**20 c** were accessed from the β‐oxoesters **21 a**–**21 c** following the original report^25^ with stoichiometric amount of PIFA and TFAA (trifluoroacetic anhydride) in a mixture of MeCN and TFA (trifluoroacetic acid). In our hands, the yields in the range of 47–63 % were a little bit better compared to the literature (Scheme 4). The product constitution is proposed to result from a [3,3]‐sigmatropic rearrangement (“ioda‐Claisen reaction”) of an intermediate **22** which was formed by substitution of a trifluoroacetate residue by the enol tautomer of the oxoesters **21 a**–**21 c** at the hypervalent iodine atom. This rearrangement is followed by rearomatization by tautomerization and reductive elimination of TFA from a hypervalent iodine species.

**Scheme 5:**
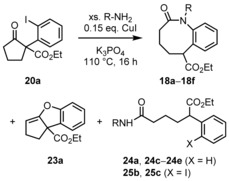
Benzazocinone formation after Ullmann‐type aryl amination, for residues R and X as well as yields see Table 1.

We have chosen the Buchwald–Hartwig^26^ coupling reaction^27^ for our first efforts for aryl amination of compound **20 a** with the primary amine BnNH_2_. As precatalysts we have chosen Pd_2_(dba)_3_ with BINAP, DPPF, and Xantphos as ligands and Cs_2_CO_3_, KHMDS and NaO*t*Bu as bases in refluxing toluene, however, acyclic product **24 a** was observed as the only isolable and unique compound together with several unspecified decomposition products. Compound **24 a** results from two processes: Pd‐catalyzed reductive deiodination and retro‐Claisen reaction induced by intermolecular nucleophilic attack of the amine to the endocyclic carbonyl group. Therefore, we turned to Ullmann‐type^28^ condensations^29^ with catalytic amounts of CuI and Cs_2_CO_3_ (with or without phenanthroline as ligand) in solvents like 1,4‐dioxane, DMF, and acetonitrile, and we were indeed able to detect the target structure **18 a** in the reaction mixture. Finally, inspired by reports of Buchwald et al.,^30^ we used K_3_PO_4_ as base, and the amount of product **18 a** increased (Scheme 5). After screening of reaction temperature and solvent, we ultimately identified the following optimal reaction conditions for the formation of compound **18 a**: 0.15 equivalents CuI and 2 equivalents K_3_PO_4_ in neat BnNH_2_ at 110 °C for 16 h gave 56 % yield of product **18 a**. Cyclopenta[*b*]benzofurane derivative **23 a** was formed as a byproduct and could be isolated in 9 % yield, which results presumably from Cu‐mediated carbon–oxygen coupling and subsequent elimination of water from an intermediate hemiacetal. Furthermore, deiodinated and ring‐opened byproduct **24 a** was isolated in 16 %. We then submitted various primary amines to the conversion with oxoester **18 a** under the optimized conditions and were able to isolate further five lactams **18 b**–**18 f** together with varying amounts of benzofuran **23 a** as well as acyclic products **24** and **25** with (X=H) or without (X=I) reductive deiodination as byproducts (see Table 1). For alkylamines (R=*n*Bu, *n*Hex, Cy and allyl) the products **18 b**–**18 e** were obtained in ca. 50 % yield. For 2‐ethoxyethylamine, the yield was slightly lower (product **18 f** in 38 % yield). Table 1 lists the yields of the major products **18 a**–**18 f** as well as the yields of byproducts **23 a**, **24 a**, **24 c**–**24 e**, **25 b**, and **25 c**.

**Table 1:** Residues R, X and yields of benzoazocinones **18** and byproducts **23 a**, **24** and **25**.

R	Product **18**	Byproduct **23 a**	Byproduct **24**	Byproduct **25**
Bn	56 % (**18 a**)	9 %	16 % (**24 a**)	0 %
*n*Bu	51 % (**18 b**)	0 %	0 %	15 % (**25 b**)
*n*Hex	50 % (**18 c**)	4 %	16 % (**24 c**)	2 % (**25 c**)
Cy	50 % (**18 d**)	11 %	13 % (**24 d**)	0 %
allyl	49 % (**18 e**)	0 %	20 % (**24 e**)	0 %
CH_2_CH_2_OEt	38 % (**18 f**)	0 %	0 %	0 %

**Scheme 6:**
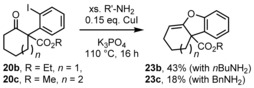
Formation of dibenzofurane and cyclohepta[*b*]benzofuran derivatives **23 b** and **23 c**.

Conversion of the congeners **20 b** and **20 c** under the respective reaction conditions with benzyl‐ or butylamine did not give lactams as products, but the dibenzofuran and cyclohepta[*b*]benzofuran derivatives **23 b** and **23 c** were isolated (in 43 and 18 % yield, respectively; Scheme 6).

**Figure 2:**
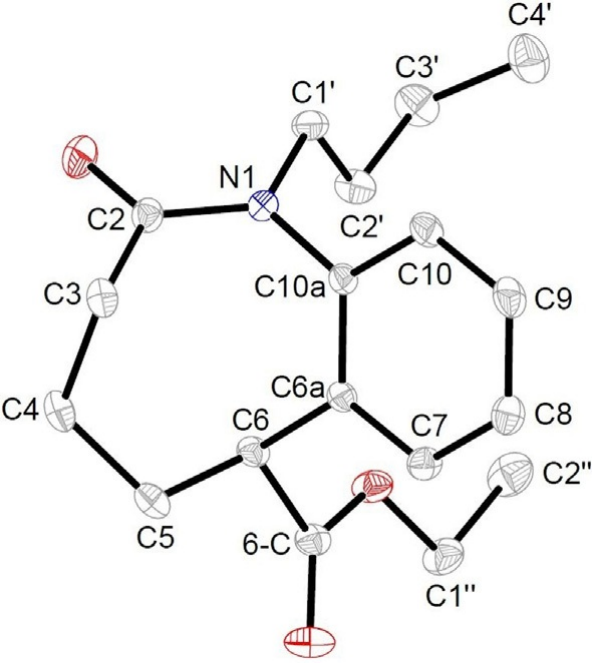
The ORTEP‐representation of the molecular structure of compound **18 b** in the solid state proves the constitution.

Compound **18 b** was obtained as a crystalline material suitable for single crystal X‐ray structure determination.^31^ In Figure 2, a representation of the molecular structure is given. Being a carboxamide, the nitrogen atom N1 is planar (angles C2‐N1‐C10a 123.26°, C2‐N1‐C1′ 120.30°, C1′‐N1‐C10a 116.12°, sum 359.68°) and the C2−N1 bond with a length of 1.3658 Å is rather a double bond. The bond N1−C10a with 1.4289 Å is a single bond. The eight and six membered rings are almost perpendicular at their junction (dihedral angles C2‐N1‐C10a‐C6a 60.75° and C4‐C5‐C6‐C6a 86.16°). Therefore, there seems to be no electronic influence of the amide group towards the aromatic ring, which is also reflected by the chemical shifts of the four aromatic protons in the ^1^H NMR spectrum (*δ* 7.21–7.35 ppm).

**Scheme 7:**
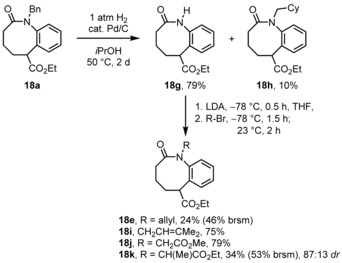
Hydrogenolytic debenzylation of the lactam‐nitrogen followed by alkylation reaction.

In order to prove the versatility of the benzoazocinones **18** as new heterocyclic scaffolds, we envisioned diversifying transformations at the lactam‐nitrogen and the exocyclic carboxyl function. First of all, the benzyl group of compound **18 a** was removed with H_2_/Pd/C to furnish compound **18 g** (79 %, Scheme 7). In order to achieve full conversion, the temperature had to be raised to 50 °C, upon which the aromatic ring of part of the starting material was hydrogenated to furnish the *N*‐(cyclohexylmethyl) congener **18 h** (10 %). After NH deprotonation with LDA, it was reacted with various alkyl bromides. First of all, the *N*‐allyl compound **18 e** was isolated in surprisingly low yield (24 %, 46 % brsm) together with some starting material **18 g**. On the other hand, the prenylation proceeded straightforwardly without allylic inversion (75 % of product **18 i**). With methyl bromoacetate, compound **18 j** was obtained in 79 % yield. Introducing some steric hindrance with the secondary halide ethyl α‐bromopropionate gave again lower yield (product **18 k** in 34 %, 53 % brsm) together with recovered starting materials **18 g**. Interestingly, this compound was isolated as two diastereoisomers with 87:13 *dr*, which is rather a remarkable selectivity considering the 1,5‐distance of the two stereocenters.

**Scheme 8:**
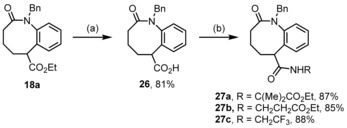
Ester saponification and amide coupling. Reagents and conditions: (a) NaOH, H_2_O‐EtOH, 80 °C, 16 h; (b) HATU, DIPEA, 1.5 equiv R‐NH_2_, CH_2_Cl_2_, 23 °C, 16 h.

For the second diversifying strategy we first submitted compound **18 a** to ester saponification yielding compound **26** in 81 % yield (Scheme 8). It was then coupled with the HATU–DIPEA protocol^32^ [HATU=*O*‐(7‐azabenzotriazol‐1‐yl)‐*N*,*N*,*N′*,*N′*‐tetramethyluronium hexafluorophosphate), DIPEA=ethyldiisopropylamine] with the ethyl esters of aminoisobutyric acid and β‐alanine to give the amides **27 a** and **27 b** in good yield (87 % and 85 %, respectively). By application of the same reaction conditions, trifluoroethylamine could be coupled to furnish compound **27 c** with 88 % yield.

**Scheme 9:**
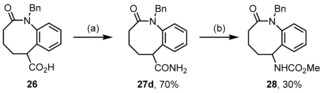
Preparation and Hofmann degradation of amide **27 d**. Reagents and conditions: (a) 1. 1.5 equiv Boc_2_O, 1.8 equiv pyridine, 1,4‐dioxane, 23 °C, 0.5 h; 2. 2.8 equiv (NH_4_)_2_CO_3_, 23 °C, 16 h; (b) 1.0 equiv PIDA, 2.5 equiv KOH, MeOH, CH_2_Cl_2_, 0 °C→23 °C, 16 h.

Furthermore, we intended to prepare the 6‐amino derivative of the scaffold by Hofmann degradation of the carboxylate function in compound **26**. We relied on a literature procedure applying the hypervalent iodine reagent PIDA [PhI(OAc)_2_] (Scheme 9).^20^ First of all, the parent unsubstituted amide **27 d** was prepared in 70 % yield by activation of the acid **26** with Boc_2_O and conversion of the mixed anhydride with hartshorn salt (ammonium carbonate). The degradation proceeded with PIDA and the intermediate isocyanate was removed with MeOH to furnish the carbamate **28**, however, the yield was moderate.

**Scheme 10:**
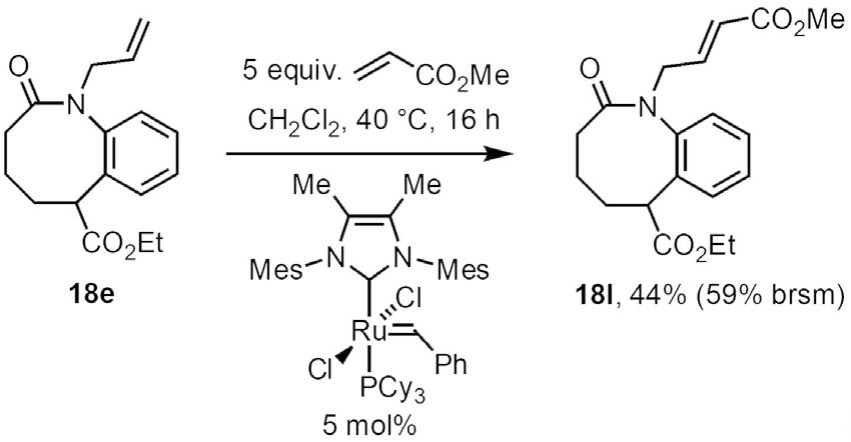
Olefin cross‐metathesis of allylic amide **18 e**; Mes=2,4,6‐Me_3_C_6_H_2_.

Finally, the *N*‐allyl group of compound **18 e** seemed to be perfectly suited for further transformations, for example, olefin cross‐metathesis. Therefore, we converted it with an excess of methyl acrylate in the presence of one of Evonik's catMETium RF catalysts^33^ (Scheme 10). The internal olefin **18 l** was obtained exclusively as *trans*‐diastereoisomer together with some unreacted starting material (44 % yield, 59 % brsm).

## Conclusions

A novel synthesis of benzo[*b*]azocin‐2‐ones by a sequence of aryl amination and ring transformation of ethyl 1‐(*ortho*‐iodophenyl)‐2‐oxocyclopentancarboxylate **20 a** was introduced. Additionally, the nitrogen atom and the carboxylate function define two points for further diversification, thus, the suitability of this compound class as a scaffold was proven by appropriate functionalization. Starting point of this investigation was the preparation of α‐(*ortho*‐iodophenyl)‐β‐oxoesters **20 a**–**20 c** by transformation of β‐oxoesters **21 a**–**21 c** with PhI(OCOCF_3_)_2_ (PIFA). After aryl amination of the cyclopentanone congener **20 a** with six primary amines, which was accomplished with catalytic amounts of CuI and K_3_PO_4_ as stoichiometric base, the intermediate *N*‐alkyl anilines underwent ring transformation by addition of the nitrogen into the carbonyl group of the cycloalkanone, furnishing the benzo‐annulated eight‐membered ring lactams **18 a**–**18 f** (38–56 % yield). Under the same reaction conditions, the cyclohexanone and cycloheptanone derivatives **20 b** and **20 c** gave no aminated products, but ring‐transformed to benzofuran derivatives **23 b** and **23 c**. The first series of derivatizations of the scaffold was initiated by hydrogenolytic debenzylation of *N*‐benzyl derivative **18 a** to provide the NH‐congener **18 g**, which could be deprotonated with LDA and alkylated at nitrogen to give further examples **18 i**–**18 k** of this compound class. Another representative (product **18 l**) was obtained by olefin cross‐metathesis of *N*‐allyl lactam **18 e** with methyl acrylate. Secondly, the ester function of compound **18 a** was submitted to saponification (81 % yield) and the resulting carboxylic acid **26** could be amidated using HATU as coupling reagent to furnish three different amides **27 a**–**27 c** (85–88 % yield). The *N*‐unsubstituted parent amide **27 d** was obtained by amidation with (NH_4_)_2_CO_3_ and could be further transformed by Hofmann degradation using PhI(OAc)_2_ (PIDA) and MeOH to give carbamate **28** (30 % yield).

## Experimental Section

**General**: Preparative column chromatography was carried out using Merck SiO_2_ (35–70 μm, type 60A) with hexanes (mixture of isomers, bp. 64–71 °C), *tert*‐butyl methyl ether (MTBE), EtOAc, and MeOH as eluents. TLC was performed on aluminum plates coated with SiO_2_ F_254_. ^1^H, ^19^F and ^13^C NMR spectra were recorded on Bruker Avance DRX 500 and 300 instruments. Multiplicities of carbon signals were determined with DEPT experiments. MS and HRMS spectra of products were obtained with a Waters Q‐TOF Premier (ESI, pos. mode) or Thermo Scientific DFS (EI) spectrometers. IR spectra were recorded on a Bruker Tensor 27 spectrometer equipped with a diamond ATR unit. Compounds **20 a**–**20 c** were literature known and prepared accordingly.^25^ All other starting materials were commercially available.

**General procedure A (GPA) for the α‐arylation of β‐oxoesters 21 a**–**21 c**:^25^ Under exclusion of air and moisture (nitrogen atmosphere), TFAA (1.5 equiv) was added dropwise to a stirred solution of PIFA (1.3 equiv) and TFA (1.5 L mol^−1^ PIFA) in MeCN (1.5 L mol^−1^ PIFA) and the resulting mixture was stirred at ambient temperature for 15 min. Then β‐oxoester **21** (1.0 equiv) was added and the resulting mixture was further stirred at ambient temperature for 16 h. The solvent was removed in vacuum and the residue was purified by column chromatography to yield arylated β‐oxoesters **20 a**–**20 c**.

**Ethyl 1‐(2‐iodophenyl)‐2‐oxocyclopentane‐1‐carboxylate (20 a)**:^25^ According to GPA, TFAA (2.52 g, 12.0 mmol), PIFA (4.47 g, 10.4 mmol) and β‐oxoester **21 a** (1.25 g, 8.00 mmol) were converted in TFA (16 mL) and MeCN (16 mL) to furnish the title compound **20 a** (1.64 g, 4.58 mmol, 57 %) after chromatography (SiO_2_, hexanes/MTBE 3:1, *R*_f_=0.30) as a colorless solid. M.p. 74 °C. ^1^H NMR (300 MHz, CDCl_3_): *δ*=1.24 (t, *J=*7.1 Hz, 3 H), 1.63–1.80 (m, 1 H), 2.03–2.15 (m, 1 H), 2.44–2.57 (m, 3 H), 3.20 (ddd, *J=*13.5 Hz, *J=*9.7 Hz, *J=*7.0 Hz, 1 H), 4.14–4.30 (m, 2 H), 6.92–6.97 (m, 2 H), 7.28 (td, *J=*7.8 Hz, *J=*1.3 Hz, 1 H), 7.93 (dd, *J=*8.3 Hz, *J=*1.2 Hz, 1 H) ppm. All spectroscopic data are in accordance with the literature.^25^ C_14_H_15_IO_3_ (358.18 g mol^−1^).

For the preparation of compounds **20 b** and **20 c** see the Supporting Information.

**General procedure B (GPB) for the Ullmann type coupling of β‐oxoesters 20 a**–**20 c with amines**: Under exclusion of air and moisture (nitrogen atmosphere), a Schlenk tube was charged with α‐arylated β‐oxoester **20** (1.0 equiv), K_3_PO_4_ (2.0–3.0 equiv) and CuI (15 mol %), three times evacuated and flushed with nitrogen. The amine (1‐1.8 L mol^−1^) was then added and the tube was tightly closed. The resulting mixture was stirred at 110 °C for 16 h and subsequently cooled to ambient temperature. The mixture was diluted with MTBE (20 L mol^−1^), water (20 L mol^−1^) and sat. aqueous NH_4_Cl solution (2 L mol^−1^) and the layers were separated. The aqueous layer was extracted with MTBE (2×20 L mol^−1^). The combined organic layers were dried (MgSO_4_), filtered and the solvent was removed in vacuo. The crude product was purified by column chromatography to furnish benzazocinones **18** together with byproducts **23**, **24**, and **25**.

**Conversion of β‐oxoester 20 a with benzylamine**: According to GPB, β‐oxoester **20 a** (179 mg, 500 μmol), K_3_PO_4_ (212 mg, 1.00 mmol) and CuI (14 mg, 75 μmol) were converted with benzylamine (0.5 mL). The crude product was purified by column chromatography (SiO_2_, hexanes/MTBE 1:2) to yield the benzofuran **23 a** (10 mg, 43 μmol, 9 %, *R*_f_=0.65) as a pale yellow oil. Secondly, the benzazocinone **18 a** (95 mg, 0.28 mmol, 56 %, *R*_f_=0.30) was eluted as a pale yellow solid. M.p. 60–63 °C. As the third fraction, the acyclic amide **24 a** (28 mg, 82 μmol, 16 %, *R*_f_=0.12) was obtained as a pale yellow oil.

**Ethyl 1‐benzyl‐2‐oxo‐1,2,3,4,5,6‐hexahydrobenzo[*b*]azocine‐6‐carboxylate (18 a)**: ^1^H NMR (500 MHz, CDCl_3_): *δ*=1.10 (t, *J=*7.1 Hz, 3 H), 1.49 (dddd, *J=*14.1 Hz, *J=*12.7 Hz, *J=*11.2 Hz, *J=*5.6 Hz, 1 H), 1.76–1.85 (m, 1 H), 1.88–1.96 (m, 2 H), 2.30–2.34 (m, 1 H), 2.38 (dd, *J=*14.1 Hz, *J=*5.0 Hz, 1 H), 3.20 (dd, *J=*11.2 Hz, *J=*0.9 Hz, 1 H), 3.97–4.03 (m, 2 H), 4.68 (d, *J=*14.0 Hz, 1 H), 5.33 (d, *J=*14.0 Hz, 1 H), 7.17–7.19 (m, 1 H), 7.22–7.30 (m, 8 H) ppm. ^13^C{^1^H} NMR (125 MHz, CDCl_3_): *δ*=14.06 (CH_3_), 24.42 (CH_2_), 32.28 (CH_2_), 33.13 (CH_2_), 44.69 (CH), 52.69 (CH_2_), 60.59 (CH_2_), 125.95 (CH), 127.31 (CH), 127.40 (CH), 127.92 (CH), 128.42 (2 CH), 128.49 (CH), 129.26 (2 CH), 136.74 (C), 139.01 (C), 140.62 (C), 173.68 (C), 173.99 (C) ppm. IR (ATR): *ṽ*=2941 (w), 2928 (w), 1728 (vs), 1651 (vs), 1493 (m), 1453 (m), 1393 (m), 1296 (m), 1225 (m), 1185 (vs), 1148 (m), 1027 (m), 759 (m), 733 (m), 701 (s) cm^−1^. HR‐MS (EI, 70 eV): calcd 337.1672 (for C_21_H_23_NO_3_^+^), found 337.1665 [*M* ^+^]. C_21_H_23_NO_3_ (337.42 g mol^−1^).

**Ethyl 2,8 b**–**dihydro‐1*H*‐cyclopenta[*b*]benzofuran‐8 b**,**carboxylate (23 a)**: ^1^H NMR (500 MHz, CDCl_3_): *δ*=1.16 (t, *J=*7.1 Hz, 3 H), 2.27 (ddd, *J=*11.6 Hz, *J=*9.9 Hz, *J=*7.9 Hz, 1 H), 2.44 (ddd, *J=*14.9 Hz, *J=*7.9 Hz, *J=*4.0 Hz, 1 H), 2.78 (dddd, *J=*14.9 Hz, *J=*9.9 Hz, *J=*5.3 Hz, *J=*1.5 Hz, 1 H), 2.85 (dd, *J=*11.6 Hz, *J=*5.3 Hz, 1 H), 4.07–4.13 (m, 2 H), 5.24 (dd, *J=*4.0 Hz, *J=*1.5 Hz, 1 H), 6.97–7.01 (m, 2 H), 7.23 (td, *J=*7.9 Hz, *J=*1.5 Hz, 1 H), 7.33 (dd, *J=*7.4 Hz, *J=*1.1 Hz, 1 H) ppm. ^13^C{^1^H} NMR (125 MHz, CDCl_3_): *δ*=13.98 (CH_3_), 31.32 (CH_2_), 37.84 (CH_2_), 61.40 (CH_2_), 63.00 (C), 101.56 (CH), 110.74 (CH), 122.51 (CH), 124.81 (CH), 129.03 (C), 129.17 (CH), 161.67 (C), 162.25 (C), 171.76 (C) ppm. IR (ATR): *ṽ*=2958 (m), 2929 (m), 2859 (m), 1727 (vs), 1686 (m), 1607 (m), 1456 (s), 1239 (s), 1152 (s), 1101 (m), 1017 (m), 835 (m), 751 (s) cm^−1^. HR‐MS (ESI): calcd 237.1097 (for C_14_H_14_LiO_3_^+^), found 237.1105 [*M*+Li^+^]. C_14_H_14_O_3_ (230.26 g mol^−1^).

**Ethyl 6‐(benzylamino)‐6‐oxo‐2‐phenylhexanoate (24 a)**: ^1^H NMR (500 MHz, CDCl_3_): *δ*=1.18 (t, *J=*7.1 Hz, 3 H), 1.54–1.69 (m, 2 H), 1.75–1.82 (m, 1 H), 2.04–2.12 (m, 1 H), 2.15–2.25 (m, 2 H), 3.52 (t, *J=*7.6 Hz, 1 H), 4.02–4.16 (m, 2 H), 4.40 (d, *J=*5.8 Hz, 2 H), 5.80 (br s, 1 H), 7.22–7.33 (m, 10 H) ppm. ^13^C{^1^H} NMR (125 MHz, CDCl_3_): *δ*=14.08 (CH_3_), 23.63 (CH_2_), 33.01 (CH_2_), 36.25 (CH_2_), 43.55 (CH_2_), 51.52 (CH), 60.77 (CH_2_), 127.21 (CH), 127.46 (CH), 127.80 (4 CH), 128.59 (2 CH), 128.65 (2 CH), 138.25 (C), 138.89 (C), 172.16 (C), 173.82 (C) ppm. IR (ATR): *ṽ*=3294 (w), 2931 (w), 1731 (s), 1646 (s), 1546 (m), 1456 (m), 1174 (m), 1150 (s), 1030 (m), 734 (m), 699 (vs) cm^−1^. HR‐MS (EI, 70 eV): calcd 339.1829 (for C_21_H_25_NO_3_^+^), found 339.1817 [*M* ^+^]. C_21_H_25_NO_3_ (339.44 g mol^−1^).

For the conversion of β‐oxoester **20 a** with *n*‐butylamine (products **18 b**, **25 b**), *n*‐hexylamine (products **18 c**, **24 c**, **25 c**), cyclohexylamine (products **18 d**, **24 d**), allylamine (products **18 e**, **24 e**), and 2‐ethoxyethylamine (product **18 f**) see the Supporting Information.

**Ethyl 2,3,4,4 a**‐**tetrahydrodibenzofuran‐4 a**‐**carboxylate (23 b)**: According to GPB, β‐oxoester **20 b** (105 mg, 279 μmol), K_3_PO_4_ (178 mg, 837 μmol) and CuI (8 mg, 0.04 mmol) were converted in *n‐*butylamine (0.5 mL) to yield the title compound **23 b** (29 mg, 0.12 mmol, 43 %) after chromatography (SiO_2_, hexanes/MTBE 20:1, *R*_f_=0.23) as a colorless oil. ^1^H NMR (500 MHz, CDCl_3_): *δ*=1.16 (t, *J=*7.1 Hz, 3 H), 1.57–1.64 (m, 2 H), 1.86–1.90 (m, 1 H), 2.19–2.31 (m, 2 H), 2.83 (dd, *J=*8.6 Hz, *J=*3.1 Hz, 1 H), 4.11 (q, *J=*7.1 Hz, 2 H), 5.31 (t, *J=*3.8 Hz, 1 H), 6.91–6.96 (m, 2 H), 7.20 (td, *J=*7.9 Hz, *J=*1.2 Hz, 1 H), 7.31 (dd, *J=*7.4 Hz, *J=*0.8 Hz, 1 H) ppm. ^13^C{^1^H} NMR (125 MHz, CDCl_3_): *δ*=13.96 (CH_3_), 19.03 (CH_2_), 21.97 (CH_2_), 29.84 (CH_2_), 54.13 (C), 61.46 (CH_2_), 99.97 (CH), 109.80 (CH), 121.78 (CH), 124.05 (CH), 128.62 (C), 129.32 (CH), 155.61 (C), 157.87 (C), 171.66 (C) ppm. IR (ATR): *ṽ*=2981 (w), 2936 (w), 2916 (w), 1728 (vs), 1609 (w), 1596 (w), 1472 (m), 1461 (s), 1223 (vs), 1174 (m), 1157 (m), 1128 (m), 1102 (m), 1087 (vs), 1072 (m), 1022 (m), 751 (s) cm^−1^. HR‐MS (ESI): calcd 251.1254 (for C_15_H_16_LiO_3_^+^), found 251.1251 [*M*+Li^+^]. C_15_H_16_O_3_ (244.29 g mol^−1^).

**Methyl 8,9,10,10 a**‐**tetrahydro‐7*H*‐cyclohepta[*b*]benzofuran‐10 a**‐**carboxylate (23 c)**: According to GPB, β‐oxoester **20 c** (105 mg, 279 μmol), K_3_PO_4_ (178 mg, 837 μmol) and CuI (8 mg, 0.04 mmol) were converted in benzylamine (0.5 mL) to yield the title compound **23 c** (12 mg, 49 μmol, 18 %) after chromatography (SiO_2_, hexanes/MTBE 20:1, *R*_f_=0.21) as a colorless solid. M.p. 75 °C. ^1^H NMR (500 MHz, CDCl_3_): *δ*=1.38–1.47 (m, 1 H), 1.68–1.80 (m, 3 H), 2.04–2.10 (m, 1 H), 2.12–2.16 (m, 2 H), 2.59–2.63 (m, 1 H), 3.73 (s, 3 H), 5.66 (dd, *J=*7.5 Hz, *J=*6.5 Hz, 1 H), 6.85 (d, *J=*8.0 Hz, 1 H), 6.93 (td, *J=*7.5 Hz, *J=*0.9 Hz, 1 H), 7.20 (td, *J=*8.0 Hz, *J=*1.4 Hz, 1 H), 7.27 (dd, *J=*7.2 Hz, *J=*1.1 Hz, 1 H) ppm. ^13^C{^1^H} NMR (125 MHz, CDCl_3_): *δ*=24.55 (CH_2_), 27.52 (CH_2_), 28.85 (CH_2_), 35.45 (CH_2_), 52.78 (CH_3_), 58.75 (C), 104.59 (CH), 109.47 (CH), 121.60 (CH), 123.81 (CH), 129.46 (CH), 129.57 (C), 156.98 (C), 159.98 (C), 171.15 (C) ppm. IR (ATR): *ṽ*=2926 (m), 2851 (w), 1732 (vs), 1701 (m), 1597 (m), 1476 (s), 1463 (s), 1237 (vs), 1221 (vs), 1158 (m), 1137 (m), 1093 (m), 1073 (m), 1057 (m), 999 (m), 820 (m), 749 (vs) cm^−1^. HR‐MS (ESI): calcd 251.1254 (for C_15_H_16_LiO_3_^+^), found 251.1257 [*M*+Li^+^]. C_15_H_16_O_3_ (244.29 g mol^−1^).

***N*** **‐Debenzylation of benzazocinone 18 a**: A suspension of 10 % Pd/C (883 mg, 830 μmol) and benzazocinone **18 a** (560 mg, 1.66 mmol) in *i*PrOH (8 mL) was stirred at 50 °C for 2 d under an atmosphere of hydrogen (1 bar). The mixture was then filtered and the solvent was removed in vacuo. The mixture was submitted to column chromatography (SiO_2_, hexanes/MTBE 1:5) to yield in the first fraction benzazocinone **18 h** (59 mg, 0.17 mmol, 10 %, *R*_f_=0.40) as a colorless oil. Secondly, benzazocinone **18 g** (324 mg, 1.31 mmol, 79 %, *R*_f_=0.16) was obtained as a colorless solid. M.p. 95–100 °C.

**Ethyl 2‐oxo‐1,2,3,4,5,6‐hexahydrobenzo[*b*]azocine‐6‐carboxylate (18 g)**: ^1^H NMR (500 MHz, CDCl_3_): *δ*=1.18 (t, *J=*7.1 Hz, 3 H), 1.58–1.67 (m, 1 H), 1.73–1.82 (m, 1 H), 1.93–1.98 (m, 2 H), 2.29–2.33 (m, 1 H), 2.50–2.53 (m, 1 H), 3.71 (d, *J=*10.8 Hz, 1 H), 4.09–4.19 (m, 2 H), 7.16–7.18 (m, 1 H), 7.26–7.32 (m, 2 H), 7.35–7.36 (m, 1 H), 8.29 (s, 1 H) ppm. ^13^C{^1^H} NMR (125 MHz, CDCl_3_): *δ*=14.00 (CH_3_), 23.65 (CH_2_), 32.14 (CH_2_), 32.51 (CH_2_), 45.02 (CH), 60.95 (CH_2_), 125.68 (CH), 127.14 (CH), 127.84 (CH), 128.24 (CH), 135.52 (C), 137.55 (C), 173.92 (C), 176.67 (C) ppm. IR (ATR): *ṽ*=3189 (w), 2945 (w), 1727 (s), 1659 (vs), 1495 (m), 1443 (m), 1390 (m), 1371 (m), 1301 (m), 1222 (m), 1185 (s), 1142 (m), 1096 (m), 1048 (m), 1017 (m), 764 (s), 734 (m) cm^−1^. HR‐MS (EI, 70 eV): calcd 247.1203 (for C_14_H_17_NO_3_^+^), found 247.1196 [*M* ^+^]. C_14_H_17_NO_3_ (247.29 g mol^−1^).

**Ethyl 1‐(cyclohexylmethyl)‐2‐oxo‐1,2,3,4,5,6‐hexahydrobenzo[*b*]azocine‐6‐carboxylate (18 h)**: ^1^H NMR (500 MHz, CDCl_3_): *δ*=1.05–1.19 (m, 4 H), 1.17 (t, *J=*7.1 Hz, 3 H), 1.51–1.71 (m, 7 H), 1.80–1.86 (m, 3 H), 1.90–1.96 (m, 1 H), 2.23 (dd, *J=*11.1 Hz, *J=*8.1 Hz, 1 H), 2.43–2.46 (m, 1 H), 3.22 (dd, *J=*13.5 Hz, *J=*5.2 Hz, 1 H), 3.63 (d, *J=*10.7 Hz, 1 H), 4.06–4.21 (m, 3 H), 7.20–7.22 (m, 1 H), 7.27–7.31 (m, 2 H), 7.33–7.36 (m, 1 H) ppm. ^13^C{^1^H} NMR (125 MHz, CDCl_3_): *δ*=14.12 (CH_3_), 24.27 (CH_2_), 25.80 (CH_2_), 26.00 (CH_2_), 26.25 (CH_2_), 31.51 (CH_2_), 31.91 (CH_2_), 32.16 (CH_2_), 33.26 (CH_2_), 36.82 (CH), 44.81 (CH), 55.38 (CH_2_), 60.91 (CH_2_), 125.52 (CH), 126.99 (CH), 128.02 (CH), 128.20 (CH), 138.68 (C), 141.46 (C), 173.93 (C), 174.43 (C) ppm. IR (ATR): *ṽ*=2924 (m), 2851 (w), 1732 (vs), 1652 (vs), 1493 (m), 1450 (m), 1395 (m), 1299 (m), 1224 (m), 1183 (m), 1150 (m), 1097 (m), 1048 (m), 1025 (m), 764 (m), 735 (m) cm^−1^. HR‐MS (EI, 70 eV): calcd 343.2142 (for C_21_H_29_NO_3_^+^), found 343.2152 [*M* ^+^]. C_21_H_29_NO_3_ (343.47 g mol^−1^).

**General procedure C (GPC) for the** ***N*** **‐alkylation of benzazocinone 18 g**: Under exclusion of air and moisture (nitrogen atmosphere) and at −78 °C, *n*BuLi (2.5 mol L^−1^ in hexanes, 1.05 equiv) was added dropwise to a stirred solution of diisopropylamine (1.05 equiv) in abs. THF (3 L mol^−1^). After stirring this mixture for 15 min at −78 °C, a solution of benzazocinone **18 g** (1.00 equiv) in abs. THF (2 L mol^−1^) was added and the resulting mixture was further stirred at −78 °C for 30 min. The alkyl bromide (1.05 equiv) was then added and the resulting mixture was stirred at −78 °C for 1.5 h and for further 2 h at ambient temperature. Subsequently, the mixture was diluted with hydrochloric acid (1 mol L^−1^, 4 L mol^−1^) and extracted with MTBE (3×4 L mol^−1^). The combined organic layers were dried (MgSO_4_), filtered and the solvent was removed in vacuo. The crude product was purified by column chromatography to yield benzazocinones **18 e**, **18 i**–**18 k**.

**Ethyl 1‐allyl‐2‐oxo‐1,2,3,4,5,6‐hexahydrobenzo[*b*]azocine‐6‐carboxylate (18 e)**: According to GPC, benzazocinone **18 g** (124 mg, 500 μmol), *n*BuLi (0.21 mL, 2.5 mol L^−1^ in hexanes, 0.53 mmol) and *i*Pr_2_NH (54 mg, 0.53 mmol) were converted with allyl bromide (64 mg, 0.53 mmol) to yield in the first fraction the title compound **18 e** (34 mg, 0.12 mmol, 24 %, *R*_f_=0.39) after chromatography (SiO_2_, hexanes/MTBE 1:5) as a colorless oil. Secondly, starting material **18 g** (60 mg, 0.24 mmol, 48 %, *R*_f_=0.16) was recovered in another fraction.

**Ethyl 2‐oxo‐1‐prenyl‐1,2,3,4,5,6‐hexahydrobenzo[*b*]azocine‐6‐carboxylate (18 i)**: According to GPC, benzazocinone **18 g** (124 mg, 500 μmol), *n*BuLi (0.21 mL, 2.5 mol L^−1^ in hexanes, 0.53 mmol) and *i*Pr_2_NH (54 mg, 0.53 mmol) were converted with prenyl bromide (79 mg, 0.53 mmol) to yield the title compound **18 i** (119 mg, 377 μmol, 75 %) after chromatography (SiO_2_, hexanes/MTBE 1:5, *R*_f_=0.43) as a colorless oil. ^1^H NMR (500 MHz, CDCl_3_): *δ*=1.15 (t, *J=*7.1 Hz, 3 H), 1.46–1.55 (m, 1 H), 1.49 (s, 3 H), 1.61 (s, 3 H), 1.72–1.91 (m, 3 H), 2.21 (dd, *J=*11.1 Hz, *J=*7.9 Hz, 1 H), 2.40 (dd, *J=*13.5 Hz, *J=*4.7 Hz, 1 H), 3.51 (d, *J=*11.2 Hz, 1 H), 3.97 (dd, *J=*14.4 Hz, *J=*7.3 Hz, 1 H), 4.11 (q, *J=*7.1 Hz, 2 H), 4.85 (dd, *J=*14.4 Hz, *J=*7.3 Hz, 1 H), 5.24 (t, *J=*7.3 Hz, 1 H), 7.19–7.22 (m, 1 H), 7.24–7.27 (m, 2 H), 7.29–7.32 (m, 1 H) ppm. ^13^C{^1^H} NMR (125 MHz, CDCl_3_): *δ*=14.08 (CH_3_), 17.69 (CH_3_), 24.24 (CH_2_), 25.58 (CH_3_), 32.14 (CH_2_), 32.99 (CH_2_), 44.75 (CH), 46.51 (CH_2_), 60.76 (CH_2_), 118.14 (CH), 126.01 (CH), 126.80 (CH), 127.86 (CH), 128.35 (CH), 136.63 (C), 139.10 (C), 140.55 (C), 173.52 (C), 173.95 (C) ppm. IR (ATR): *ṽ*=2924 (w), 1733 (s), 1652 (s), 1495 (m), 1456 (m), 1445 (m), 1395 (m), 1297 (m), 1227 (m), 1184 (s), 1149 (m), 1099 (m), 1049 (m), 1027 (m), 769 (m), 738 (m) cm^−1^. HR‐MS (EI, 70 eV): calcd 315.1829 (for C_19_H_25_NO_3_^+^), found 315.1835 [*M* ^+^]. C_19_H_25_NO_3_ (315.41 g mol^−1^).

For the preparation of compounds **18 j** and **18 k** see the Supporting Information.

**Ethyl (*E*)‐1‐[3‐(methoxycarbonyl)‐2‐propenyl]‐2‐oxo‐1,2,3,4,5,6‐hexahydrobenzo[*b*]azocine‐6‐carboxylate (18 l)**: Methyl acrylate (215 mg, 2.50 mmol) and catMETium RF {Benzylidenedichloro[4,5‐dimethyl‐1,3‐bis(2,4,6‐trimethylphenyl)‐4‐imidazolin‐2‐ylidene](tricyclohexylphosphano)ruthenium(II)} (25 μmol, 22 mg) were added to a solution of benzazocinone **18 e** (144 mg, 501 μmol) in degassed CH_2_Cl_2_ (1.5 mL) and the resulting mixture was stirred at 40 °C for 16 h. All volatile materials were evaporated and the crude product was purified by column chromatography (SiO_2_, hexanes/EtOAc 1:1) to yield the title compound **18 l** (76 mg, 0.22 mmol, 44 %, *R*_f_=0.27) as a colorless oil. As a second fraction, the starting material **18 e** (38 mg, 0.13 mmol, 26 %, *R*_f_=0.35) was recovered. ^1^H NMR (500 MHz, CDCl_3_): *δ*=1.15 (t, *J=*7.1 Hz, 3 H), 1.55 (dddd, *J=*14.2 Hz, *J=*12.4 Hz, *J=*11.1 Hz, *J=*5.6 Hz, 1 H), 1.77–1.86 (m, 1 H), 1.88–1.97 (m, 2 H), 2.27–2.31 (m, 1 H), 2.46 (dd, *J=*14.2 Hz, *J=*4.9 Hz, 1 H), 3.46 (dd, *J=*11.1 Hz, *J=*0.8 Hz, 1 H), 3.69 (s, 3 H), 4.11 (q, *J=*7.1 Hz, 2 H), 4.32 (ddd, *J=*15.4 Hz, *J=*6.7 Hz, *J=*1.1 Hz, 1 H), 4.79 (ddd, *J=*15.4 Hz, *J=*6.4 Hz, *J=*1.4 Hz, 1 H), 5.92 (dt, *J=*15.7 Hz, *J=*1.3 Hz, 1 H), 6.97 (dt, *J=*15.7 Hz, *J=*6.5 Hz, 1 H), 7.20–7.22 (m, 1 H), 7.28–7.36 (m, 3 H) ppm. ^13^C{^1^H} NMR (125 MHz, CDCl_3_): *δ*=13.93 (CH_3_), 24.22 (CH_2_), 31.96 (CH_2_), 32.90 (CH_2_), 44.84 (CH), 49.98 (CH_2_), 51.49 (CH_3_), 60.98 (CH_2_), 123.88 (CH), 125.62 (CH), 127.20 (CH), 128.27 (CH), 128.92 (CH), 138.90 (C), 140.20 (C), 141.65 (CH), 166.13 (C), 173.60 (C), 174.10 (C) ppm. IR (ATR): *ṽ*=2949 (w), 1724 (vs), 1652 (vs), 1494 (m), 1454 (m), 1441 (m), 1390 (m), 1299 (m), 1276 (m), 1225 (m), 1185 (s), 1169 (s), 1150 (m), 1097 (m), 1045 (m), 1022 (m), 996 (m), 972 (m), 765 (m), 740 (m), 718 (m) cm^−1^. HR‐MS (EI, 70 eV): calcd 345.1571 (for C_19_H_23_NO_5_^+^), found 345.1566 [*M* ^+^]. C_19_H_23_NO_5_ (345.40 g mol^−1^).

**1‐Benzyl‐2‐oxo‐1,2,3,4,5,6‐hexahydrobenzo[*b*]azocine‐6‐carboxylic acid (26)**: An aqueous solution of NaOH (0.5 mol L^−1^, 40 mL) was added to a solution of benzazocinone **18 a** (700 mg, 2.07 mmol) in EtOH (2 mL) and the resulting mixture was stirred at 80 °C for 16 h. Subsequently, the mixture was acidified with hydrochloric acid (1 mol L^−1^, 25 mL) and extracted with CH_2_Cl_2_ (3×30 mL). The combined organic layers were dried (MgSO_4_), filtered and the solvent was removed in vacuo to yield the title compound **26** (519 mg, 1.68 mmol, 81 %) as a colorless solid. M.p. 166–170 °C. ^1^H NMR (500 MHz, CDCl_3_): *δ*=1.51 (dddd, *J=*14.1 Hz, *J=*12.6 Hz, *J=*11.0 Hz, *J=*5.5 Hz, 1 H), 1.77–1.86 (m, 1 H), 1.90–1.97 (m, 2 H), 2.40 (dd, *J=*12.0 Hz, *J=*8.2 Hz, 1 H), 2.45 (dd, *J=*14.1 Hz, *J=*4.8 Hz, 1 H), 3.29 (d, *J=*11.0 Hz, 1 H), 4.86 (d, *J=*14.1 Hz, 1 H), 5.17 (d, *J=*14.1 Hz, 1 H), 7.15 (dd, *J=*7.8 Hz, *J=*1.0 Hz, 1 H), 7.21–7.30 (m, 6 H), 7.33 (td, *J=*7.8 Hz, *J=*1.4 Hz, 1 H), 7.42 (dd, *J=*7.8 Hz, *J=*1.3 Hz, 1 H), 10.45 (br s, 1 H) ppm. ^13^C{^1^H} NMR (125 MHz, CDCl_3_): *δ*=24.18 (CH_2_), 31.90 (CH_2_), 32.78 (CH_2_), 44.59 (CH), 52.87 (CH_2_), 125.86 (CH), 127.46 (CH), 127.50 (CH), 128.02 (CH), 128.46 (2 CH), 128.74 (CH), 129.03 (2 CH), 136.27 (C), 138.57 (C), 140.36 (C), 174.68 (C), 177.26 (C) ppm. IR (ATR): *ṽ*=3044 (m), 2946 (m), 1728 (vs), 1625 (vs), 1598 (s), 1496 (m), 1456 (m), 1441 (m), 1411 (m), 1287 (m), 1224 (m), 1173 (s), 1145 (m), 781 (m), 761 (m), 722 (m), 701 (s), 681 (m), 640 (m) cm^−1^. HR‐MS (EI, 70 eV): calcd 309.1359 (for C_19_H_19_NO_3_^+^), found 309.1368 [*M* ^+^]. C_19_H_19_NO_3_ (309.37 g mol^−1^). The compound was reported in the literature before, but insufficiently characterized.14b

**General procedure D (GPD) for the amide coupling of benzazocinone 26**: HATU (1.1 equiv) and DIPEA (1.1–2.2 equiv) were added to a stirred solution of benzazocinone **26** (1.0 equiv) and the primary amine (1.5 equiv) in CH_2_Cl_2_ (5 L mol^−1^) and the resulting mixture was stirred at ambient temperature for 16 h. Subsequently, the mixture was washed with water (1×10 L mol^−1^), sat. aq. NaHCO_3_ solution (1×10 L mol^−1^) and brine (1×10 L mol^−1^). The organic layer was dried (MgSO_4_), filtered and the solvent was removed in vacuo. The crude product was purified by column chromatography to yield benzazocinones **27 a**–**27 c**.

**1‐Benzyl‐2‐oxo‐1,2,3,4,5,6‐hexahydrobenzo[*b*]azocine‐6‐carboxylic acid** ***N*** **‐[1‐methyl‐1‐(ethoxycarbonyl)ethyl]amide (27 a)**: According to GPD, HATU (209 mg, 550 μmol), DIPEA (71 mg, 0.55 mmol) and ethyl 2‐aminoisobutyrate (98 mg, 0.75 mmol) were converted with benzazocinone **26** (154 mg, 500 μmol) to yield the title compound **27 a** (183 mg, 433 μmol, 87 %) after chromatography (SiO_2_, hexanes/MTBE 1:7, *R*_f_=0.28) as a colorless oil. ^1^H NMR (500 MHz, CDCl_3_): *δ*=1.14 (s, 3 H), 1.19 (t, *J=*7.1 Hz, 3 H), 1.22 (s, 3 H), 1.28–1.38 (m, 1 H), 1.69–1.76 (m, 1 H), 1.84 (t, *J=*12.4 Hz, 1 H), 1.87–1.93 (m, 1 H), 2.27–2.34 (m, 2 H), 2.72 (d, *J=*10.8 Hz, 1 H), 4.04–4.13 (m, 3 H), 4.16 (d, *J=*13.9 Hz, 1 H), 5.94 (d, *J=*13.9 Hz, 1 H), 7.22–7.28 (m, 6 H), 7.32 (d, *J=*4.0 Hz, 2 H), 7.37 (d, *J=*7.9 Hz, 1 H) ppm. ^13^C{^1^H} NMR (125 MHz, CDCl_3_): *δ*=13.98 (CH_3_), 24.38 (CH_2_), 24.82 (CH_3_), 24.84 (CH_3_), 31.94 (CH_2_), 33.06 (CH_2_), 44.93 (CH), 51.98 (CH_2_), 55.64 (C), 60.93 (CH_2_), 125.60 (CH), 127.85 (CH), 128.07 (CH), 128.13 (CH), 128.52 (CH), 128.64 (2 CH), 129.26 (2 CH), 137.00 (C), 139.48 (C), 139.57 (C), 171.91 (C), 173.55 (C), 173.86 (C) ppm. IR (ATR): *ṽ*=3410 (w), 2983 (w), 2938 (w), 1737 (s), 1676 (s), 1651 (s), 1493 (s), 1452 (s), 1393 (m), 1383 (m), 1276 (s), 1234 (m), 1214 (m), 1193 (m), 1174 (s), 1148 (vs), 1029 (m), 920 (m), 759 (s), 733 (s), 705 (s), 635 (m) cm^−1^. HR‐MS (EI, 70 eV): calcd 422.2200 (for C_25_H_30_N_2_O_4_^+^), found 422.2196 [*M* ^+^]. C_25_H_30_N_2_O_4_ (422.53 g mol^−1^).

For the preparation of compounds **27 b** and **27 c** see the Supporting Information.

**1‐Benzyl‐2‐oxo‐1,2,3,4,5,6‐hexahydrobenzo[*b*]azocine‐6‐carboxamide (27 d)**: Pyridine (142 mg, 1.80 mmol) and Boc_2_O (327 mg, 1.50 mmol) were added to a solution of benzazocinone **26** (309 mg, 1.00 mmol) in 1,4‐dioxane (2 mL) and the resulting mixture was stirred at ambient temperature for 30 min. Then (NH_4_)_2_CO_3_ (269 mg, 2.80 mmol) was added and the reaction mixture was stirred at ambient temperature for 16 h. Subsequently, H_2_O (5 mL) and MTBE (5 mL) were added and the crude product **27 d** precipitated. It was filtered off, washed with MTBE (3×5 mL) and dried in vacuum to yield the title compound **27 d** (216 mg, 700 μmol, 70 %) as a colorless solid. M.p. 225–227 °C. ^1^H NMR (500 MHz, CDCl_3_): *δ*=1.39 (dddd, *J=*14.3 Hz, *J=*12.7 Hz, *J=*10.8 Hz, *J=*5.5 Hz, 1 H), 1.72–1.82 (m, 1 H), 1.87–1.96 (m, 2 H), 2.30–2.35 (m, 2 H), 2.72 (d, *J=*10.8 Hz, 1 H), 3.32 (br s, 1 H), 4.16 (d, *J=*13.7 Hz, 1 H), 4.89 (br s, 1 H), 6.05 (d, *J=*13.7 Hz, 1 H), 7.25–7.30 (m, 6 H), 7.36–7.40 (m, 3 H) ppm. ^13^C{^1^H} NMR (125 MHz, CDCl_3_): *δ*=24.42 (CH_2_), 31.56 (CH_2_), 33.12 (CH_2_), 44.66 (CH), 51.88 (CH_2_), 125.74 (CH), 127.27 (CH), 127.86 (CH), 128.24 (CH), 128.84 (3 CH), 129.93 (2 CH), 136.82 (C), 139.19 (C), 139.85 (C), 173.77 (C), 175.13 (C) ppm. IR (ATR): *ṽ*=3318 (m), 3132 (m), 2963 (w), 2928 (w), 1673 (s), 1628 (vs), 1595 (m), 1488 (m), 1450 (m), 1443 (m), 1427 (m), 1411 (m), 1392 (s), 1356 (m), 1333 (m), 1230 (m), 1203 (m), 1157 (m), 1020 (m), 994 (m), 776 (m), 759 (s), 727 (m), 715 (m), 694 (m), 670 (m), 637 (m), 579 (s) cm^−1^. HR‐MS (EI, 70 eV): calcd 308.1519 (for C_19_H_20_N_2_O_2_^+^), found 308.1511 [*M* ^+^]. C_19_H_20_N_2_O_2_ (308.38 g mol^−1^).

**1‐Benzyl‐6‐[(methoxycarbonyl)amino]‐2‐oxo‐1,2,3,4,5,6‐hexahydrobenzo[*b*]azocine (28)**: A solution of KOH (68 mg, 1.21 mmol) in MeOH (1 mL) was added at 0 °C to a solution of benzazocinone **27 d** (150 mg, 486 μmol) and PhI(OAc)_2_ (157 mg, 487 μmol) in CH_2_Cl_2_ (1 mL). The resulting mixture was stirred at 0 °C for 15 min and for further 16 h at ambient temperature. Subsequently, the reaction mixture was diluted with water (5 mL) and the aqueous layer was extracted with CH_2_Cl_2_ (3×5 mL). The combined organic layers were dried (MgSO_4_), filtered and the solvent was removed in vacuo. The crude product was purified by column chromatography (SiO_2_, hexanes/EtOAc/MeOH 1:1:0.1) to yield the title compound **28** (50 mg, 0.15 mmol, 30 %, *R*_f_=0.30) as a colorless solid. M.p. 114–130 °C. NMR spectra showed doubled and broadened signal sets due to *E*/*Z*‐isomers (ratio 1:0.15) at the carbamate C−N‐bond. ^1^H NMR (500 MHz, CDCl_3_), major isomer: *δ*=1.60 (qd, *J=*12.1 Hz, *J=*5.6 Hz, 1 H), 1.87–2.07 (m, 3 H), 2.11–2.14 (m, 1 H), 2.29–2.34 (m, 1 H), 3.60 (s, 3 H), 4.38 (br s, 1 H), 4.56 (br d, *J=*14.5 Hz, 1 H), 5.25 (br d, *J=*4.6 Hz, 1 H), 5.40 (br s, 1 H), 6.87 (br d, *J=*6.0 Hz, 1 H), 7.15 (br t, *J=*7.2 Hz, 1 H), 7.22–7.34 (m, 6 H), 7.39 (dd, *J=*7.9 Hz, *J=*1.4 Hz, 1 H) ppm; minor isomer: *δ*=1.73 (ddt, *J=*14.3 Hz, *J=*10.0 Hz, *J=*4.2 Hz, 1 H), 1.78–1.84 (m, 1 H), 1.87–2.07 (m, 2 H), 2.18 (dd, *J=*11.9 Hz, *J=*4.3 Hz, 1 H), 2.29–2.34 (m, 1 H), 3.58 (s, 3 H), 4.80 (d, *J=*14.2 Hz, 1 H), 4.95–4.99 (m, 1 H), 5.02 (d, *J=*14.2 Hz, 1 H), 7.01 (dd, *J=*7.7 Hz, *J=*1.3 Hz, 1 H), 7.22–7.34 (m, 8 H) ppm; a signal for the NH proton was not observed. ^13^C{^1^H} NMR (125 MHz, CDCl_3_), major isomer: *δ*=23.65 (CH_2_), 32.72 (CH_2_), 36.43 (CH_2_), 50.51 (CH), 52.06 (CH_3_), 52.40 (CH_2_), 125.25 (CH), 126.15 (CH), 127.27 (CH), 127.67 (CH), 128.37 (2 CH), 128.86 (CH), 128.88 (2 CH), 137.53 (C), 139.87 (C), 141.94 (C), 155.78 (C), 174.04 (C) ppm; minor isomer: *δ*=20.80 (CH_2_), 31.98 (CH_2_), 32.16 (CH_2_), 51.97 (CH_3_), 52.43 (CH_2_), 54.86 (CH), 127.32 (CH), 127.87 (CH), 128.33 (CH), 128.57 (CH), 128.68 (2 CH), 129.17 (2 CH), 131.75 (CH), 137.22 (C), 138.94 (C), 140.47 (C), 155.79 (C), 173.43 (C) ppm. IR (ATR): λ^−1^=3314 (m), 2929 (w), 1717 (s), 1627 (s), 1598 (m), 1521 (m), 1494 (m), 1454 (m), 1447 (m), 1406 (m), 1295 (m), 1247 (s), 1201 (m), 1058 (m), 1025 (m), 911 (m), 906 (m), 759 (m), 734 (s), 702 (s), 626 (m) cm^−1^. HR‐MS (EI, 70 eV): calcd 338.1625 (for C_20_H_22_N_2_O_3_^+^), found 338.1624 [*M* ^+^]. C_20_H_22_N_2_O_3_ (338.41 g mol^−1^).

## Conflict of interest

The authors declare no conflict of interest.

## Supporting information

As a service to our authors and readers, this journal provides supporting information supplied by the authors. Such materials are peer reviewed and may be re‐organized for online delivery, but are not copy‐edited or typeset. Technical support issues arising from supporting information (other than missing files) should be addressed to the authors.

Supplementary
