# Supplementary material for: Synthesis of Benzo[b]azocin‐2‐ones by Aryl Amination and Ring‐Expansion of α‐(Iodophenyl)‐β‐oxoesters
Source: Chemistry. 2019 Oct 28;25(65):14912–20. doi: 10.1002/chem.201903139 (PMC6899745; doi:10.1002/chem.201903139)
Supplement: Supplementary file 1 — Supplementary [file CHEM-25-14912-s001.pdf]

CHEMISTRY
A European Journal

Supporting Information

Synthesis of Benzo[*b*]azocin-2-ones by Aryl Amination and Ring-Expansion of α-(Iodophenyl)-β-oxoesters

Anna Dierks, Jan Tönjes, Marc Schmidtmann, and Jens Christoffers*[a]

chem_201903139_sm_miscellaneous_information.pdf

Supporting Information

Preparation of compounds **20b** and **20c**	S1
Conversion of β-oxoester **20a** with *n*-butylamine (products **18b**, **25b**)	S2
Conversion of β-oxoester **20a** with *n*-hexylamine (products **18c**, **24c**, **25c**)	S3
Conversion of β-oxoester **20a** with cyclohexylamine (products **18d**, **24d**)	S4
Conversion of β-oxoester **20a** with allylamine (products **18e**, **24e**)	S5
Conversion of β-oxoester **20a** with 2-ethoxyethylamine (product **18f**)	S6
Preparation of compounds **18j** and **18k**	S7
Preparation of compounds **27b** and **27c**	S8
^{1}H and ^{13}C{^{1}H} NMR spectra of all reported compounds	S10

Ethyl 1-(2-iodophenyl)-2-oxocyclohexane-1-carboxylate (20b).[25] According to GPA, TFAA (630 mg, 3.00 mmol), PIFA (1.12 g, 2.60 mmol) and β-oxoester **21b** (340 mg, 2.00 mmol) were converted in TFA (4 mL) and MeCN (4 mL) to furnish the title compound **20b** (468 mg, 1.26 mmol, 63%) after chromatography (SiO_2, hexanes/MTBE 5:1, R_f = 0.29) as a colorless oil. ^{1}H-NMR (300 MHz, $CDCl_3$): δ = 1.27 (t, J = 7.1 Hz, 3H), 1.75–1.91 (m, 2H), 1.97–2.07 (m, 2H), 2.58–2.85 (m, 4H), 4.20–4.37 (m, 2H), 6.96 (t, J = 7.5 Hz, 1H), 7.05 (d, J = 7.9 Hz, 1H), 7.32 (t, J = 7.6 Hz, 1H), 7.96 (d, J = 7.8 Hz, 1H) ppm. All spectroscopic data are in accordance with the literature.[25] $C_{15}H_{17}IO_3$ (372.20 g mol^{-1}).

Methyl 1-(2-iodophenyl)-2-oxocycloheptane-1-carboxylate (20c).[25] According to GPA, TFAA (315 mg, 1.50 mmol), PIFA (559 mg, 1.30 mmol) and β-oxoester **21c** (170 mg, 1.00 mmol) were converted in TFA (2 mL) and MeCN (2 mL) to furnish the the title compound **20c** (173 mg, 465 µmol, 47%) after chromatography (SiO_2, hexanes/MTBE 2:1, R_f = 0.43) as a colorless oil. ^{1}H-NMR (300 MHz, $CDCl_3$): 1.47–1.58 (m, 1H), 1.68–1.80 (m, 5H), 2.11–2.19 (m, 1H), 2.73–2.81 (m, 1H), 2.96–3.03 (m, 1H), 3.16–3.24 (m, 1H), 3.73 (s, 3H), 6.96 (t, J = 7.6 Hz, 1H), 7.02 (d, J = 7.7 Hz, 1H), 7.32 (t, J = 7.7 Hz, 1H), 7.94 (d, J = 7.8 Hz, 1H) ppm. All spectroscopic data are in accordance with the literature.[25] $C_{15}H_{17}IO_3$ (372.20 g mol^{-1}).

Conversion of β-oxoester 20a with *n*-butylamine. According to GPB, β-oxoester **20a** (179 mg, 500 µmol), K_3PO_4 (212 mg, 1.00 mmol) and CuI (14 mg, 75 µmol) were converted with *n*-butylamine (0.5 mL). The crude product was purified by column chromatography (SiO_2, hexanes/MTBE 1:1) to yield the benzazocinone **18b** (78 mg, 0.26 mmol, 51%, R_f = 0.33) as a colorless solid, mp 78 °C. As a second fraction, the acyclic amide **25b** (33 mg, 77 µmol, 15%, R_f = 0.17) was obtained as a colorless oil.

Ethyl 1-butyl-2-oxo-1,2,3,4,5,6-hexahydrobenzo[*b*]azocine-6-carboxylate (18b). ^{1}H-NMR (500 MHz, $CDCl_3$): δ = 0.87 (t, *J* = 7.3 Hz, 3H), 1.16 (t, *J* = 7.1 Hz, 3H), 1.24–1.37 (m, 2H), 1.40–1.49 (m, 1H), 1.50–1.65 (m, 2H), 1.76–1.85 (m, 2H), 1.89–1.94 (m, 1H), 2.23 (dd, *J* = 11.0 Hz, *J* = 7.8 Hz, 1H), 2.44 (dd, *J* = 13.4 Hz, *J* = 4.5 Hz, 1H), 3.26 (ddd, *J* = 13.3 Hz, *J* = 10.4 Hz, *J* = 5.0 Hz, 1H), 3.55 (d, *J* = 11.0 Hz, 1H), 4.08 (dq, *J* = 10.8 Hz, *J* = 7.1 Hz, 1H), 4.16 (dq, *J* = 10.8 Hz, *J* = 7.1 Hz, 1H), 4.38 (ddd, *J* = 13.3 Hz, *J* = 10.4 Hz, *J* = 5.9 Hz, 1H), 7.21–7.24 (m, 1H), 7.28–7.32 (m, 2H), 7.33–7.35 (m, 1H) ppm. ^{13}C{^{1}H}-NMR (125 MHz, $CDCl_3$): δ = 13.85 (CH_3), 13.98 (CH_3), 20.43 (CH_2), 24.27 (CH_2), 29.22 (CH_2), 31.95 (CH_2), 33.16 (CH_2), 44.78 (CH), 48.98 (CH_2), 61.88 (CH_2), 125.83 (CH), 126.87 (CH), 128.08 (CH), 128.41 (CH), 139.16 (C), 140.72 (C), 173.87 (C), 173.88 (C) ppm. IR (ATR): nu(tilde) = 2960 (w), 2937 (w), 2869 (w), 1734 (s), 1653 (vs), 1495 (m), 1456 (m), 1397 (m), 1302 (m), 1226 (m), 1187 (s), 1154 (m), 1102 (m), 1047 (m), 1028 (m), 767 (m), 741 (m) cm^{-1}. HR-MS (ESI): calcd. 310.1989 (for $C_{18}H_{25}LiNO_3^+$), found 310.1996 [M + Li^+]. $C_{18}H_{25}NO_3$ (303.40 g mol^{-1}).

Ethyl 6-(butylamino)-2-(2-iodophenyl)-6-oxohexanoate (25b). ^{1}H-NMR (500 MHz, $CDCl_3$): δ = 0.91 (t, *J* = 7.3 Hz, 3H), 1.20 (t, *J* = 7.1 Hz, 3H), 1.33 (sext, *J* = 7.3 Hz, 2H), 1.43–1.49 (m, 2H), 1.59–1.69 (m, 2H), 1.73–1.80 (m, 1H), 1.99–2.07 (m, 1H), 2.18 (t, *J* = 7.5 Hz, 2H), 3.23 (q, *J* = 7.0 Hz, 2H), 4.01 (t, *J* = 7.4 Hz, 1H), 4.07–4.18 (m, 2H), 5.53 (br s, 1H), 6.90–6.95 (m, 1H), 7.30–7.31 (m, 2H), 7.84 (d, *J* = 7.9 Hz, 1H) ppm. ^{13}C{^{1}H}-NMR (125 MHz, $CDCl_3$): δ = 13.73 (CH_3), 14.09 (CH_3), 20.05 (CH_2), 23.53 (CH_2), 31.66 (CH_2), 32.93 (CH_2), 36.37 (CH_2), 39.21 (CH_2), 54.68 (CH), 60.99 (CH_2), 101.62 (C), 127.67 (CH), 128.65 (CH), 128.83 (CH), 139.75 (CH), 141.89 (C), 172.17 (C), 173.27 (C) ppm. IR (ATR): nu(tilde) = 3300 (w), 2938 (w), 1731 (vs), 1640 (vs), 1548 (s), 1465 (m), 1369 (m), 1178 (m), 1149 (s), 1010 (s), 746

(s) cm^{-1}. HR-MS (EI, 70 eV): calcd. 431.0952 (for $C_{18}H_{26}INO_3^+$), found 431.0952 [M^+]. $C_{18}H_{26}INO_3$ (431.31 g mol^{-1}).

Conversion of β-oxoester 20a with *n*-hexylamine. According to GPB, β-oxoester **20a** (179 mg, 500 µmol), K_3PO_4 (212 mg, 1.00 mmol) and CuI (14 mg, 75 µmol) were converted with *n*-hexylamine (0.5 mL). The crude mixture was separated by column chromatography (SiO_2, hexanes/MTBE 1:2) to yield as the first fraction the benzofuran **23a** (4 mg, 0.02 mmol, 4%, R_f = 0.65) as a pale yellow oil. Secondly, the benzazocinone **18c** (83 mg, 0.25 mmol, 50%, R_f = 0.34) was eluted as a colorless oil. As third and fourth fractions, the acyclic amide **25c** (5 mg, 0.01 mmol, 2%, R_f = 0.21) and the acylic amide **24c** (26 mg, 78 µmol, 16%, R_f = 0.18) were obtained, both as a colorless oils.

Ethyl 1-hexyl-2-oxo-1,2,3,4,5,6-hexahydrobenzo[*b*]azocine-6-carboxylate (18c). ^{1}H-NMR (500 MHz, $CDCl_3$): δ = 0.80–0.82 (m, 3H), 1.14 (t, *J* = 7.1 Hz, 3H), 1.21–1.23 (m, 5H), 1.26–1.32 (m, 1H), 1.39–1.64 (m, 3H), 1.74–1.85 (m, 2H), 1.87–1.92 (m, 1H), 2.21 (dd, *J* = 11.0 Hz, *J* = 7.8 Hz, 1H), 2.40–2.44 (m, 1H), 3.23 (ddd, *J* = 13.2 Hz, *J* = 10.8 Hz, *J* = 4.9 Hz, 1H), 3.53 (d, *J* = 10.7 Hz, 1H), 4.06 (dq, *J* = 10.8 Hz, *J* = 7.1 Hz, 1H), 4.13 (dq, *J* = 10.8 Hz, *J* = 7.1 Hz, 1H), 4.35 (ddd, *J* = 13.2 Hz, *J* = 10.8 Hz, *J* = 5.8 Hz, 1H), 7.19–7.22 (m, 1H), 7.26–7.33 (m, 3H) ppm. ^{13}C{^{1}H}-NMR (125 MHz, $CDCl_3$): δ = 13.92 (2 CH_3), 22.36 (CH_2), 24.21 (CH_2), 26.70 (CH_2), 26.90 (CH_2), 31.51 (CH_2), 31.92 (CH_2), 33.09 (CH_2), 44.72 (CH), 49.17 (CH_2), 60.82 (CH_2), 125.81 (CH), 126.80 (CH), 128.03 (CH), 128.36 (CH), 139.10 (C), 140.65 (C), 173.74 (C), 173.83 (C) ppm. IR (ATR): nu(tilde) = 2931 (w), 2864 (w), 1733 (s), 1652 (vs), 1493 (m), 1454 (m), 1396 (m), 1300 (m), 1224 (m), 1183 (s), 1150 (m), 1098 (m), 1046 (m), 1025 (m), 765 (m), 738 (m) cm^{-1}. HR-MS (EI, 70 eV): calcd. 331.2142 (for $C_{20}H_{29}NO_3^+$), found 310.2140 [M^+]. $C_{20}H_{29}NO_3$ (331.46 g mol^{-1}).

Ethyl 6-(hexylamino)-2-(2-iodophenyl)-6-oxohexanoate (25c). ^{1}H-NMR (500 MHz, $CDCl_3$): δ = 0.88 (t, *J* = 7.0 Hz, 3H), 1.21 (t, *J* = 7.1 Hz, 3H), 1.28–1.33 (m, 6H), 1.45–1.49 (m, 2H), 1.62–1.68 (m, 2H), 1.73–1.80 (m, 1H), 2.00–2.07 (m, 1H), 2.19 (t, *J* = 7.5 Hz, 2H), 3.22 (q, *J* = 7.0 Hz, 2H), 4.02 (dd, *J* = 8.1 Hz, *J* = 6.7 Hz, 1H), 4.08–4.19 (m, 2H), 5.49 (br s, 1H), 6.91–6.96 (m, 1H), 7.30–7.31 (m, 2H), 7.85 (d, *J* = 7.9 Hz, 1H) ppm. ^{13}C{^{1}H}-NMR (125 MHz, $CDCl_3$): δ = 14.02 (CH_3), 14.10 (CH_3), 22.55

(CH_2), 23.55 (CH_2), 26.59 (CH_2), 29.58 (CH_2), 31.47 (CH_2), 32.94 (CH_2), 36.41 (CH_2), 39.53 (CH_2), 54.70 (CH), 61.01 (CH_2), 101.64 (C), 127.69 (CH), 128.67 (CH), 128.85 (CH), 139.77 (CH), 141.91 (C), 172.16 (C), 173.30 (C) ppm. IR (ATR): nu(tilde) = 3276 (w), 2929 (s), 2861 (m), 1733 (vs), 1642 (vs), 1549 (s), 1465 (s), 1437 (m), 1370 (m), 1178 (m), 1150 (s), 1010 (s), 747 (s) cm^{-1}. HR-MS (EI, 70 eV): calcd. 459.1265 (for $C_{20}H_{30}INO_3^+$), found 459.1262 [M^+]. $C_{20}H_{30}INO_3$ (459.37 g mol^{-1}).

Ethyl 6-(hexylamino)-6-oxo-2-phenylhexanoate (24c). ^{1}H-NMR (500 MHz, $CDCl_3$): δ = 0.86–0.88 (m, 3H), 1.19 (t, J = 7.1 Hz, 3H), 1.25–1.32 (m, 6H), 1.43–1.49 (m, 2H), 1.53–1.66 (m, 2H), 1.73–1.82 (m, 1H), 2.03–2.12 (m, 1H), 2.12–2.17 (m, 2H), 3.19–3.23 (m, 2H), 3.52 (t, J = 7.6 Hz, 1H), 4.07 (dq, J = 10.8 Hz, J = 7.1 Hz, 1H), 4.14 (dq, J = 10.8 Hz, J = 7.1 Hz, 1H), 5.48 (br s, 1H), 7.22–7.32 (m, 5H) ppm. $^{13}C\{^1H\}$-NMR (125 MHz, $CDCl_3$): δ = 13.99 (CH_3), 14.09 (CH_3), 22.52 (CH_2), 23.72 (CH_2), 26.56 (CH_2), 29.55 (CH_2), 31.44 (CH_2), 33.05 (CH_2), 36.39 (CH_2), 39.50 (CH_2), 51.55 (CH), 60.77 (CH_2), 127.20 (CH), 127.80 (2 CH), 128.59 (2 CH), 138.94 (C), 172.22 (C), 173.88 (C) ppm. IR (ATR): nu(tilde) = 3294 (w), 2954 (m), 2927 (m), 2857 (m), 1732 (vs), 1641 (vs), 1549 (m), 1455 (m), 1175 (m), 1148 (m), 1026 (m), 732 (m), 698 (s) cm^{-1}. HR-MS (EI, 70 eV): calcd. 333.2298 (for $C_{20}H_{31}NO_3^+$), found 333.2297 [M^+]. $C_{20}H_{31}NO_3$ (333.47 g mol^{-1}).

Conversion of β-oxoester 20a with cyclohexylamine. According to GPB, β-oxo-ester **20a** (179 mg, 500 µmol), K_3PO_4 (212 mg, 1.00 mmol) and CuI (14 mg, 75 µmol) were converted with cyclohexylamine (0.5 mL). The crude mixture was separated by column chromatography (SiO_2, hexanes/MTBE 1:2) to yield the benzofuran **23a** (13 mg, 56 µmol, 11%, R_f = 0.65) as a pale yellow oil in the first fraction. Secondly, the benzazocinone **18d** (83 mg, 0.25 mmol, 50%, R_f = 0.31) was eluted as as a colorless solid, mp 120–123 °C. As third fraction, the acyclic amide **24d** (22 mg, 66 µmol, 13%, R_f = 0.19) was obtained as a colorless oil.

Ethyl 1-cyclohexyl-2-oxo-1,2,3,4,5,6-hexahydrobenzo[*b*]azocine-6-carboxylate (18d). ^{1}H-NMR (500 MHz, $CDCl_3$): δ = 0.97–1.05 (m, 2H), 1.17 (t, J = 7.1 Hz, 3H), 1.33–1.39 (m, 1H), 1.43–1.53 (m, 3H), 1.55–1.60 (m, 1H), 1.68–1.70 (m, 2H), 1.77–1.84 (m, 3H), 1.86–1.92 (m, 1H), 2.14–2.23 (m, 2H), 2.40–2.44 (m, 1H), 3.63 (d, J = 11.1 Hz, 1H), 4.09 (dq, J = 10.7 Hz, J = 7.2 Hz, 1H), 4.16 (dq, J = 10.7 Hz, J = 7.2

Hz, 1H), 4.56 (tt, J = 12.0 Hz, J = 3.5 Hz, 1H), 7.20–7.21 (m, 1H), 7.27–7.30 (m, 1H), 7.32–7.37 (m, 2H) ppm. $^{13}C\{^1H\}$-NMR (125 MHz, $CDCl_3$): δ = 13.89 (CH_3), 24.43 (CH_2), 25.45 (CH_2), 25.80 (CH_2), 25.92 (CH_2), 29.97 (CH_2), 32.15 (CH_2), 32.78 (CH_2), 33.57 (CH_2), 44.86 (CH), 55.36 (CH), 60.77 (CH_2), 126.81 (CH), 127.30 (CH), 127.82 (CH), 128.85 (CH), 138.30 (C), 140.51 (C), 173.77 (C), 173.84 (C) ppm. IR (ATR): nu(tilde) = 2930 (m), 2856 (w), 1732 (vs), 1645 (vs), 1492 (m), 1446 (m), 1369 (m), 1302 (m), 1222 (m), 1186 (s), 1143 (m), 1024 (m), 769 (m), 745 (m) cm^{-1}. HR-MS (EI, 70 eV): calcd. 329.1985 (for $C_{20}H_{27}NO_3^+$), found 329.1985 [M^+]. $C_{20}H_{27}NO_3$ (329.44 g mol^{-1}).

Ethyl 6-(cyclohexylamino)-6-oxo-2-phenylhexanoate (24d). ^1H-NMR (500 MHz, $CDCl_3$): δ = 1.04–1.15 (m, 3H), 1.19 (t, J = 7.1 Hz, 3H), 1.29–1.39 (m, 2H), 1.55–1.62 (m, 3H), 1.67–1.70 (m, 2H), 1.74–1.81 (m, 1H), 1.87–1.89 (m, 2H), 2.03–2.10 (m, 1H), 2.13 (t, J = 7.9 Hz, 2H), 3.52 (t, J = 7.6 Hz, 1H), 3.70–3.77 (m, 1H), 4.07 (dq, J = 10.8 Hz, J = 7.1 Hz, 1H), 4.14 (dq, J = 10.8 Hz, J = 7.1 Hz, 1H), 5.35 (br d, J = 7.6 Hz, 1H), 7.22–7.26 (m, 1H), 7.27–7.32 (m, 4H) ppm. $^{13}C\{^1H\}$-NMR (125 MHz, $CDCl_3$): δ = 14.08 (CH_3), 23.75 (CH_2), 24.84 (2 CH_2), 25.49 (CH_2), 32.99 (CH_2), 33.18 (2 CH_2), 36.53 (CH_2), 48.08 (CH), 51.54 (CH), 60.76 (CH_2), 127.20 (CH), 127.80 (2 CH), 128.59 (2 CH), 138.95 (C), 171.36 (C), 173.88 (C) ppm. IR (ATR): nu(tilde) = 3288 (w), 2929 (s), 2853 (m), 1731 (vs), 1637 (vs), 1544 (s), 1451 (m), 1146 (s), 1027 (m), 733 (m), 698 (s) cm^{-1}. HR-MS (EI, 70 eV): calcd. 331.2142 (for $C_{20}H_{29}NO_3^+$), found 331.2135 [M^+]. $C_{20}H_{29}NO_3$ (331.46 g mol^{-1}).

Conversion of β-oxoester 20a with allylamine. According to GPB, β-oxoester **20a** (179 mg, 500 µmol), K_3PO_4 (212 mg, 1.00 mmol) and CuI (14 mg, 75 µmol) were converted with allylamine (0.5 mL). The crude product was purified by column chromatography (SiO_2, hexanes/MTBE 1:2) to yield the benzazocinone **18e** (70 mg, 0.24 mmol, 49%, R_f = 0.28) as a colorless oil. As second fraction, the acyclic amide **24e** (30 mg, 0.10 mmol, 20%, R_f = 0.12) was obtained as a colorless oil.

Ethyl 1-allyl-2-oxo-1,2,3,4,5,6-hexahydrobenzo[*b*]azocine-6-carboxylate (18e). ^1H-NMR (500 MHz, $CDCl_3$): δ = 1.17 (t, J = 7.0 Hz, 3H), 1.51–1.60 (m, 1H), 1.77–1.94 (m, 3H), 2.25–2.29 (m, 1H), 2.42–2.46 (m, 1H), 3.52 (d, J = 11.2 Hz, 1H), 4.05–4.18 (m, 3H), 4.76 (dd, J = 14.4 Hz, J = 6.3 Hz, 1H), 5.10 (d, J = 10.1 Hz, 1H), 5.15

(d, J = 17.1 Hz, 1H), 5.86–5.94 (m, 1H), 7.20–7.33 (m, 4H) ppm. $^{13}C\{^{1}H\}$-NMR (125 MHz, $CDCl_3$): δ = 13.96 (CH_3), 24.28 (CH_2), 32.01 (CH_2), 33.06 (CH_2), 44.78 (CH), 51.74 (CH_2), 60.84 (CH_2), 118.78 (CH_2), 125.96 (CH), 126.95 (CH), 127.99 (CH), 128.57 (CH), 132.19 (CH), 139.04 (C), 140.48 (C), 173.81 (C), 173.82 (C) ppm. IR (ATR): nu(tilde) = 2979 (w), 2935 (w), 1731 (vs), 1651 (vs), 1493 (m), 1454 (m), 1389 (m), 1225 (m), 1185 (s), 1150 (m), 1098 (m), 765 (m), 739 (m) cm^{-1}. HR-MS (ESI): calcd. 287.1516 (for $C_{17}H_{21}NO_3^{+}$), found 287.1518 [M^{+}]. $C_{17}H_{21}NO_3$ (287.36 g mol^{-1}).

Ethyl 6-(allylamino)-6-oxo-2-phenylhexanoate (24e). ^{1}H-NMR (500 MHz, $CDCl_3$): δ = 1.16 (t, J = 7.1 Hz, 3H), 1.51–1.65 (m, 2H), 1.77 (dddd, J = 13.3 Hz, J = 10.2 Hz, J = 7.3 Hz, J = 5.8 Hz, 1H), 2.05 (dddd, J = 13.3 Hz, J = 10.5 Hz, J = 7.9 Hz, J = 5.5 Hz, 1H), 2.13–2.19 (m, 2H), 3.50 (t, J = 7.6 Hz, 1H), 3.82 (tt, J = 5.7 Hz, J = 1.4 Hz, 2H), 4.01–4.14 (m, 2H), 5.08 (dq, J = 10.2 Hz, J = 1.4 Hz, 1H), 5.12 (dq, J = 17.2 Hz, J = 1.6 Hz, 1H), 5.59 (br s, 1H), 5.78 (ddt, J = 17.2 Hz, J = 10.2 Hz, J = 5.7 Hz, 1H), 7.19–7.29 (m, 5H) ppm. $^{13}C\{^{1}H\}$-NMR (125 MHz, $CDCl_3$): δ = 14.06 (CH_3), 23.63 (CH_2), 33.01 (CH_2), 36.23 (CH_2), 41.86 (CH_2), 51.53 (CH), 60.75 (CH_2), 116.33 (CH_2), 127.20 (CH), 127.80 (2 CH), 128.58 (2 CH), 134.21 (CH), 138.89 (C), 172.16 (C), 173.83 (C) ppm. IR (ATR): nu(tilde) = 3288 (w), 2929 (w), 1731 (vs), 1642 (vs), 1547 (s), 1457 (m), 1372 (m), 1269 (m), 1174 (s), 1152 (vs), 1026 (m), 920 (m), 734 (m), 700 (vs) cm^{-1}. HR-MS (EI, 70 eV): calcd. 289.1672 (for $C_{17}H_{23}NO_3^{+}$), found 289.1669 [M^{+}]. $C_{17}H_{23}NO_3$ (289.38 g mol^{-1}).

Ethyl 1-(2-ethoxyethyl)-2-oxo-1,2,3,4,5,6-hexahydrobenzo[*b*]azocine-6-carboxylate (18f). According to GPB, β-oxoester **20a** (179 mg, 500 µmol), K_3PO_4 (212 mg, 1.00 mmol) and CuI (14 mg, 75 µmol) were converted with 2-ethoxyethylamine (0.5 mL). The crude product was purified by column chromatography (SiO_2, hexanes/MTBE 5:1) to yield the benzazocinone **18f** (60 mg, 0.19 mmol, 38%, R_f = 0.26) as a colorless oil. ^{1}H-NMR (500 MHz, $CDCl_3$): δ = 1.12 (t, J = 7.0 Hz, 3H), 1.17 (t, J = 7.1 Hz, 3H), 1.53 (dddd, J = 14.3 Hz, J = 12.3 Hz, J = 11.1 Hz, J = 5.6 Hz, 1H), 1.75–1.95 (m, 3H), 2.24 (dd, J = 11.4 Hz, J = 8.1 Hz, 1H), 2.44–2.48 (m, 1H), 3.41–3.47 (m, 2H), 3.50 (ddd, J = 9.2 Hz, J = 5.9 Hz, J = 4.4 Hz, 1H), 3.53–3.57 (m, 1H), 3.61 (ddd, J = 9.2 Hz, J = 7.6 Hz, J = 5.5 Hz, 1H), 3.67 (dd, J = 11.1 Hz, J = 1.0 Hz, 1H), 4.08–4.18 (m, 2H), 4.49 (ddd, J = 13.4 Hz, J = 7.6 Hz, J = 5.9 Hz, 1H), 7.29–7.31 (m, 3H), 7.31–7.35 (m, 1H) ppm. $^{13}C\{^{1}H\}$-NMR (125 MHz, $CDCl_3$): δ = 14.02 (CH_3), 15.01

(CH_3), 24.34 (CH_2), 32.14 (CH_2), 33.06 (CH_2), 44.54 (CH), 48.44 (CH_2), 60.84 (CH_2), 66.11 (CH_2), 66.68 (CH_2), 126.10 (CH), 127.05 (CH), 128.05 (CH), 128.47 (CH), 139.15 (C), 140.74 (C), 173.95 (C), 174.36 (C) ppm. IR (ATR): nu(tilde) = 2972 (w), 2942 (w), 2864 (w), 1730 (s), 1653 (vs), 1494 (m), 1454 (m), 1392 (m), 1300 (m), 1225 (m), 1186 (m), 1113 (s), 1045 (m), 1026 (m), 766 (m), 735 (m) cm^{-1}. HR-MS (EI, 70 eV): calcd. 319.1778 (for $C_{18}H_{25}NO_4^+$), found 319.1772 [M^+]. $C_{18}H_{25}NO_4$ (319.40 g mol^{-1}).

Ethyl [1-(methoxycarbonyl)methyl]-2-oxo-1,2,3,4,5,6-hexahydrobenzo[*b*]azocine-6-carboxylate (18j). According to GPC, benzazocinone **18g** (124 mg, 500 µmol), *n*BuLi (0.21 mL, 2.5 mol L^{-1} in hexanes, 0.53 mmol) and *i*Pr_2NH (54 mg, 0.53 mmol) were converted with methyl bromoacetate (81 mg, 0.53 mmol) to yield the title compound **18j** (126 mg, 395 µmol, 79%) after chromatography (SiO_2, hexanes/MTBE 1:5, R_f = 0.28) as a colorless solid, mp 100–104 °C. ^{1}H-NMR (500 MHz, $CDCl_3$): δ = 1.16 (t, J = 7.1 Hz, 3H), 1.53 (dddd, J = 14.3 Hz, J = 12.9 Hz, J = 11.1 Hz, J = 5.4 Hz, 1H), 1.80 (td, J = 12.9 Hz, J = 5.4 Hz, 1H), 1.88–1.95 (m, 2H), 2.27 (dd, J = 12.3 Hz, J = 8.2 Hz, 1H), 2.47 (dd, J = 14.3 Hz, J = 5.4 Hz, 1H), 3.71 (s, 3H), 4.01 (dd, J = 11.1 Hz, J = 0.9 Hz, 1H), 4.06–4.17 (m, 2H), 4.35 (d, J = 17.0 Hz, 1H), 4.58 (d, J = 17.0 Hz, 1H), 7.24–7.30 (m, 3H), 7.33–7.35 (m, 1H) ppm. $^{13}C\{^1H\}$-NMR (125 MHz, $CDCl_3$): δ = 13.94 (CH_3), 24.19 (CH_2), 32.15 (CH_2), 32.53 (CH_2), 44.44 (CH), 50.98 (CH_2), 52.07 (CH_3), 60.78 (CH_2), 125.28 (CH), 127.49 (CH), 128.12 (CH), 128.68 (CH), 138.71 (C), 140.83 (C), 169.28 (C), 173.87 (C), 174.31 (C) ppm. IR (ATR): nu(tilde) = 2973 (w), 2952 (w), 1757 (s), 1716 (vs), 1649 (vs), 1496 (m), 1441 (m), 1384 (m), 1220 (s), 1196 (vs), 1095 (m), 1025 (m), 977 (m), 781 (m), 742 (m), 722 (m) cm^{-1}. HR-MS (EI, 70 eV): calcd. 319.1414 (for $C_{17}H_{21}NO_5^+$), found 319.1426 [M^+]. $C_{17}H_{21}NO_5$ (319.36 g mol^{-1}).

Ethyl 1-[1-(ethoxycarbonyl)ethyl]-2-oxo-1,2,3,4,5,6-hexahydrobenzo[*b*]azocine-6-carboxylate (18k). According to GPC, benzazocinone **18g** (124 mg, 500 µmol) in abs. THF (1 mL), *n*BuLi (0.21 mL, 2.5 mol L^{-1} in hexanes, 0.53 mmol) and *i*Pr_2NH (54 mg, 0.53 mmol) were converted with ethyl 2-bromopropionate (96 mg, 0.53 mmol) to yield in a first fraction the title compound **18k** (59 mg, 0.17 mmol, 34%, R_f = 0.32) after chromatography (SiO_2, hexanes/EtOAc 1:1) as a colorless oil. The product **18k** was isolated as a mixture of two diastereomers (ratio 87:13 by ^{1}H-NMR). In the se-

cond fraction, starting material **18g** (45 mg, 0.18 mmol, 36%, R_f = 0.11) was recovered. ^{1}H-NMR (500 MHz, $CDCl_3$), major diastereomer: δ = 1.06–1.11 (m, 3H), 1.19–1.25 (m, 6H), 1.42–1.53 (m, 1H), 1.65–1.77 (m, 1H), 1.80–1.89 (m, 2H), 2.18–2.23 (m, 1H), 2.35–2.39 (m, 1H), 3.64 (dd, J = 11.1 Hz, J = 6.5 Hz, 1H), 4.00–4.09 (m, 2H), 4.13–4.23 (m, 2H), 4.96–5.02 (m, 1H), 7.17–7.22 (m, 1H), 7.23–7.28 (m, 2H), 7.38–7.41 (m, 1H) ppm; minor diastereomer: δ = 1.06–1.11 (m, 3H), 1.19–1.25 (m, 3H), 1.42–1.53 (m, 4H), 1.65–1.89 (m, 3H), 2.11–2.17 (m, 1H), 2.35–2.39 (m, 1H), 3.74 (dd, J = 11.0 Hz, J = 6.7 Hz, 1H), 4.00–4.09 (m, 2H), 4.13–4.23 (m, 3H), 7.17–7.22 (m, 1H), 7.23–7.28 (m, 3H) ppm. ^{13}C{^{1}H}-NMR (125 MHz, $CDCl_3$), major diastereomer: δ = 13.96 (CH_3), 14.15 (CH_3), 15.02 (CH_3), 24.31 (CH_2), 32.01 (CH_2), 33.13 (CH_2), 44.69 (CH), 54.86 (CH), 60.87 (CH_2), 61.28 (CH_2), 126.90 (2 CH), 127.81 (CH), 129.11 (CH), 138.45 (C), 139.88 (C), 172.20 (C), 173.76 (C), 174.26 (C) ppm; minor diastereomer: δ = 13.96 (CH_3), 14.30 (CH_3), 14.91 (CH_3), 24.13 (CH_2), 32.05 (CH_2), 33.06 (CH_2), 44.80 (CH), 59.64 (CH), 60.91 (CH_2), 61.20 (CH_2), 126.53 (CH), 126.96 (CH), 128.21 (CH), 128.85 (CH), 139.27 (C), 140.46 (C), 171.01 (C), 173.41 (C), 173.82 (C) ppm. IR (ATR): nu(tilde) = 2978 (w), 2941 (w), 1731 (vs), 1656 (vs), 1494 (m), 1452 (m), 1371 (m), 1303 (m), 1226 (m), 1186 (s), 1090 (m), 1049 (m), 1022 (m), 768 (m), 738 (m) cm^{-1}. HR-MS (EI, 70 eV): calcd. 347.1727 (for $C_{19}H_{25}NO_5^+$), found 347.1734 [M^+]. $C_{19}H_{25}NO_5$ (347.41 g mol^{-1}).

1-Benzyl-2-oxo-1,2,3,4,5,6-hexahydrobenzo[*b*]azocine-6-carboxylic acid *N*-[2-(ethoxycarbonyl)ethyl]amide (27b). According to GPD, HATU (209 mg, 550 µmol), DIPEA (142 mg, 1.10 mmol) and β-alanine ethyl ester-hydrochloride (115 mg, 749 µmol) were converted with benzazocinone **26** (154 mg, 500 µmol) to yield the title compound **27b** (173 mg, 424 µmol, 85%) after chromatography (SiO_2, hexanes/EtOAc 1:3, R_f = 0.33) as a colorless oil. ^{1}H-NMR (500 MHz, $CDCl_3$): δ = 1.20 (t, J = 7.1 Hz, 3H), 1.37 (dddd, J = 14.3 Hz, J = 12.6 Hz, J = 10.9 Hz, J = 5.5 Hz, 1H), 1.73 (dtdd, J = 14.6 Hz, J = 12.7 Hz, J = 5.5 Hz, J = 1.8 Hz, 1H), 1.83–1.93 (m, 2H), 2.26–2.32 (m, 4H), 2.57 (dd, J = 10.9 Hz, J = 0.9 Hz, 1H), 2.98 (dq, J = 13.1 Hz, J = 7.0 Hz, 1H), 3.18 (dq, J = 13.1 Hz, J = 6.6 Hz, 1H), 3.62 (br t, J = 5.8 Hz, 1H), 3.99–4.09 (m, 2H), 4.13 (d, J = 13.7 Hz, 1H), 6.00 (d, J = 13.7 Hz, 1H), 7.22–7.27 (m, 4H), 7.28–7.31 (m, 3H), 7.31–7.36 (m, 2H) ppm. ^{13}C{^{1}H}-NMR (125 MHz, $CDCl_3$): δ = 14.14 (CH_3), 24.42 (CH_2), 31.54 (CH_2), 33.11 (CH_2), 33.70 (CH_2), 35.11 (CH_2), 45.19 (CH), 51.93 (CH_2), 60.38 (CH_2), 125.76 (CH), 127.20 (CH), 127.77 (CH), 128.09

(CH), 128.76 (CH), 128.83 (2 CH), 129.99 (2 CH), 136.79 (C), 139.22 (C), 140.20 (C), 171.43 (C), 172.98 (C), 173.79 (C) ppm. IR (ATR): nu(tilde) = 3412 (w), 3031 (w), 2939 (w), 2865 (w), 1731 (s), 1675 (s), 1650 (vs), 1513 (m), 1492 (m), 1452 (m), 1393 (m), 1181 (s), 1152 (m), 844 (m), 759 (m), 735 (m), 705 (s), 634 (m) cm^{-1}. HR-MS (EI, 70 eV): calcd. 408.2044 (for $C_{24}H_{28}N_2O_4^+$), found 408.2039 [M^+]. $C_{24}H_{28}N_2O_4$ (408.50 g mol^{-1}).

1-Benzyl-2-oxo-1,2,3,4,5,6-hexahydrobenzo[*b*]azocine-6-carboxylic acid *N*-(2,2,2-trifluoroethyl)amide (27c). According to GPD, HATU (209 mg, 550 µmol), DIPEA (71 mg, 0.55 mmol) and 2,2,2-trifluoroethylamine (74 mg, 0.75 mmol) were converted with benzazocinone **26** (154 mg, 500 µmol) to yield the title compound **27c** (172 mg, 441 µmol, 88%) after chromatography (SiO_2, hexanes/EtOAc 1:1, R_f = 0.29) as a colorless solid, mp 127–128 °C. ^{1}H-NMR (500 MHz, $CDCl_3$): δ = 1.40 (dddd, *J* = 14.4 Hz, *J* = 12.8 Hz, *J* = 10.9 Hz, *J* = 5.5 Hz, 1H), 1.70–1.79 (m, 1H), 1.85–1.94 (m, 2H), 2.30–2.34 (m, 2H), 2.65 (dd, *J* = 10.9 Hz, *J* = 0.9 Hz, 1H), 3.25 (dqd, *J* = 14.8 Hz, *J* = 9.0 Hz, *J* = 5.8 Hz, 1H), 3.42 (br t, *J* = 6.6 Hz, 1H), 3.67 (dqd, *J* = 14.8 Hz, *J* = 9.1 Hz, *J* = 7.5 Hz, 1H), 4.09 (d, *J* = 13.7 Hz, 1H), 6.08 (d, *J* = 13.7 Hz, 1H), 7.21 (dd, *J* = 7.9 Hz, *J* = 1.1 Hz, 1H), 7.25–7.28 (m, 6H), 7.35–7.40 (m, 2H) ppm. ^{13}C{^{1}H}-NMR (125 MHz, $CDCl_3$): δ = 24.31 (CH_2), 31.45 (CH_2), 33.06 (CH_2), 40.20 (q, $^2J_{CF}$ = 34.8 Hz, CH_2), 45.23 (CH), 51.79 (CH_2), 123.68 (q, $^1J_{CF}$ = 279.1 Hz, C), 125.83 (CH), 127.16 (CH), 127.57 (CH), 128.47 (CH), 128.83 (2 CH), 128.92 (CH), 130.09 (2 CH), 136.87 (C), 139.08 (C), 139.30 (C), 173.08 (C), 173.64 (C) ppm. ^{19}F{^{1}H}-NMR (470 MHz, $CDCl_3$): δ = –72.10 (s, CF_3) ppm. IR (ATR): nu(tilde) = 3410 (m), 2969 (w), 2948 (w), 2865 (w), 1690 (s), 1644 (vs), 1598 (m), 1514 (m), 1493 (m), 1454 (m), 1438 (m), 1392 (s), 1276 (s), 1228 (m), 1195 (m), 1162 (s), 1144 (vs), 1083 (m), 989 (m), 833 (m), 780 (m), 764 (s), 733 (s), 711 (s), 664 (m), 633 (s) cm^{-1}. HR-MS (EI, 70 eV): calcd. 390.1550 (for $C_{21}H_{21}F_3N_2O_2^+$), found 390.1541 [M^+]. $C_{21}H_{21}F_3N_2O_2$ (390.41 g mol^{-1}).

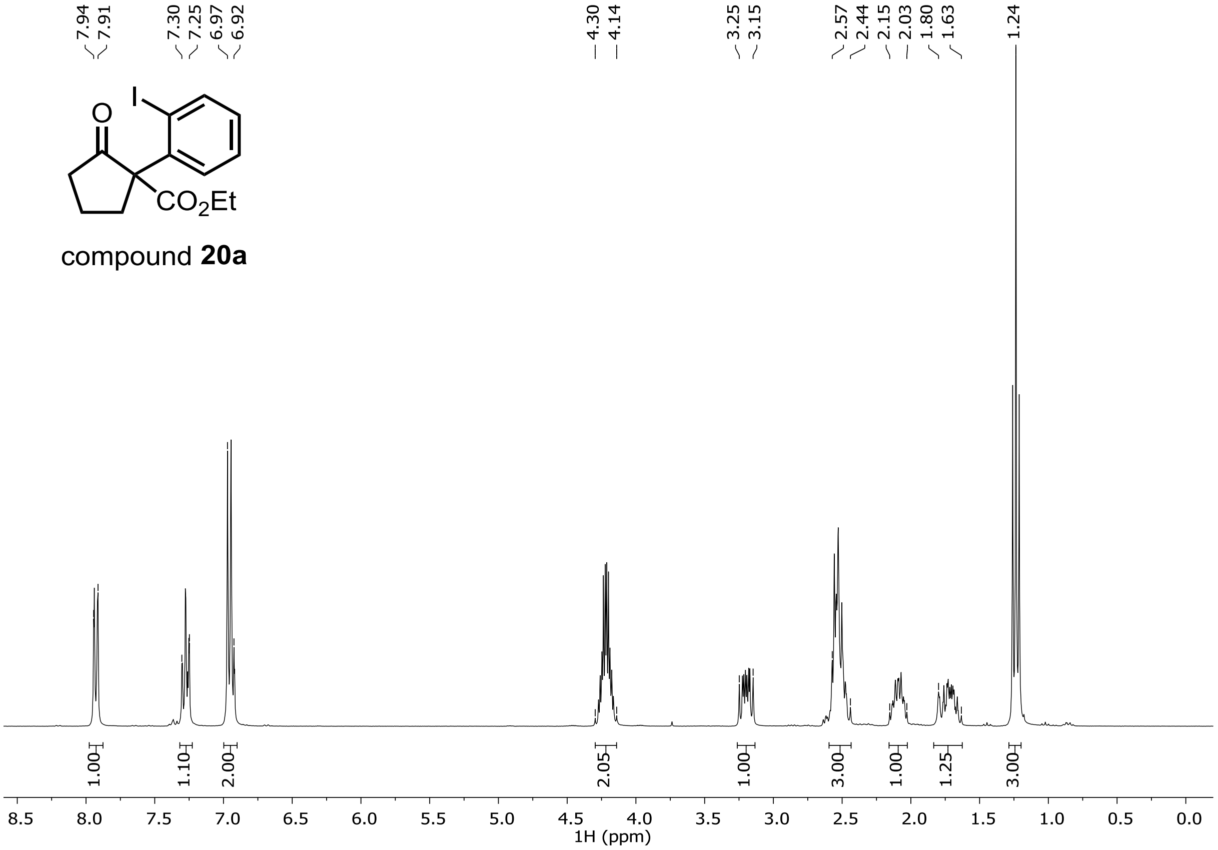

compound **20a**

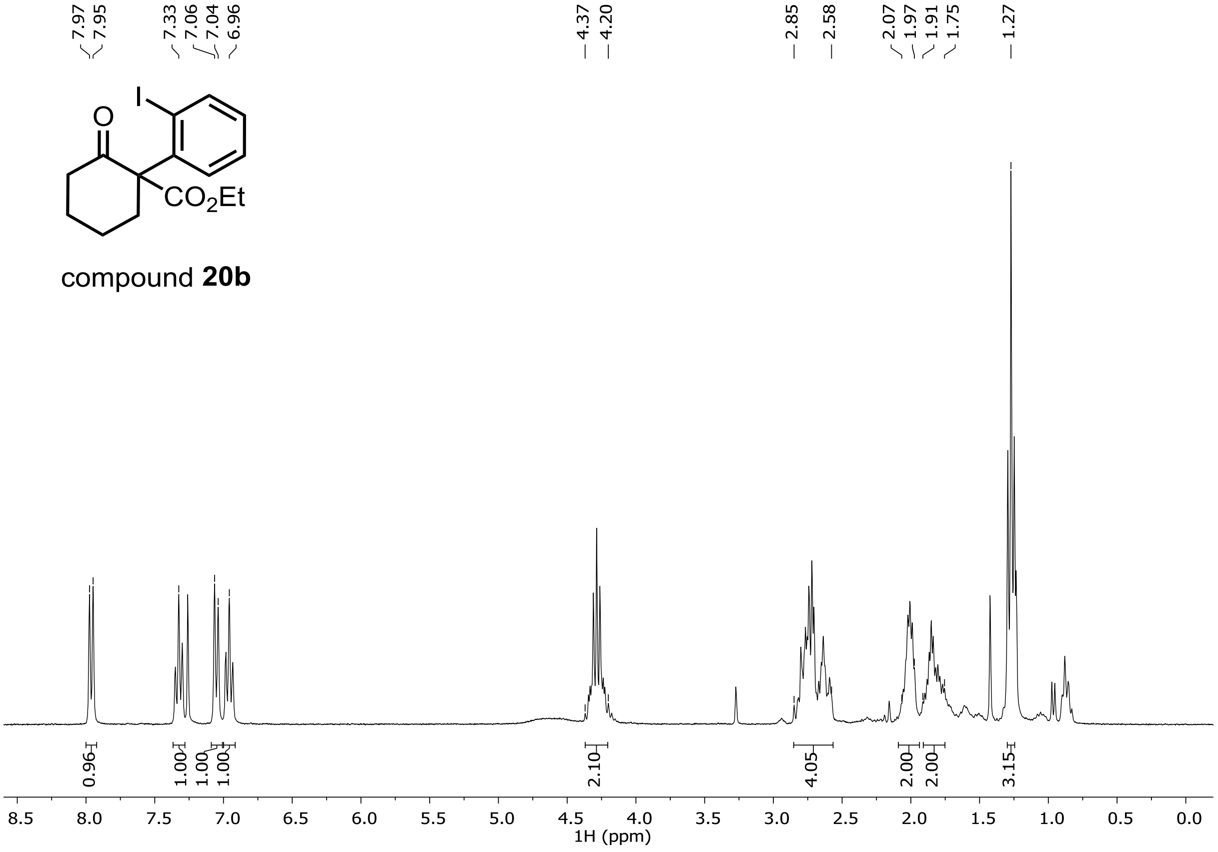

compound **20b**

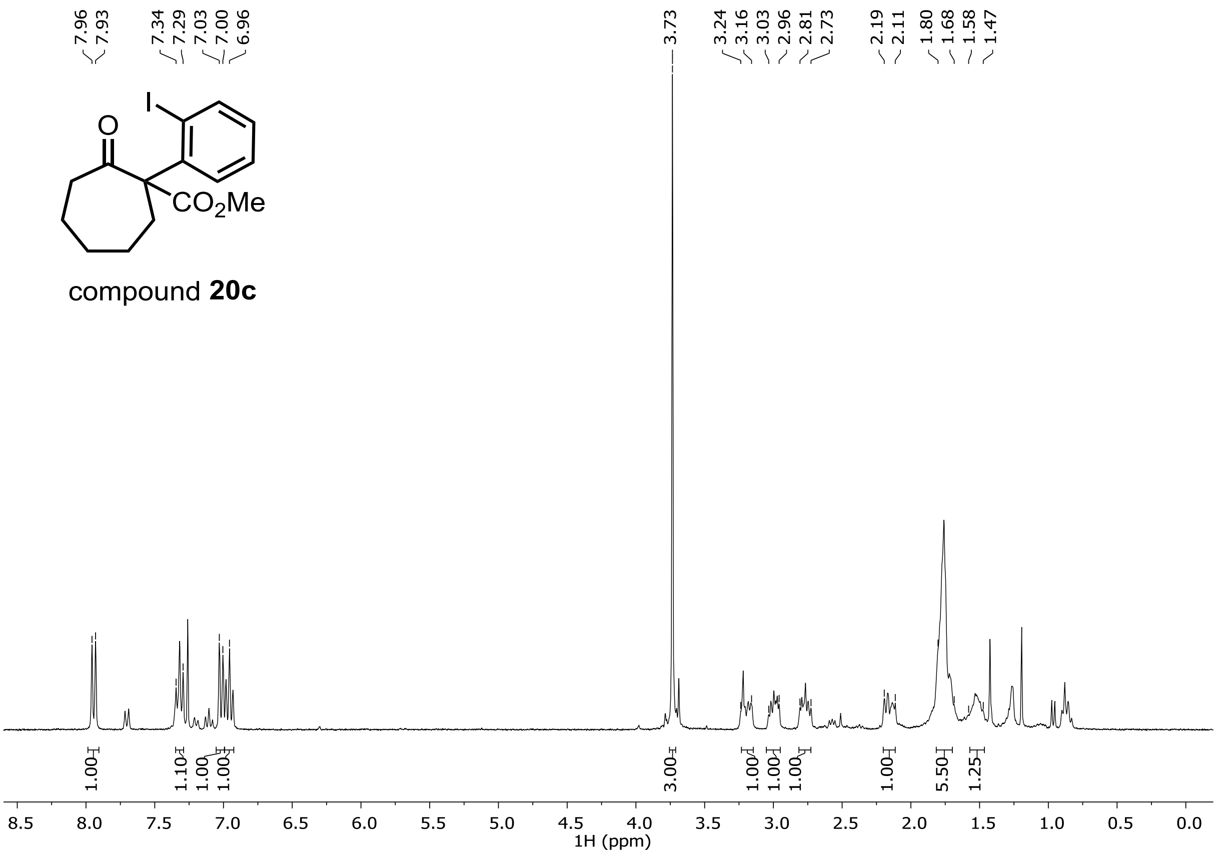
I
O
CO2Me
compound 20c
7.96
7.93
7.34
7.29
7.03
7.00
6.96
3.73
3.24
3.16
3.03
2.96
2.81
2.73
2.19
2.11
1.80
1.68
1.58
1.47
1.00
1.10
1.00
1.00
3.00
1.00
1.00
1.00
1.00
5.50
1.25
8.5
8.0
7.5
7.0
6.5
6.0
5.5
5.0
4.5
4.0
3.5
3.0
2.5
2.0
1.5
1.0
0.5
0.0
1H (ppm)

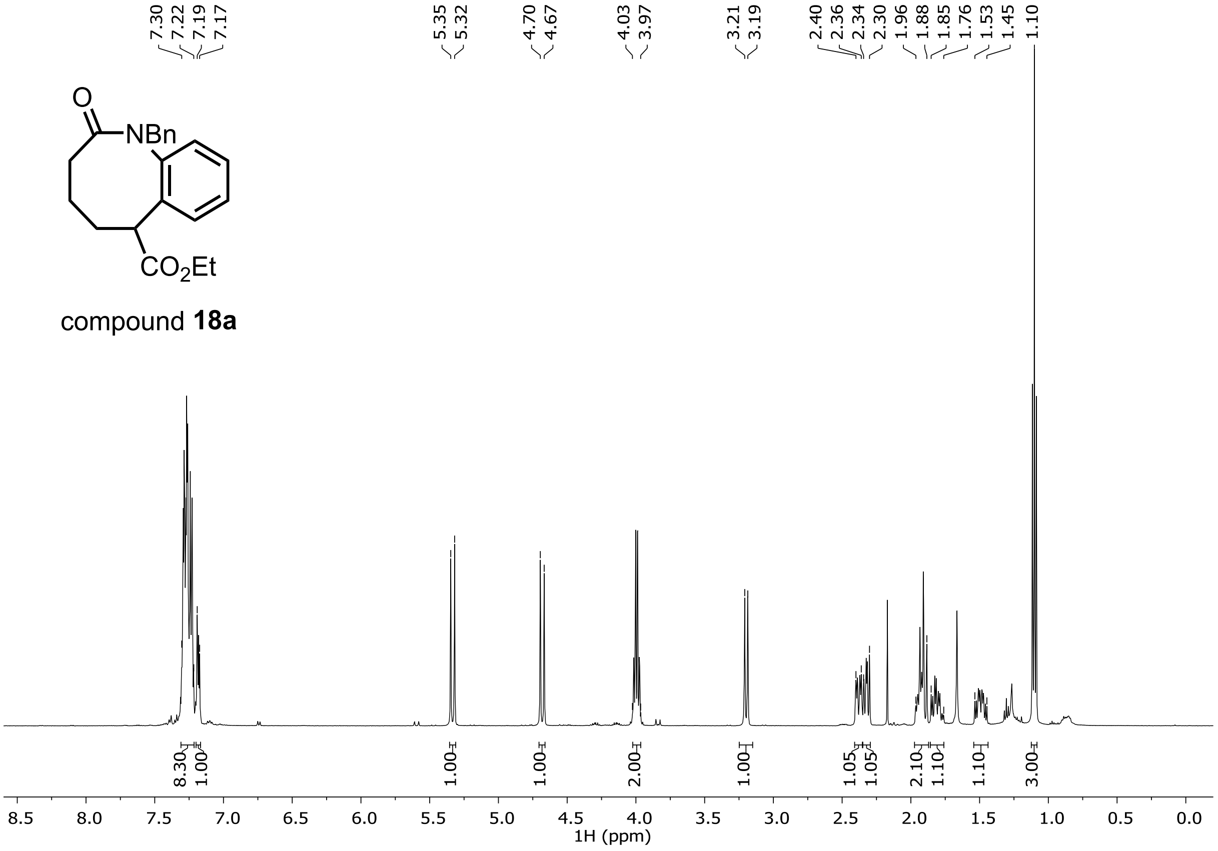
7.30
7.22
7.19
7.17
5.35
5.32
4.70
4.67
4.03
3.97
3.21
3.19
2.40
2.36
2.34
2.30
1.96
1.88
1.85
1.76
1.53
1.45
1.10
O
NBn
CO2Et
compound 18a
8.30
1.00
1.00
1.00
2.00
1.00
1.05
1.05
2.10
1.10
1.10
3.00
8.5 8.0 7.5 7.0 6.5 6.0 5.5 5.0 4.5 4.0 3.5 3.0 2.5 2.0 1.5 1.0 0.5 0.0
1H (ppm)

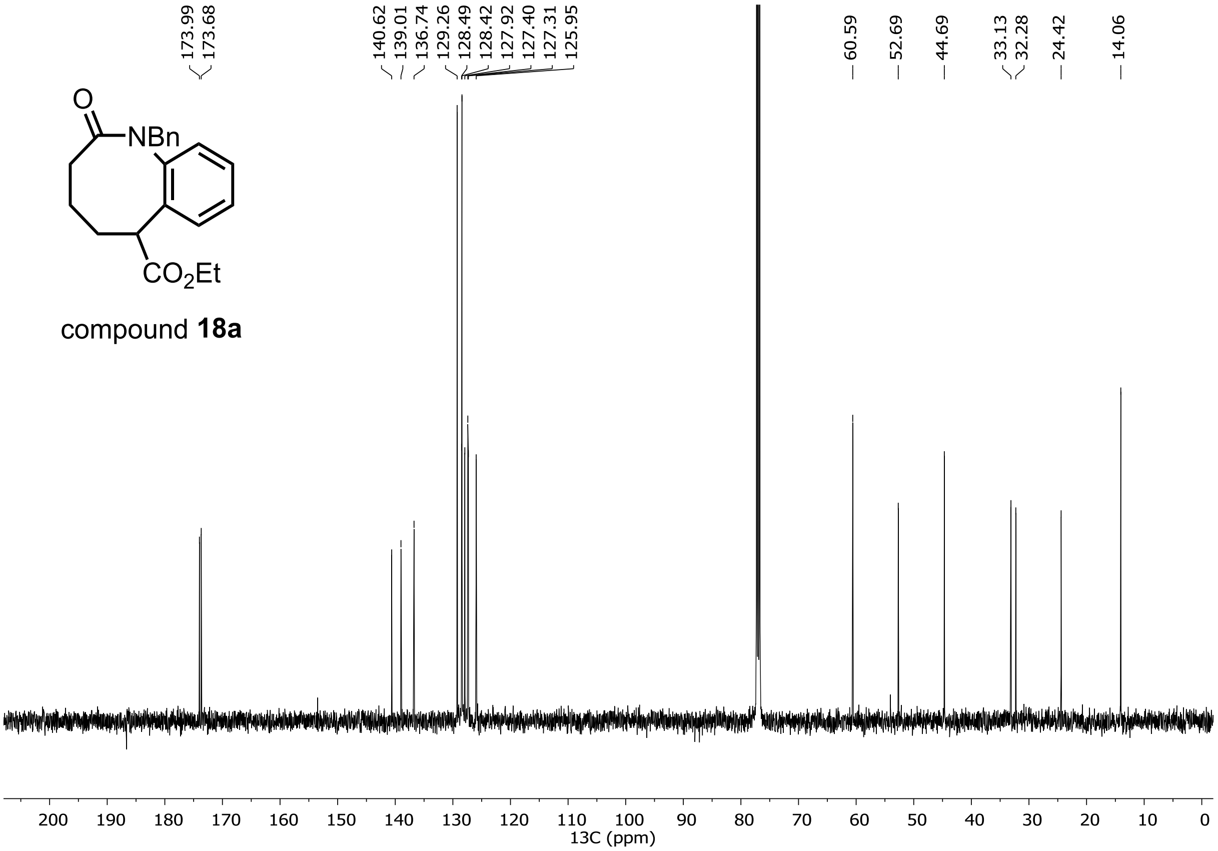
173.99
173.68
140.62
139.01
136.74
129.26
128.49
128.42
127.92
127.40
127.31
125.95
60.59
52.69
44.69
33.13
32.28
24.42
14.06
O
NBn
CO2Et
compound 18a
200 190 180 170 160 150 140 130 120 110 100 90 80 70 60 50 40 30 20 10 0
13C (ppm)

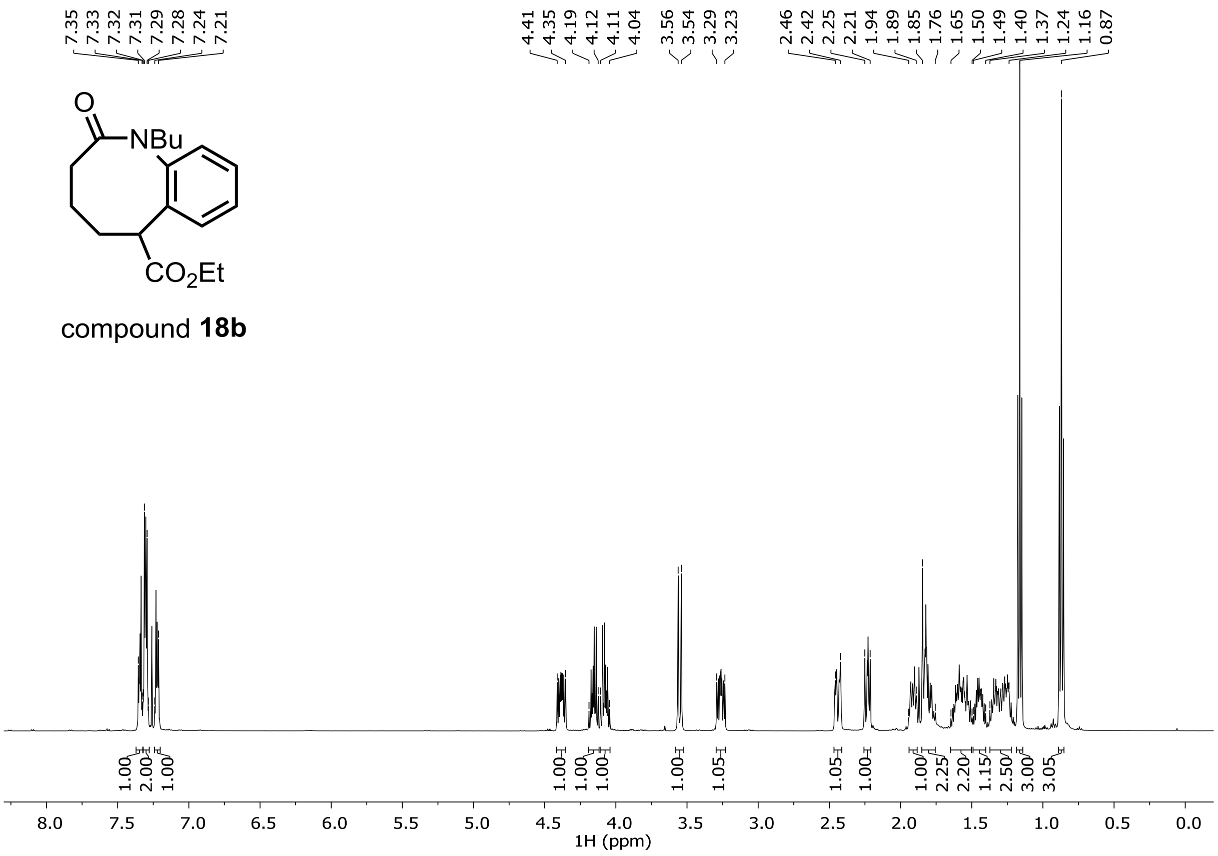

compound **18b**

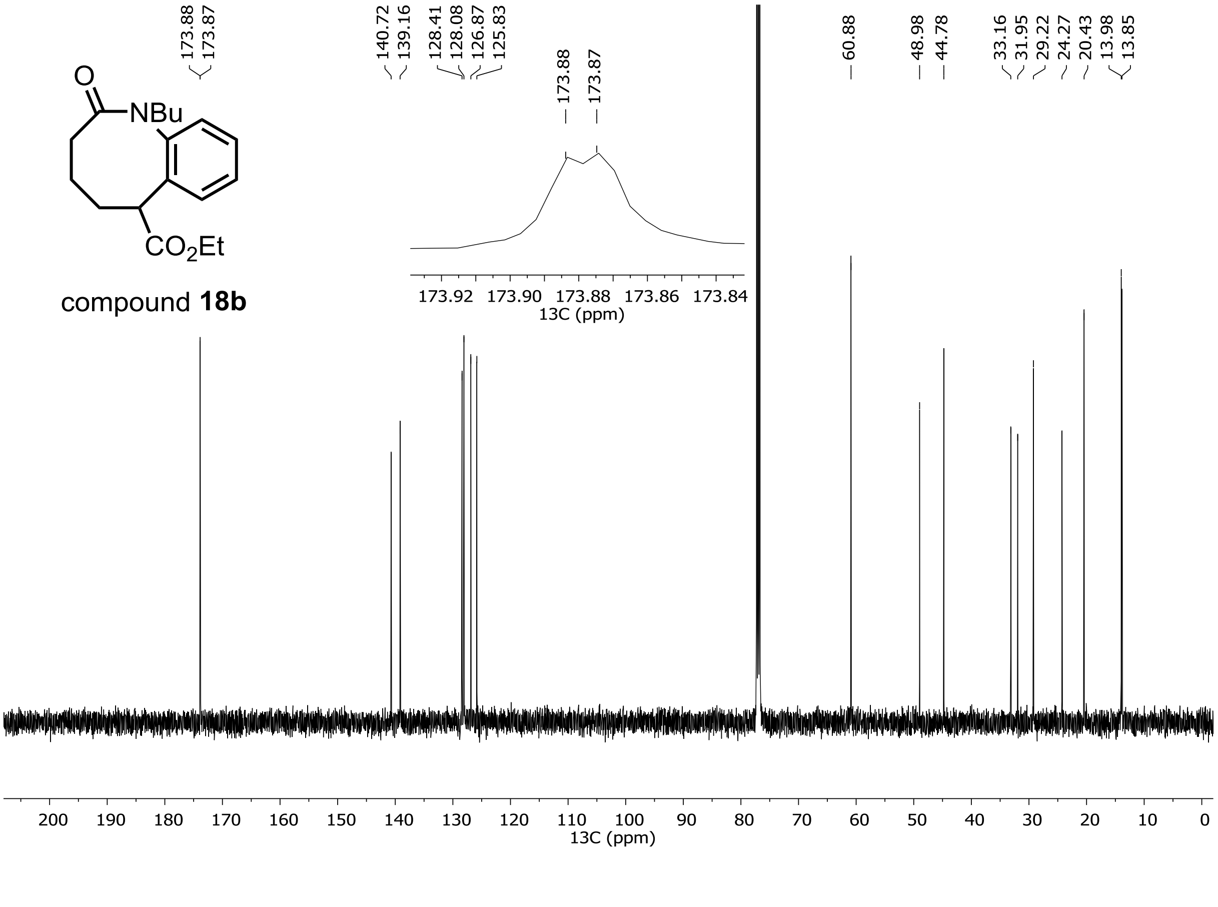

compound **18b**

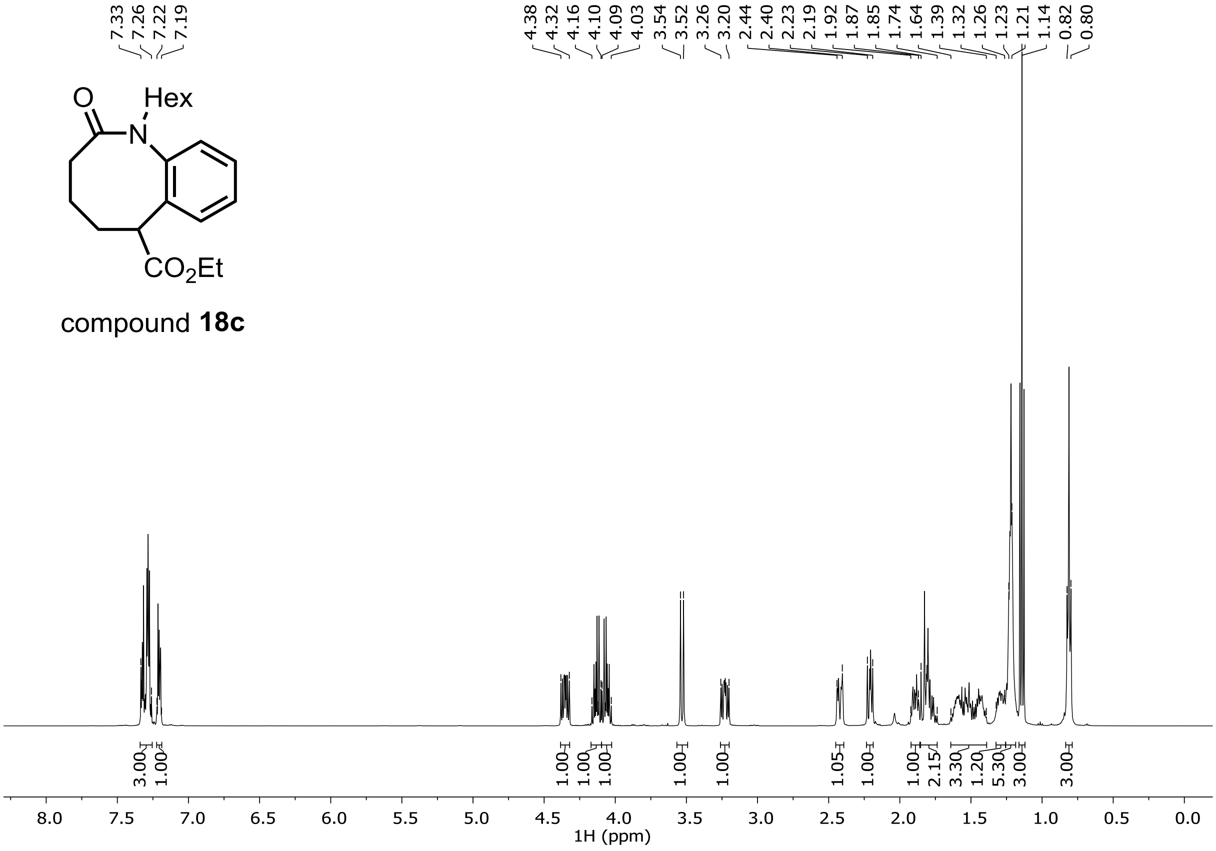

compound **18c**

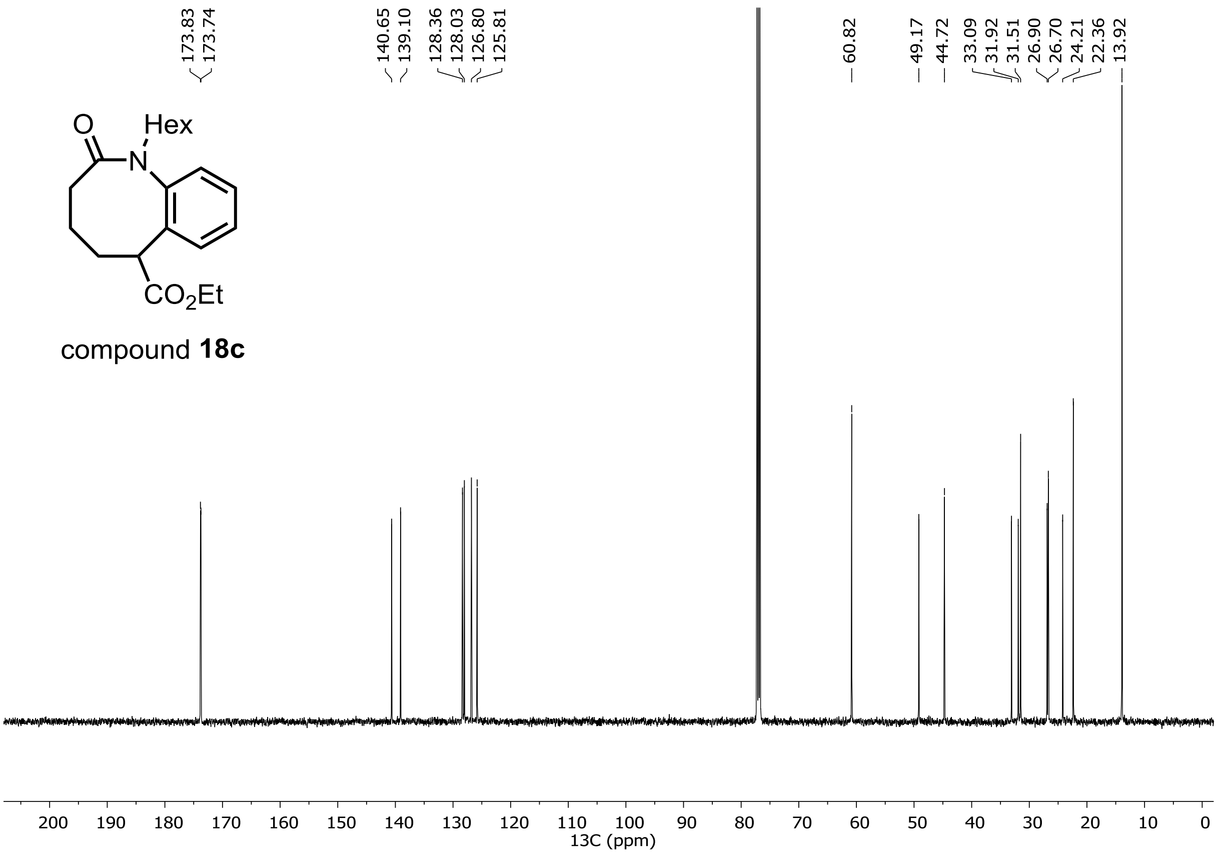

compound **18c**

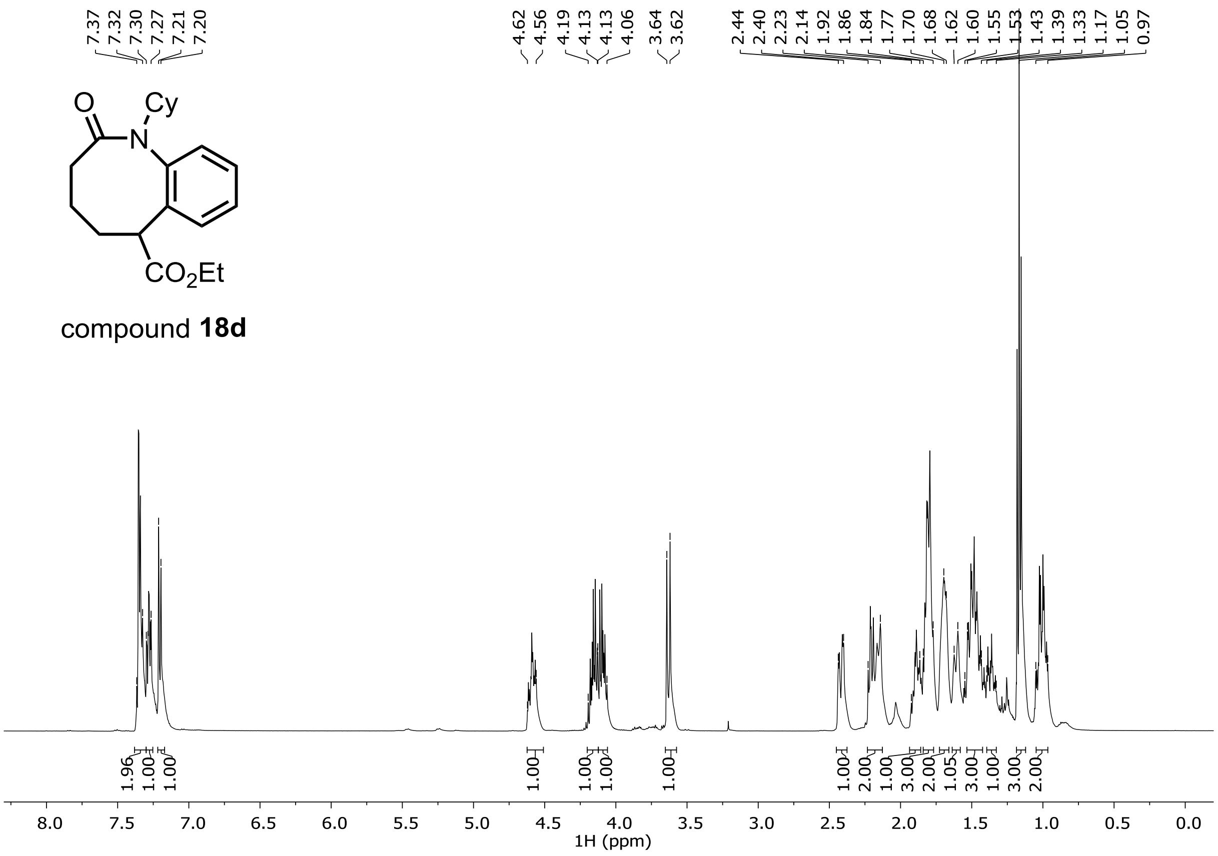

compound **18d**

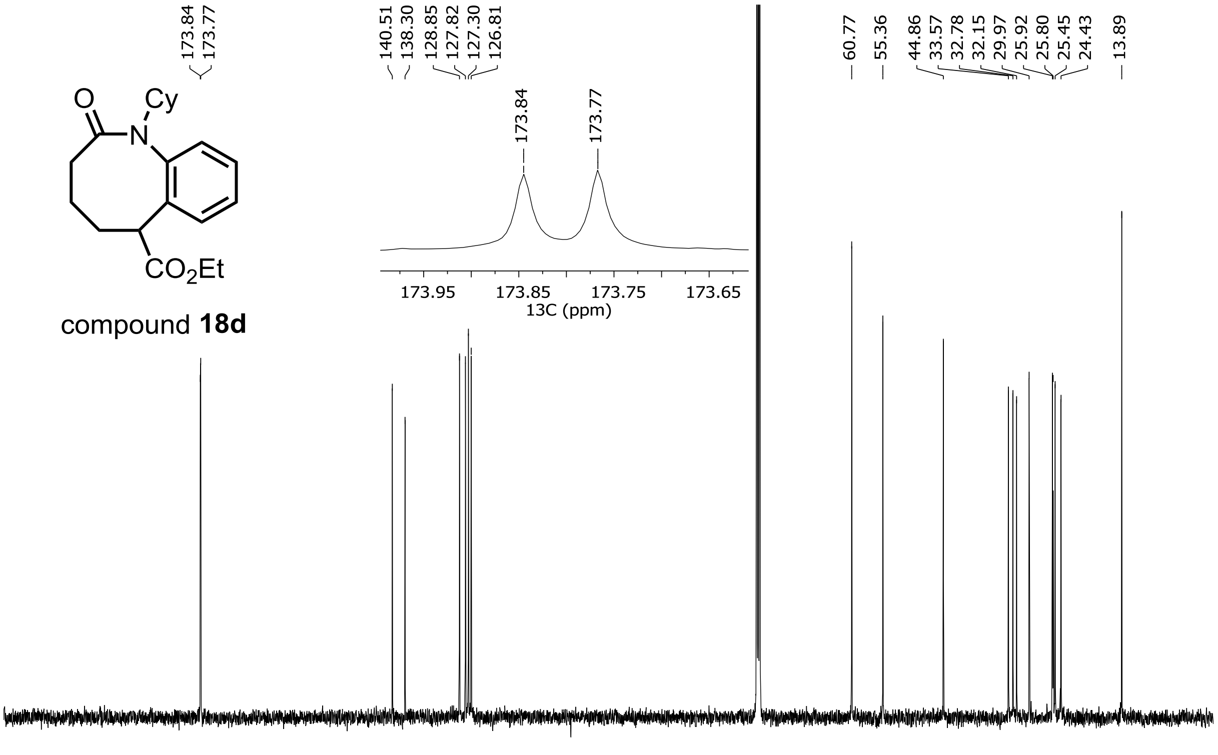

compound **18d**

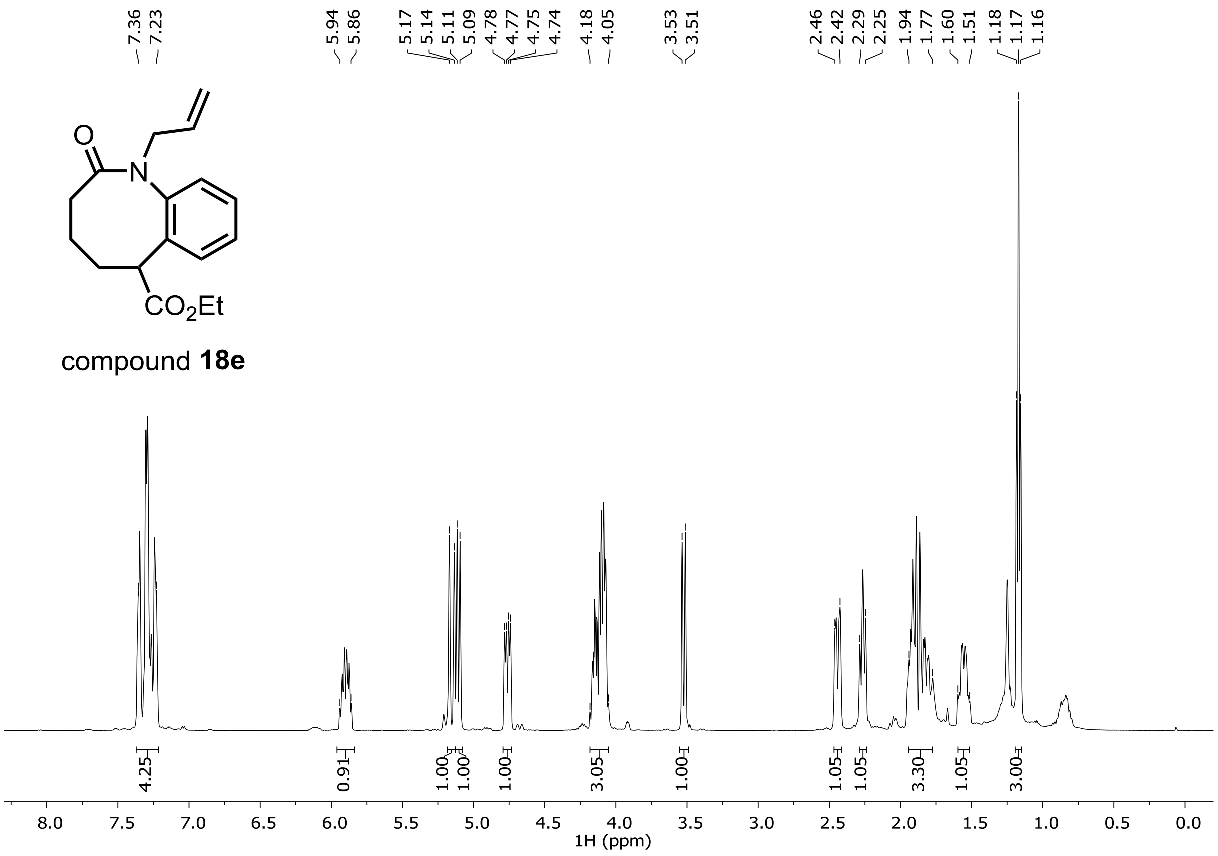
compound 18e
1H (ppm)
13C (ppm)

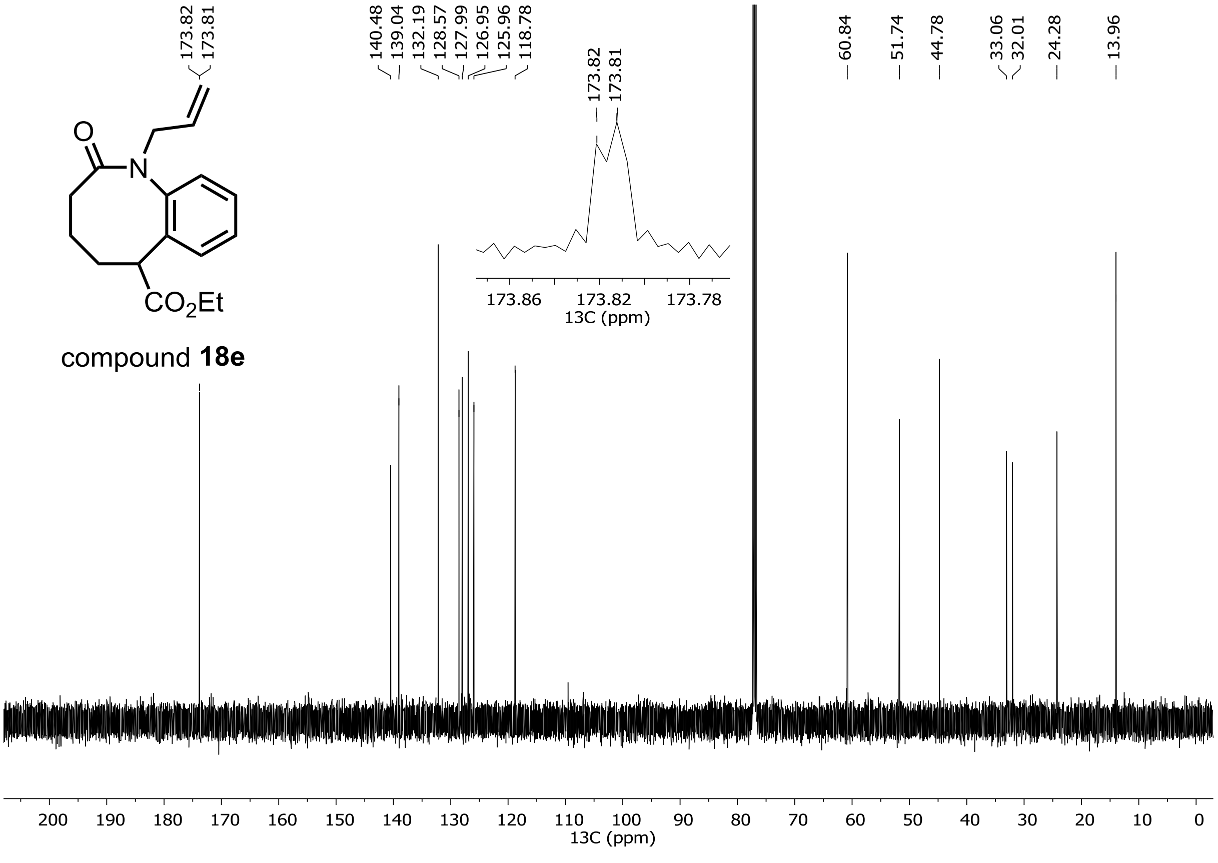
compound 18e
13C (ppm)

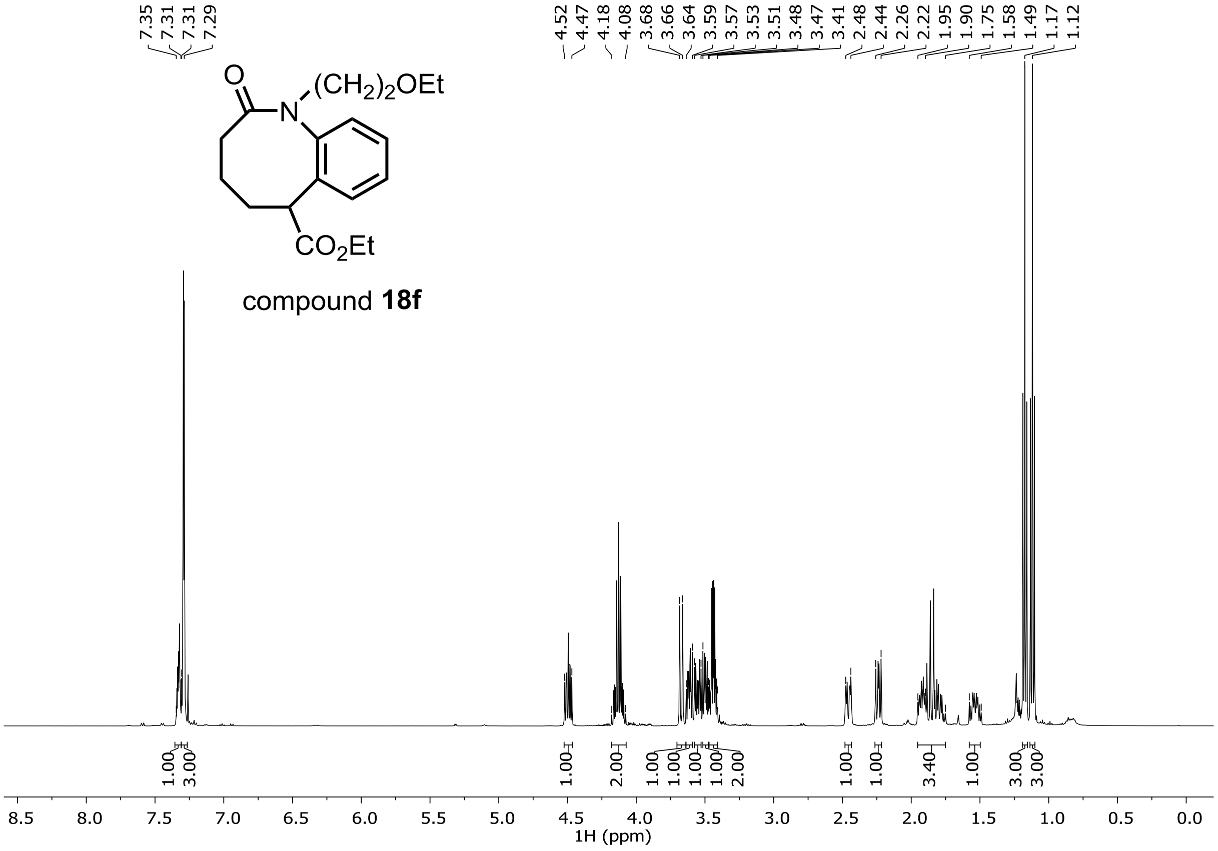
7.35
7.31
7.31
7.29
4.52
4.47
4.18
4.08
3.68
3.66
3.64
3.59
3.57
3.53
3.51
3.48
3.47
3.41
2.48
2.44
2.26
2.22
1.95
1.90
1.75
1.58
1.49
1.17
1.12
(CH2)2OEt
CO2Et
compound 18f
1.00
3.00
1.00
2.00
1.00
1.00
1.00
1.00
2.00
1.00
1.00
3.40
1.00
3.00
3.00
1H (ppm)

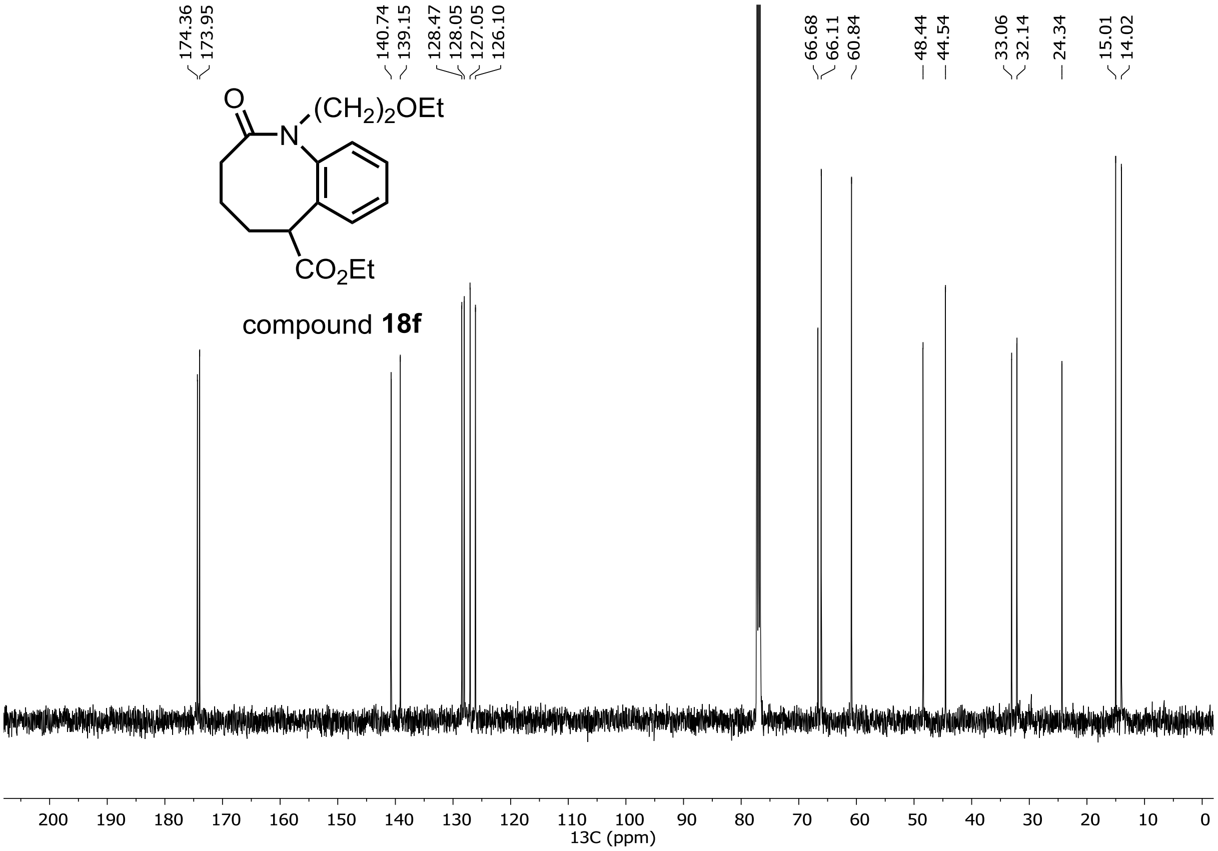
174.36
173.95
140.74
139.15
128.47
128.05
127.05
126.10
66.68
66.11
60.84
48.44
44.54
33.06
32.14
24.34
15.01
14.02
(CH2)2OEt
CO2Et
compound 18f
13C (ppm)

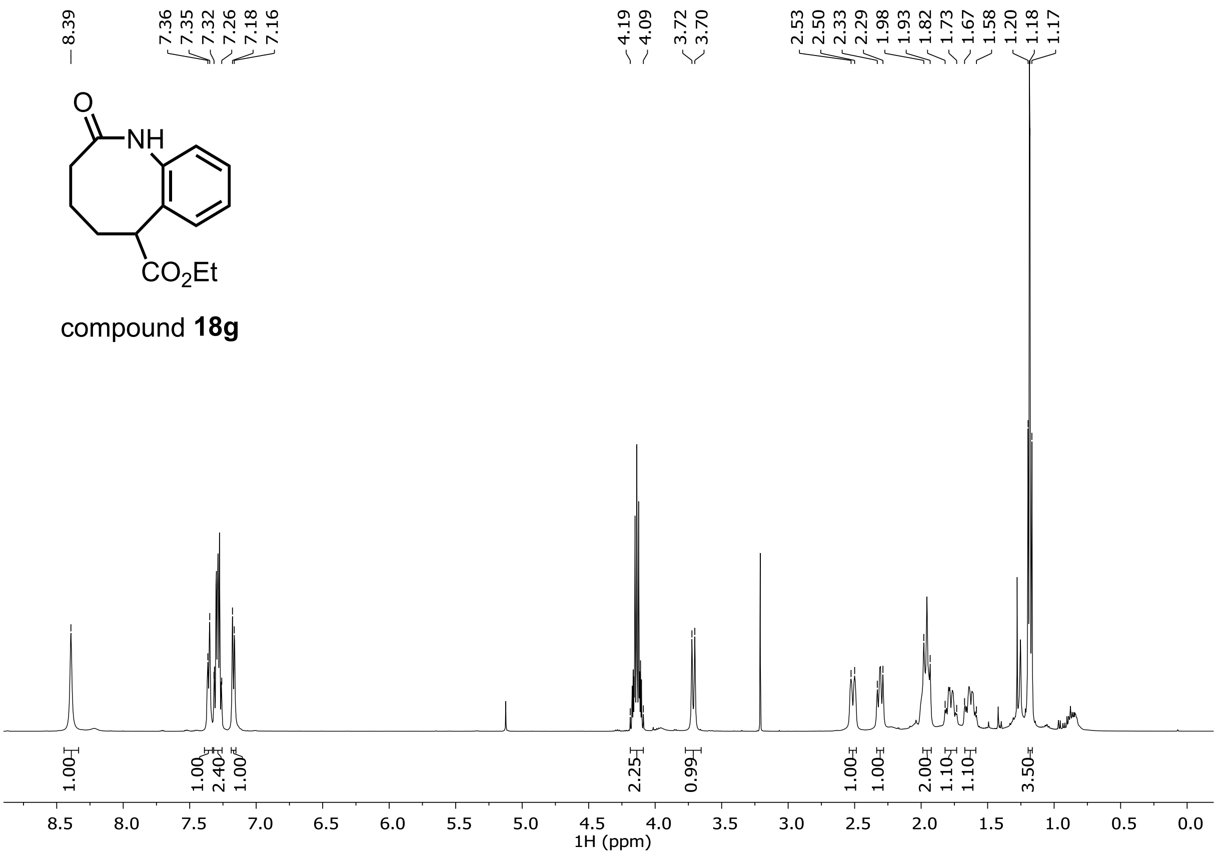

compound **18g**

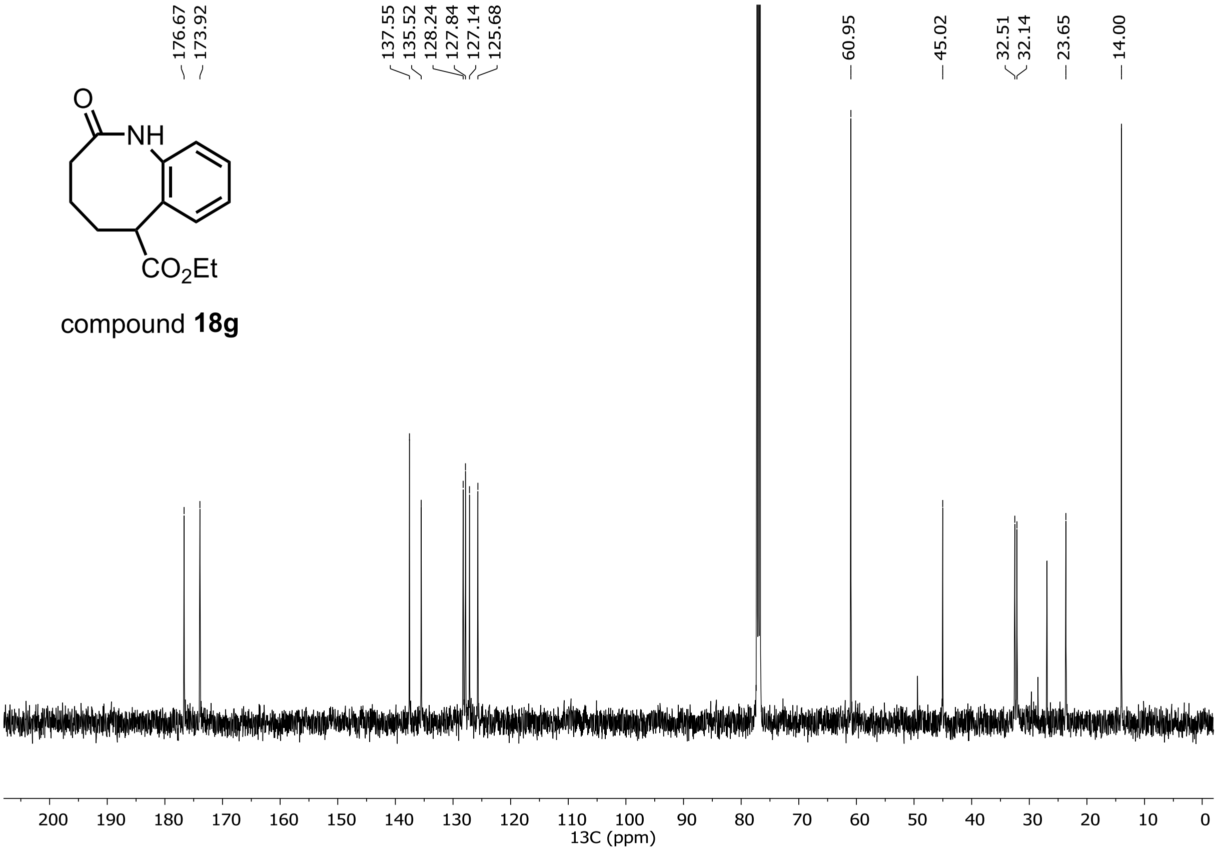

compound **18g**

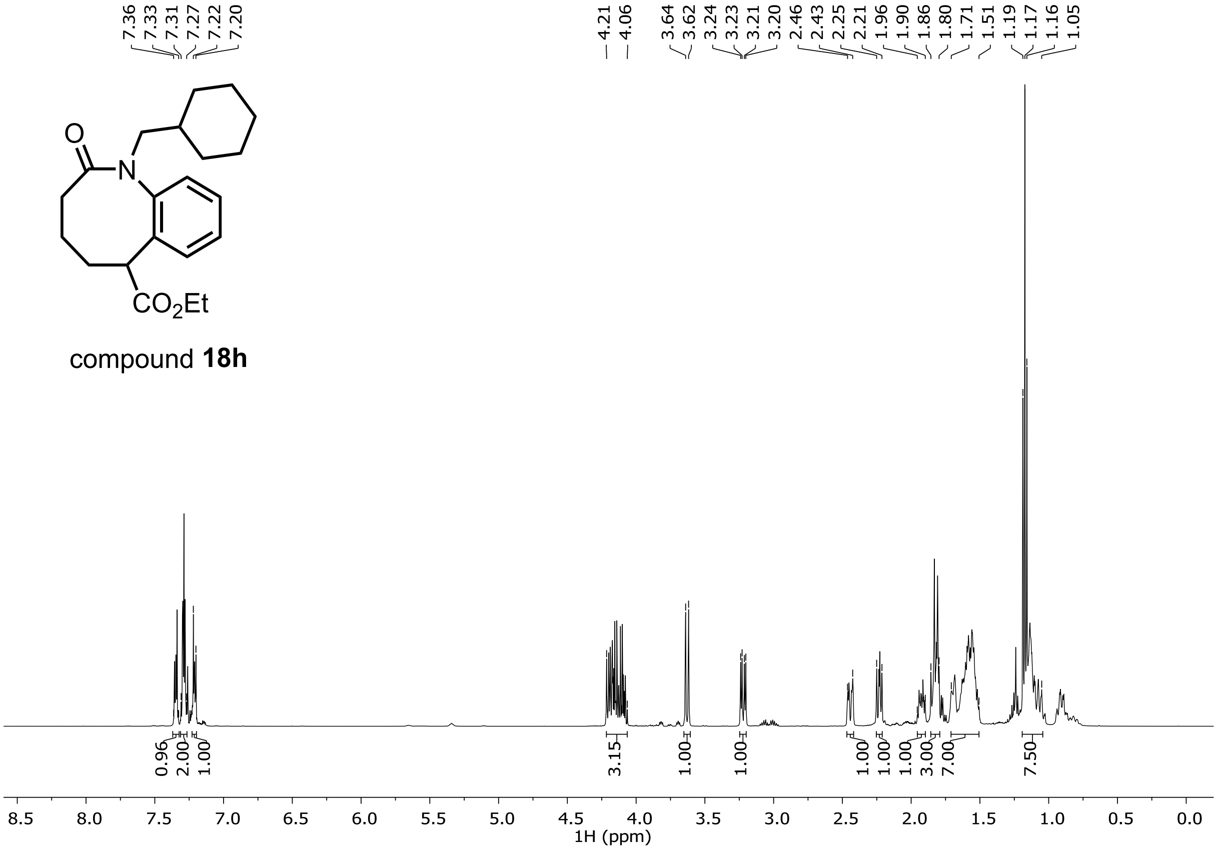
7.36
7.33
7.31
7.27
7.22
7.20
4.21
4.06
3.64
3.62
3.24
3.23
3.21
3.20
2.46
2.43
2.25
2.21
1.96
1.90
1.86
1.80
1.71
1.51
1.19
1.17
1.16
1.05
O
N
CO_2Et
compound **18h**
0.96
2.00
1.00
3.15
1.00
1.00
1.00
1.00
1.00
3.00
7.00
7.50
8.5 8.0 7.5 7.0 6.5 6.0 5.5 5.0 4.5 4.0 3.5 3.0 2.5 2.0 1.5 1.0 0.5 0.0
1H (ppm)

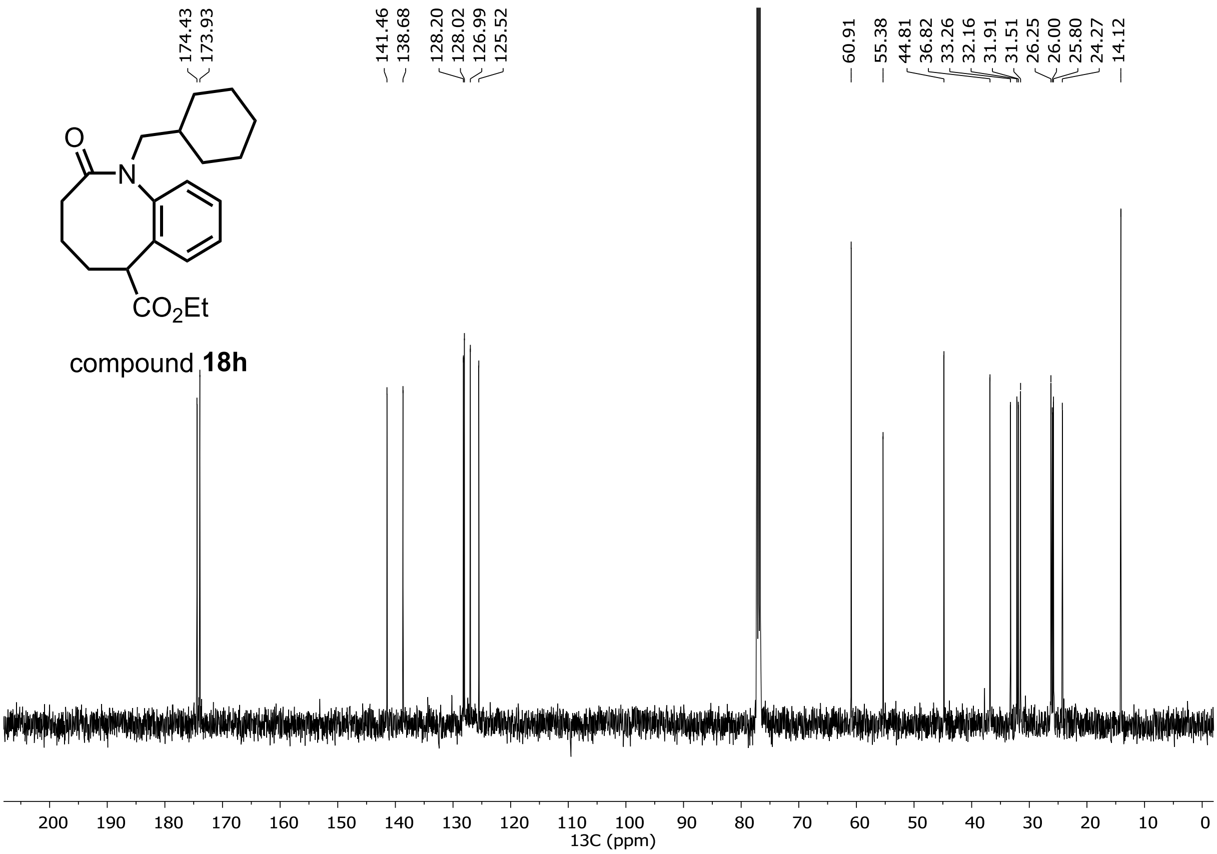
174.43
173.93
141.46
138.68
128.20
128.02
126.99
125.52
60.91
55.38
44.81
36.82
33.26
32.16
31.91
31.51
26.25
26.00
25.80
24.27
14.12
O
N
CO_2Et
compound **18h**
200 190 180 170 160 150 140 130 120 110 100 90 80 70 60 50 40 30 20 10 0
13C (ppm)

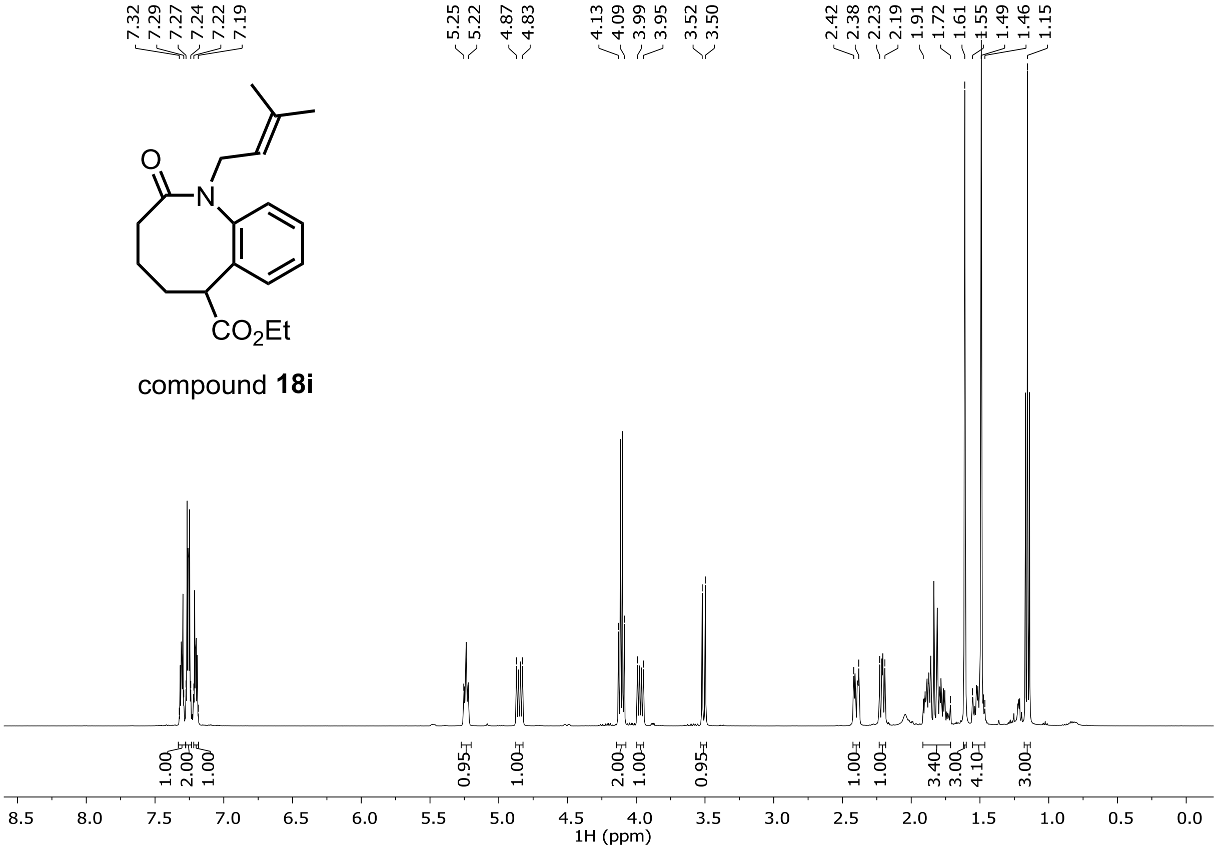
7.32 7.29 7.27 7.24 7.22 7.19
5.25 5.22 4.87 4.83
4.13 4.09 3.99 3.95 3.52 3.50
2.42 2.38 2.23 2.19 1.91 1.72 1.61 1.55 1.49 1.46 1.15
O
N
CO2Et
compound 18i
1.00 2.00 1.00 0.95 1.00 2.00 1.00 0.95 1.00 1.00 3.40 3.00 4.10 3.00
8.5 8.0 7.5 7.0 6.5 6.0 5.5 5.0 4.5 4.0 3.5 3.0 2.5 2.0 1.5 1.0 0.5 0.0
1H (ppm)

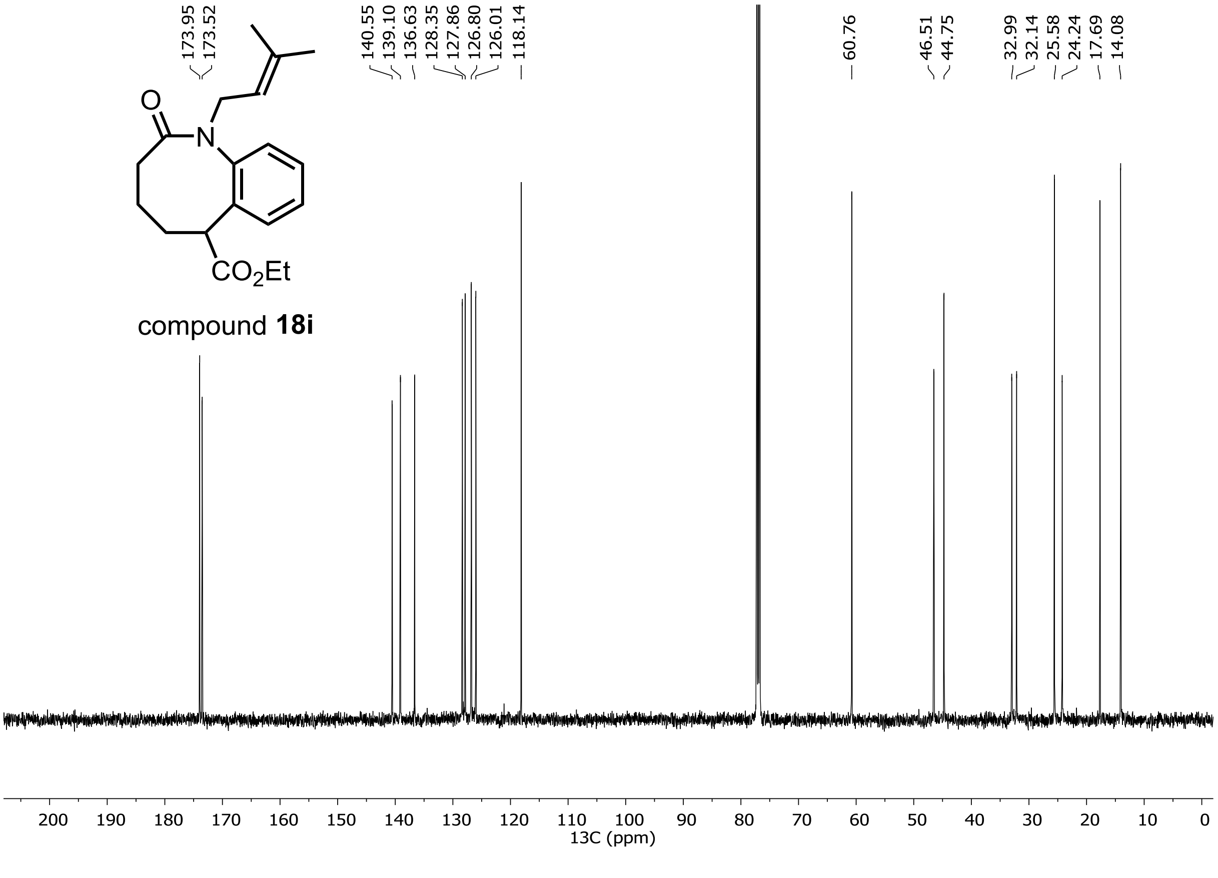
173.95 173.52
140.55 139.10 136.63 128.35 127.86 126.80 126.01 118.14
60.76
46.51 44.75
32.99 32.14 25.58 24.24 17.69 14.08
O
N
CO2Et
compound 18i
200 190 180 170 160 150 140 130 120 110 100 90 80 70 60 50 40 30 20 10 0
13C (ppm)

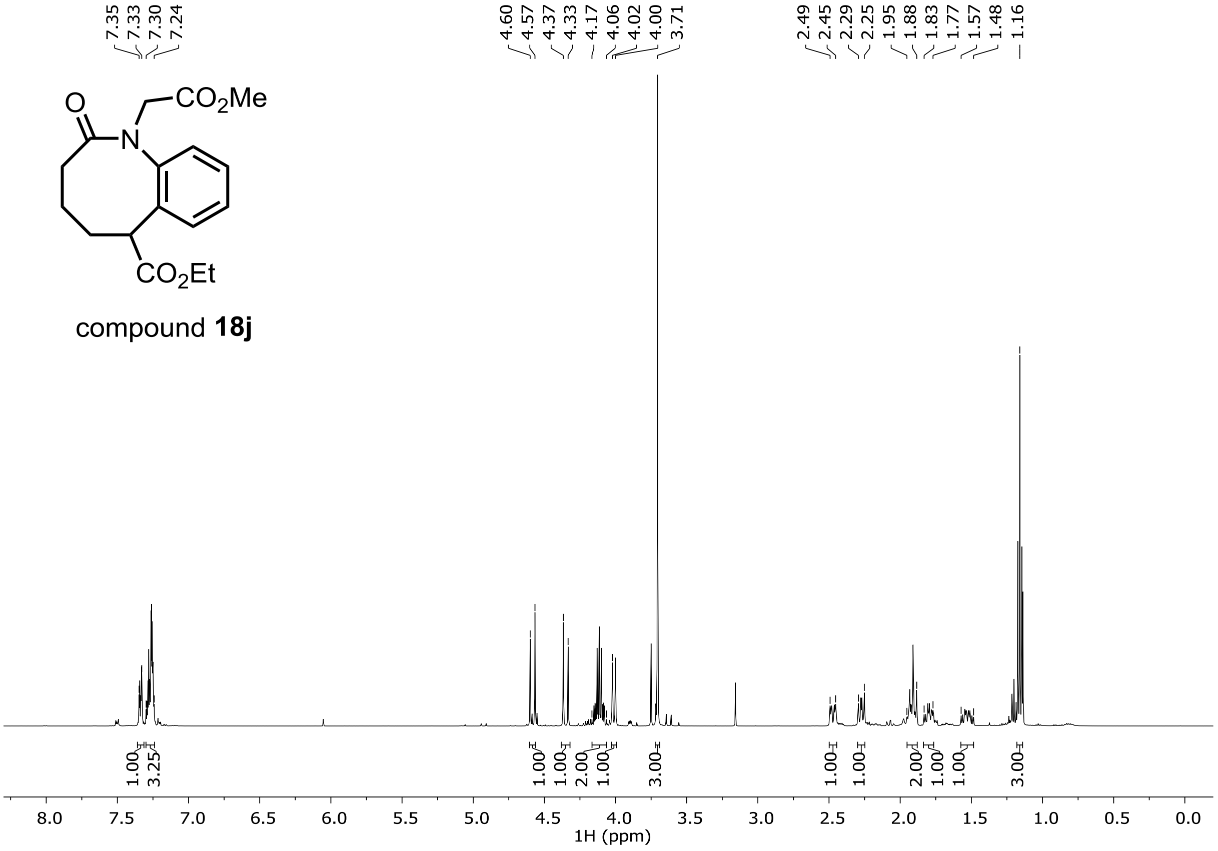
7.35
7.33
7.30
7.24
4.60
4.57
4.37
4.33
4.17
4.06
4.02
4.00
3.71
2.49
2.45
2.29
2.25
1.95
1.88
1.83
1.77
1.57
1.48
1.16
CO2Me
O
N
CO2Et
compound 18j
1.00
3.25
1.00
1.00
2.00
1.00
3.00
1.00
1.00
2.00
1.00
1.00
3.00
8.0 7.5 7.0 6.5 6.0 5.5 5.0 4.5 4.0 3.5 3.0 2.5 2.0 1.5 1.0 0.5 0.0
1H (ppm)

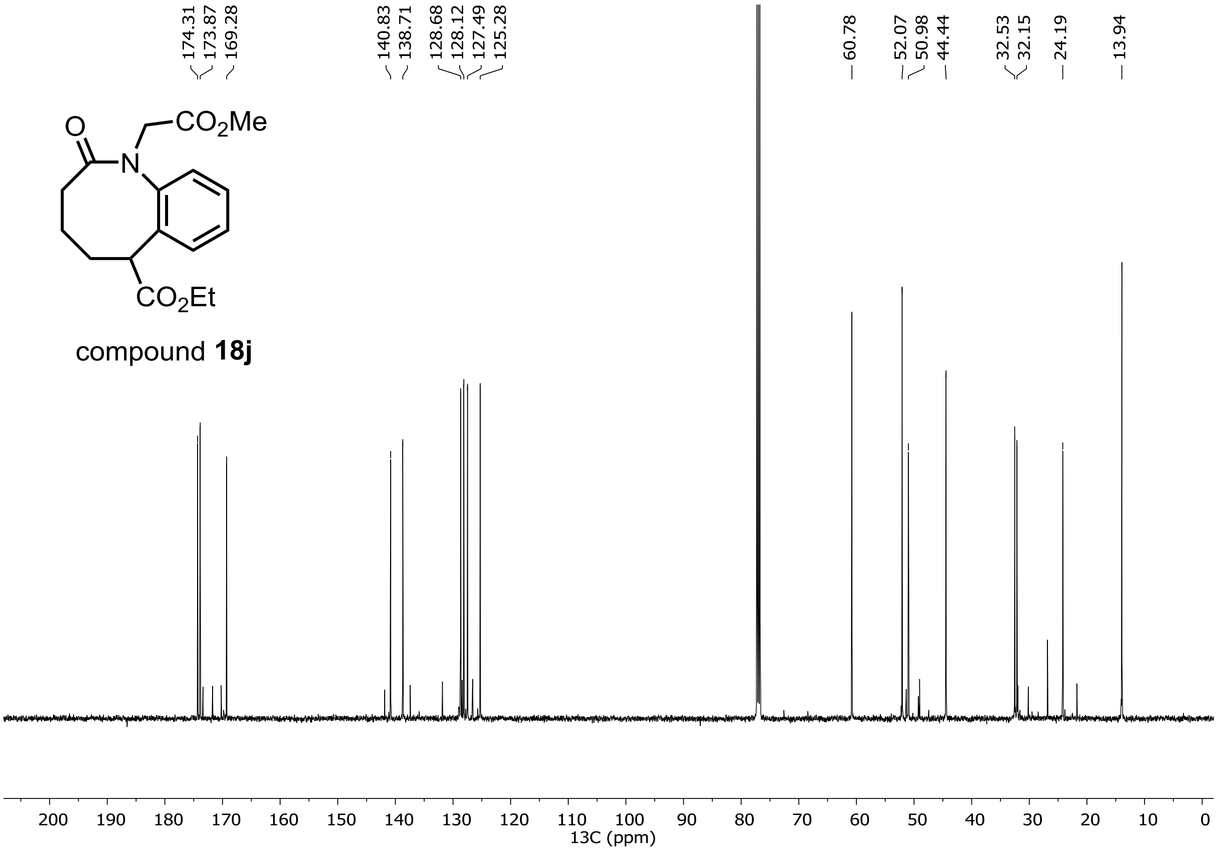
174.31
173.87
169.28
140.83
138.71
128.68
128.12
127.49
125.28
60.78
52.07
50.98
44.44
32.53
32.15
24.19
13.94
CO2Me
O
N
CO2Et
compound 18j
200 190 180 170 160 150 140 130 120 110 100 90 80 70 60 50 40 30 20 10 0
13C (ppm)

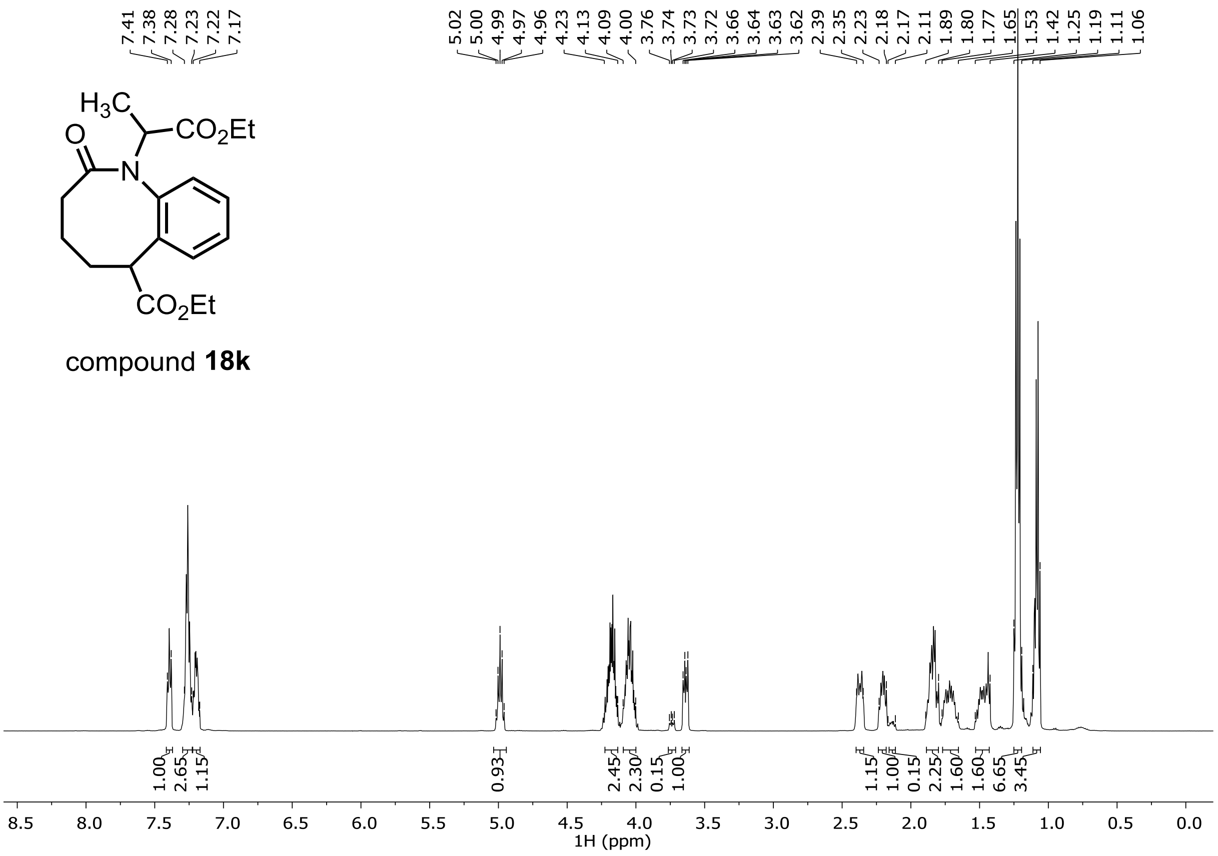
7.41 7.38 7.28 7.23 7.22 7.17
5.02 5.00 4.99 4.97 4.96 4.23 4.13 4.09 4.00 3.76 3.74 3.73 3.72 3.66 3.64 3.63 3.62 2.39 2.35 2.23 2.18 2.17 2.11 1.89 1.80 1.77 1.65 1.53 1.42 1.25 1.19 1.11 1.06
H_3C
CO_2Et
O
N
CO_2Et
compound **18k**
1.00 2.65 1.15 0.93 2.45 2.30 0.15 1.00 1.15 1.00 0.15 2.25 1.60 1.60 6.65 3.45
8.5 8.0 7.5 7.0 6.5 6.0 5.5 5.0 4.5 4.0 3.5 3.0 2.5 2.0 1.5 1.0 0.5 0.0
1H (ppm)

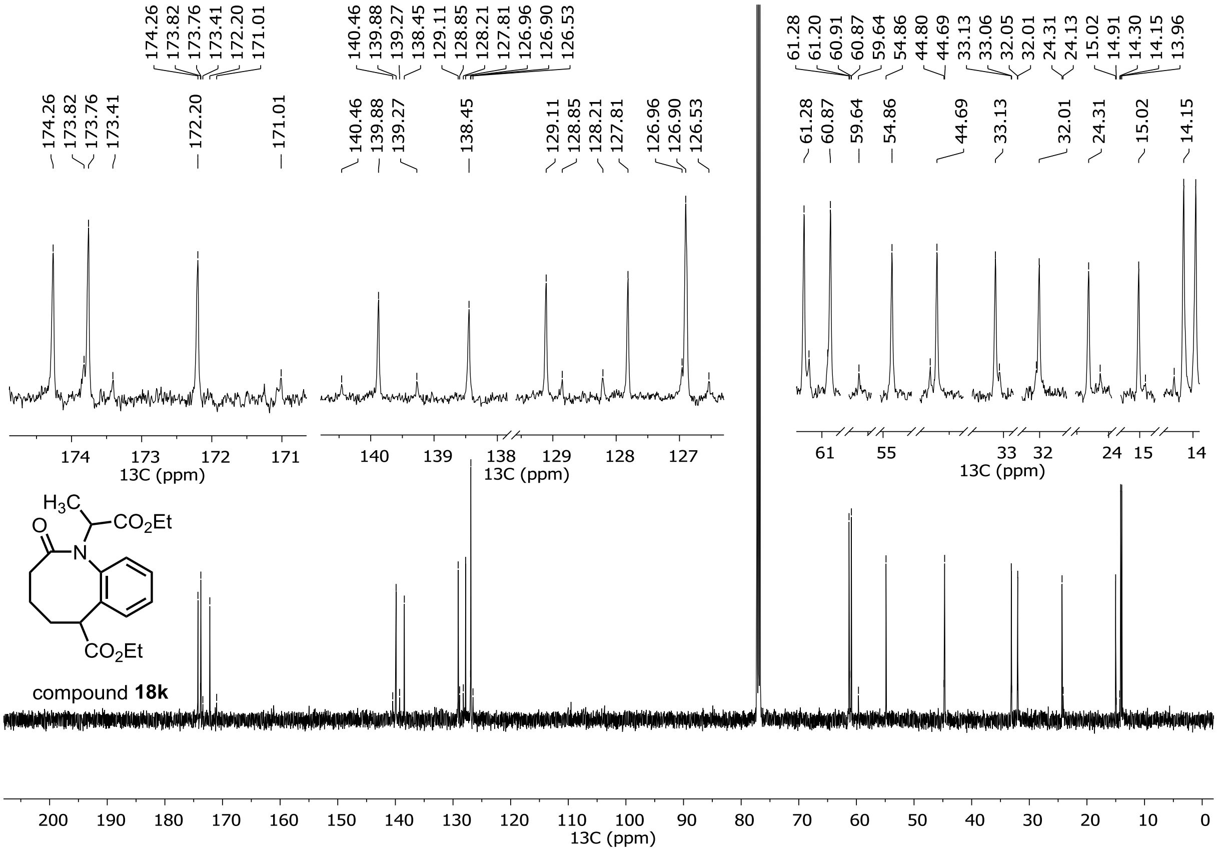
174.26 173.82 173.76 173.41 172.20 171.01
140.46 139.88 139.27 138.45 129.11 128.85 128.21 127.81 126.96 126.90 126.53
61.28 61.20 60.91 60.87 59.64 54.86 44.80 44.69 33.13 33.06 32.05 32.01 24.31 24.13 15.02 14.91 14.30 14.15 13.96
174 173 172 171
13C (ppm)
140 139 138 129 128 127
13C (ppm)
61 55 33 32 24 15 14
13C (ppm)
H_3C
CO_2Et
O
N
CO_2Et
compound **18k**
200 190 180 170 160 150 140 130 120 110 100 90 80 70 60 50 40 30 20 10 0
13C (ppm)

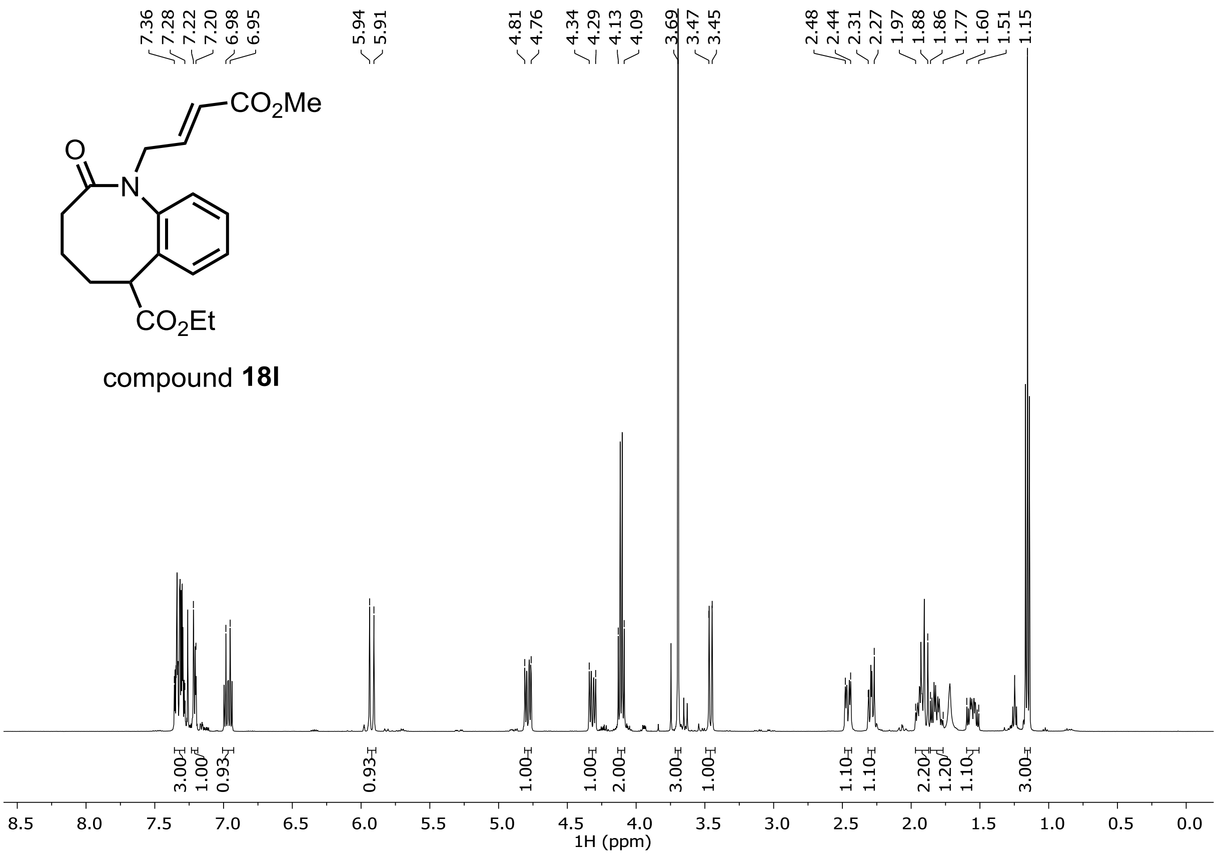
CO2Me
O
N
CO2Et
compound 18l
7.36 7.28 7.22 7.20 6.98 6.95 5.94 5.91 4.81 4.76 4.34 4.29 4.13 4.09 3.69 3.47 3.45 2.48 2.44 2.31 2.27 1.97 1.88 1.86 1.77 1.60 1.51 1.15
3.00 1.00 0.93 0.93 1.00 1.00 2.00 3.00 1.00 1.10 1.10 2.20 1.20 1.10 3.00
1H (ppm)

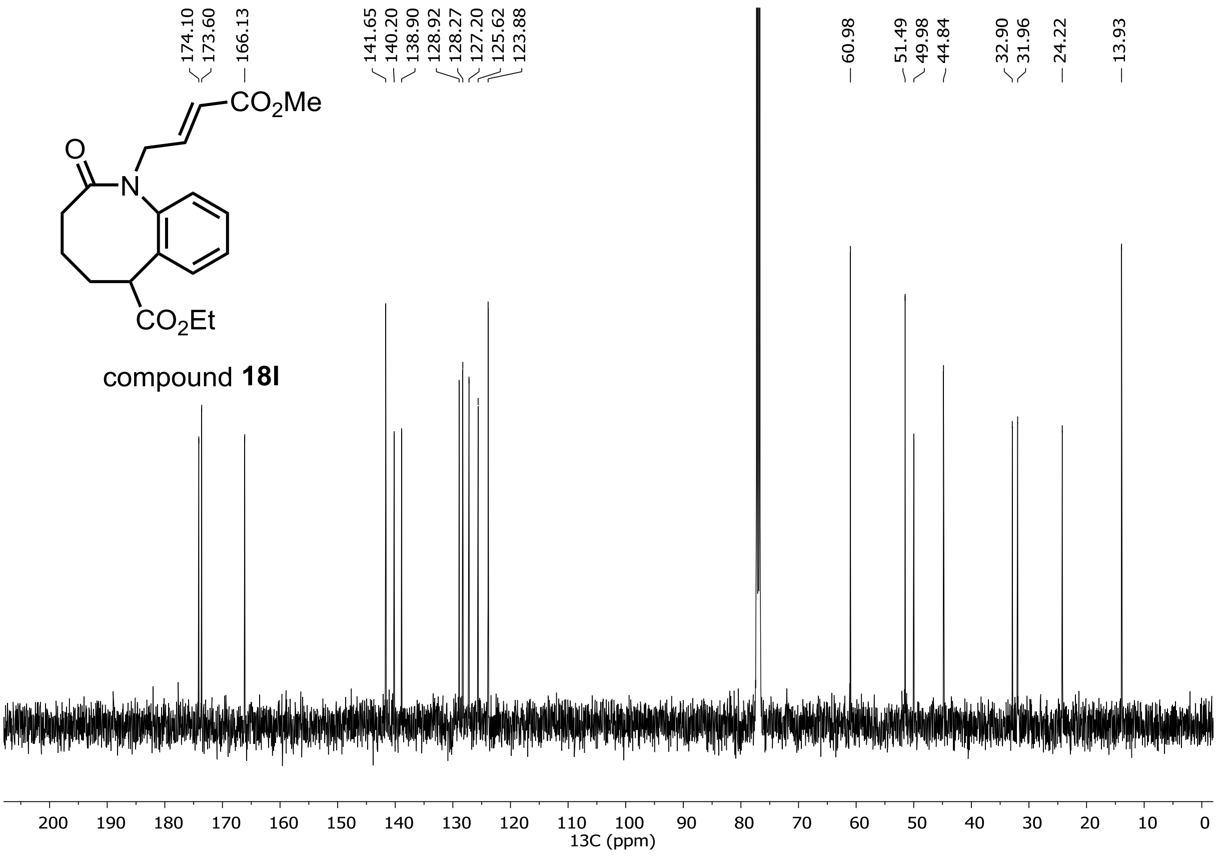
CO2Me
O
N
CO2Et
compound 18l
174.10 173.60 166.13 141.65 140.20 138.90 128.92 128.27 127.20 125.62 123.88 60.98 51.49 49.98 44.84 32.90 31.96 24.22 13.93
13C (ppm)

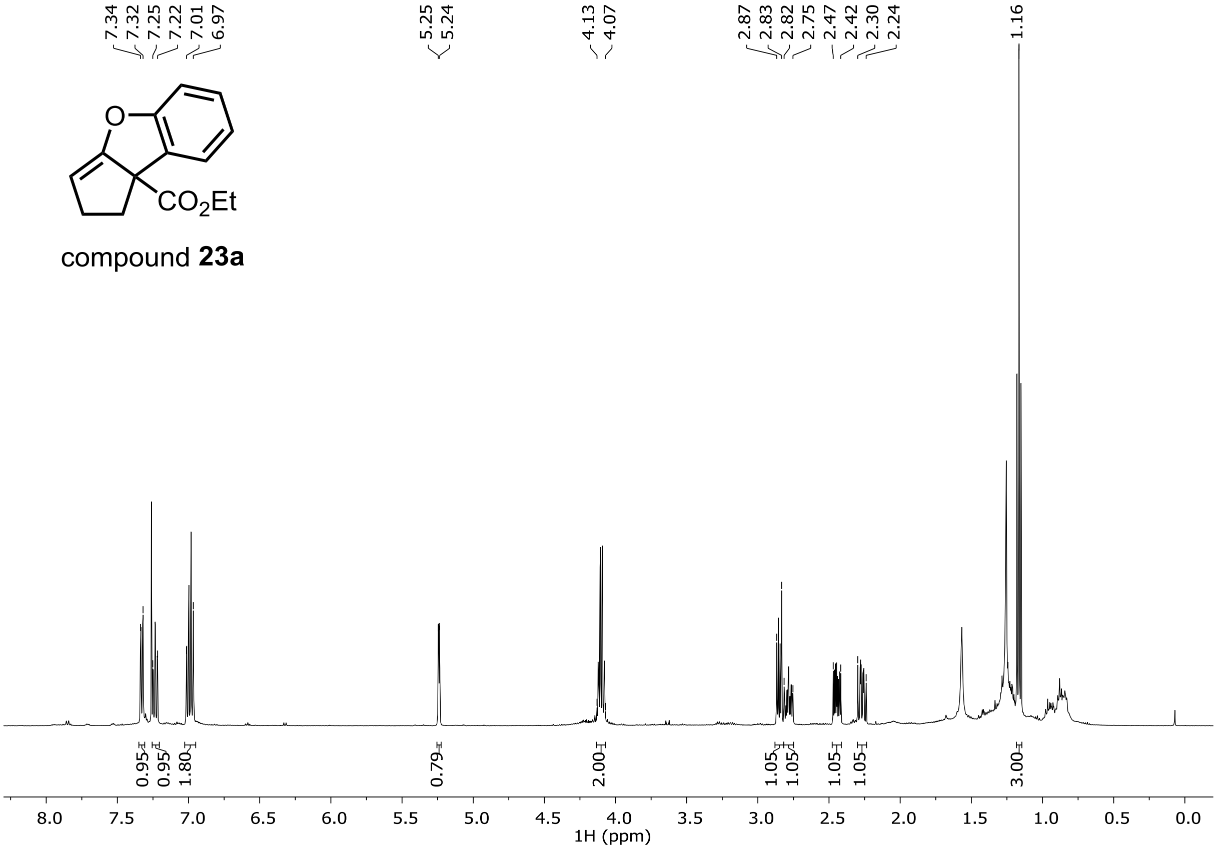

compound **23a**
CO_2Et
O
7.34
7.32
7.25
7.22
7.01
6.97
5.25
5.24
4.13
4.07
2.87
2.83
2.82
2.75
2.47
2.42
2.30
2.24
1.16
0.95
0.95
1.80
0.79
2.00
1.05
1.05
1.05
1.05
3.00
8.0 7.5 7.0 6.5 6.0 5.5 5.0 4.5 4.0 3.5 3.0 2.5 2.0 1.5 1.0 0.5 0.0
1H (ppm)

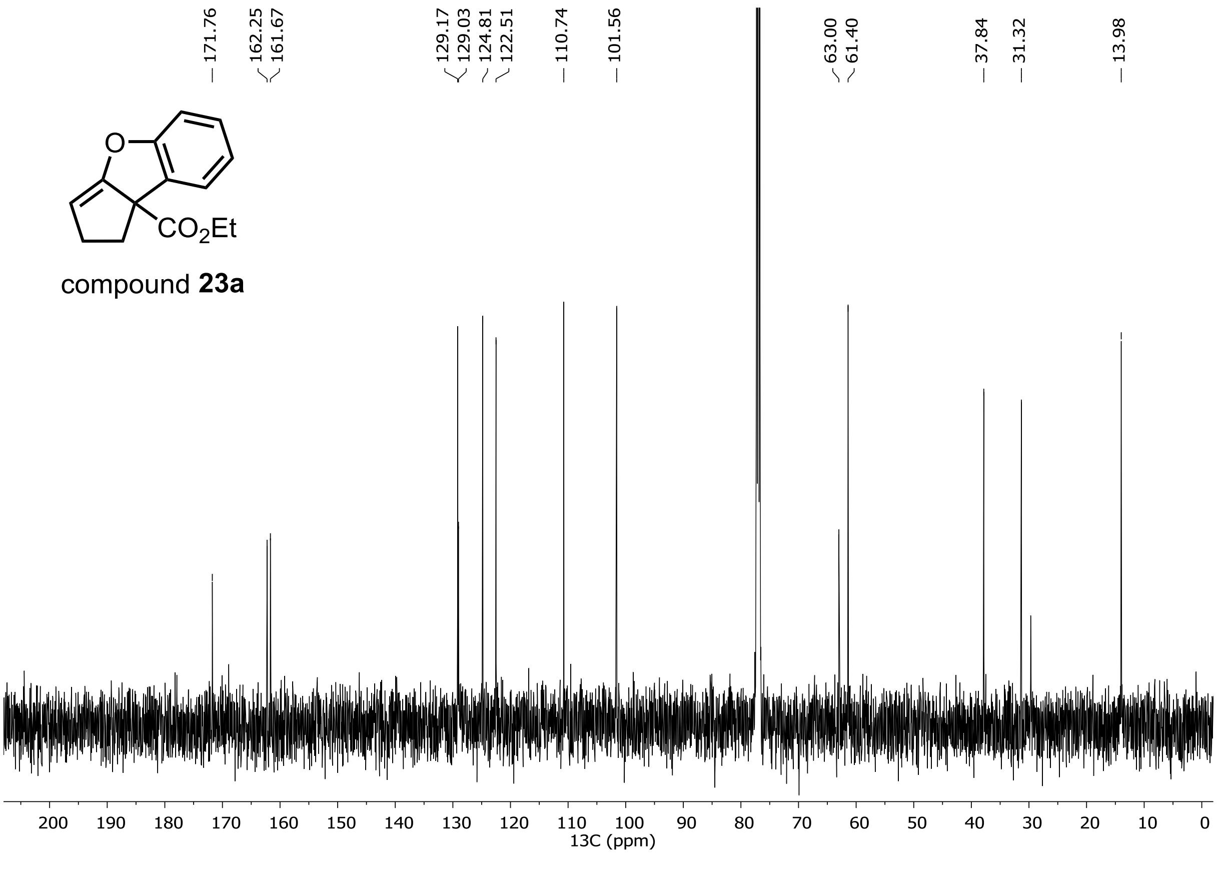

compound **23a**
CO_2Et
O
171.76
162.25
161.67
129.17
129.03
124.81
122.51
110.74
101.56
63.00
61.40
37.84
31.32
13.98
200 190 180 170 160 150 140 130 120 110 100 90 80 70 60 50 40 30 20 10 0
13C (ppm)

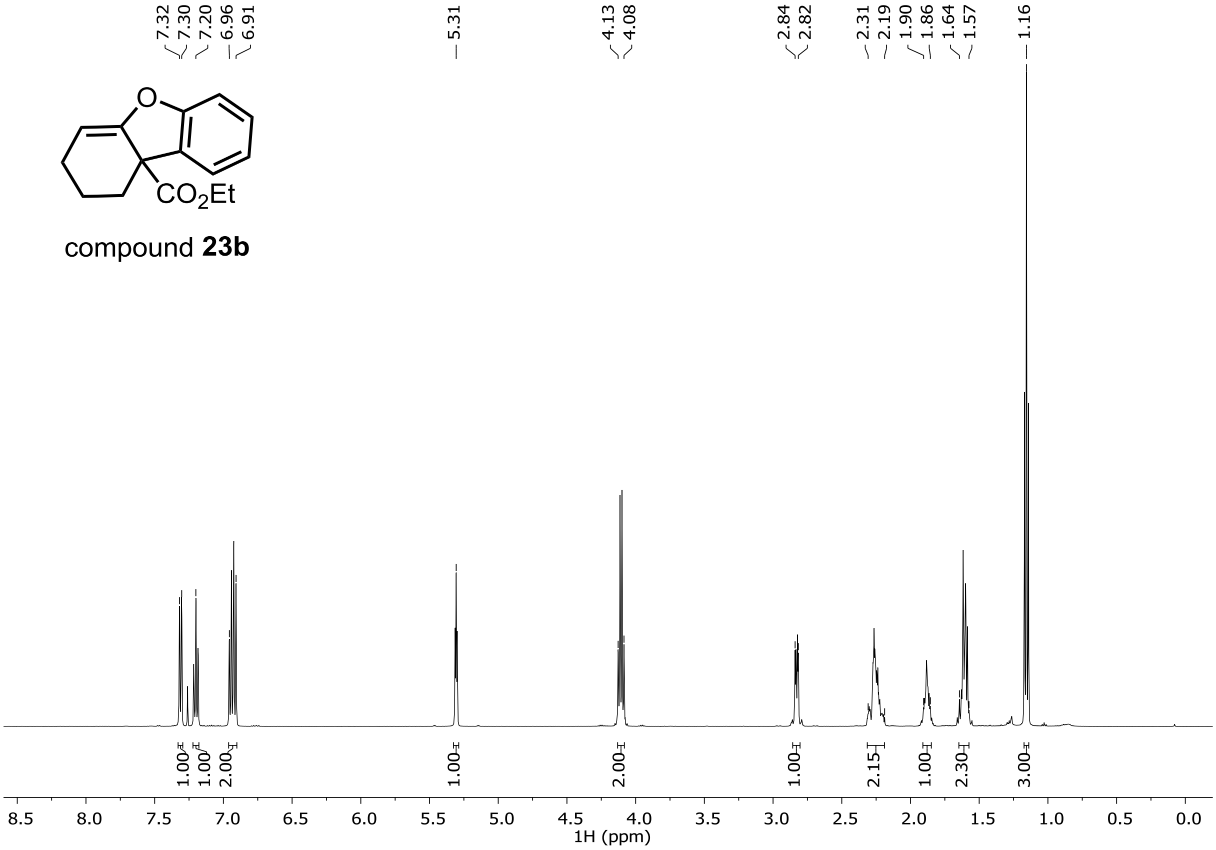
7.32
7.30
7.20
6.96
6.91
5.31
4.13
4.08
2.84
2.82
2.31
2.19
1.90
1.86
1.64
1.57
1.16
O
CO_2Et
compound **23b**
1.00
1.00
2.00
1.00
2.00
1.00
2.15
1.00
2.30
3.00
8.5 8.0 7.5 7.0 6.5 6.0 5.5 5.0 4.5 4.0 3.5 3.0 2.5 2.0 1.5 1.0 0.5 0.0
1H (ppm)

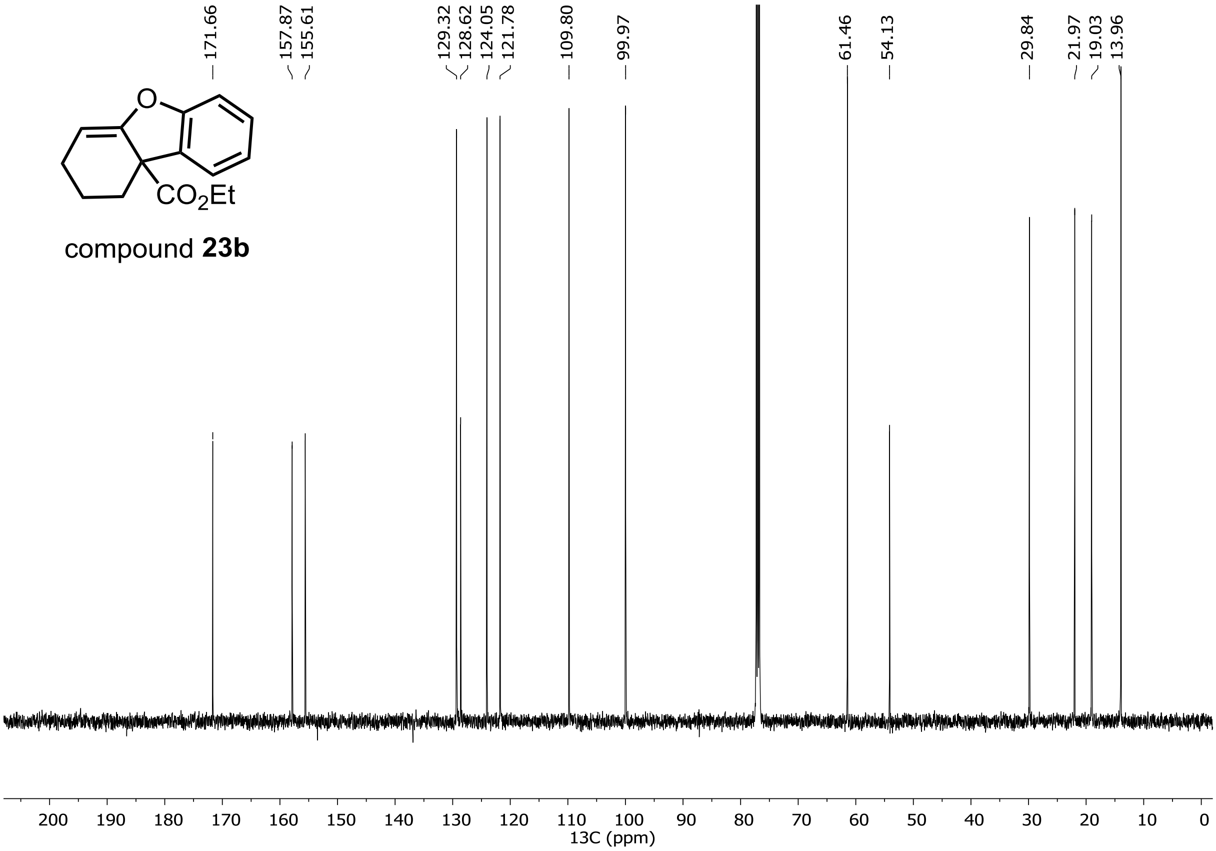
171.66
157.87
155.61
129.32
128.62
124.05
121.78
109.80
99.97
61.46
54.13
29.84
21.97
19.03
13.96
O
CO_2Et
compound **23b**
200 190 180 170 160 150 140 130 120 110 100 90 80 70 60 50 40 30 20 10 0
13C (ppm)

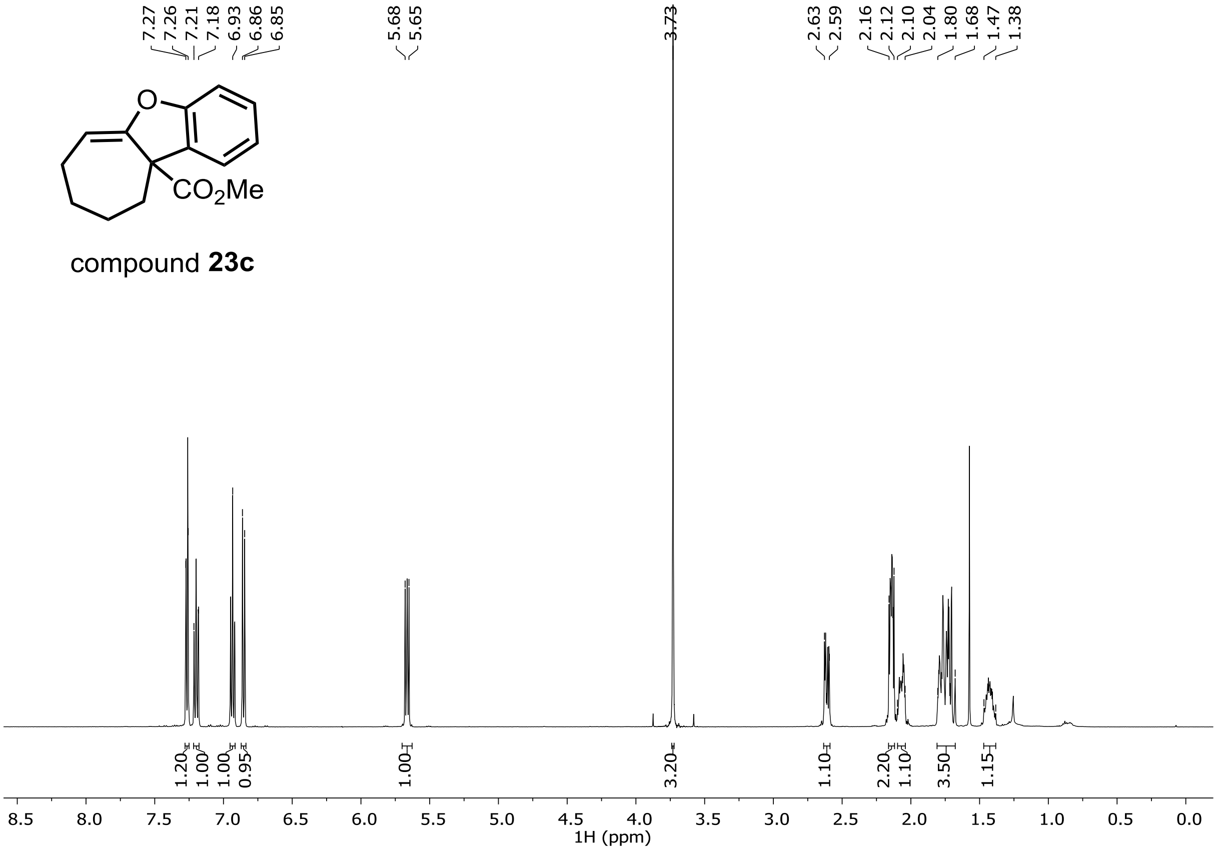
7.27
7.26
7.21
7.18
6.93
6.86
6.85
5.68
5.65
3.73
2.63
2.59
2.16
2.12
2.10
2.04
1.80
1.68
1.47
1.38
CO_2Me
compound **23c**
1.20
1.00
1.00
0.95
1.00
3.20
1.10
2.20
1.10
3.50
1.15
8.5 8.0 7.5 7.0 6.5 6.0 5.5 5.0 4.5 4.0 3.5 3.0 2.5 2.0 1.5 1.0 0.5 0.0
1H (ppm)

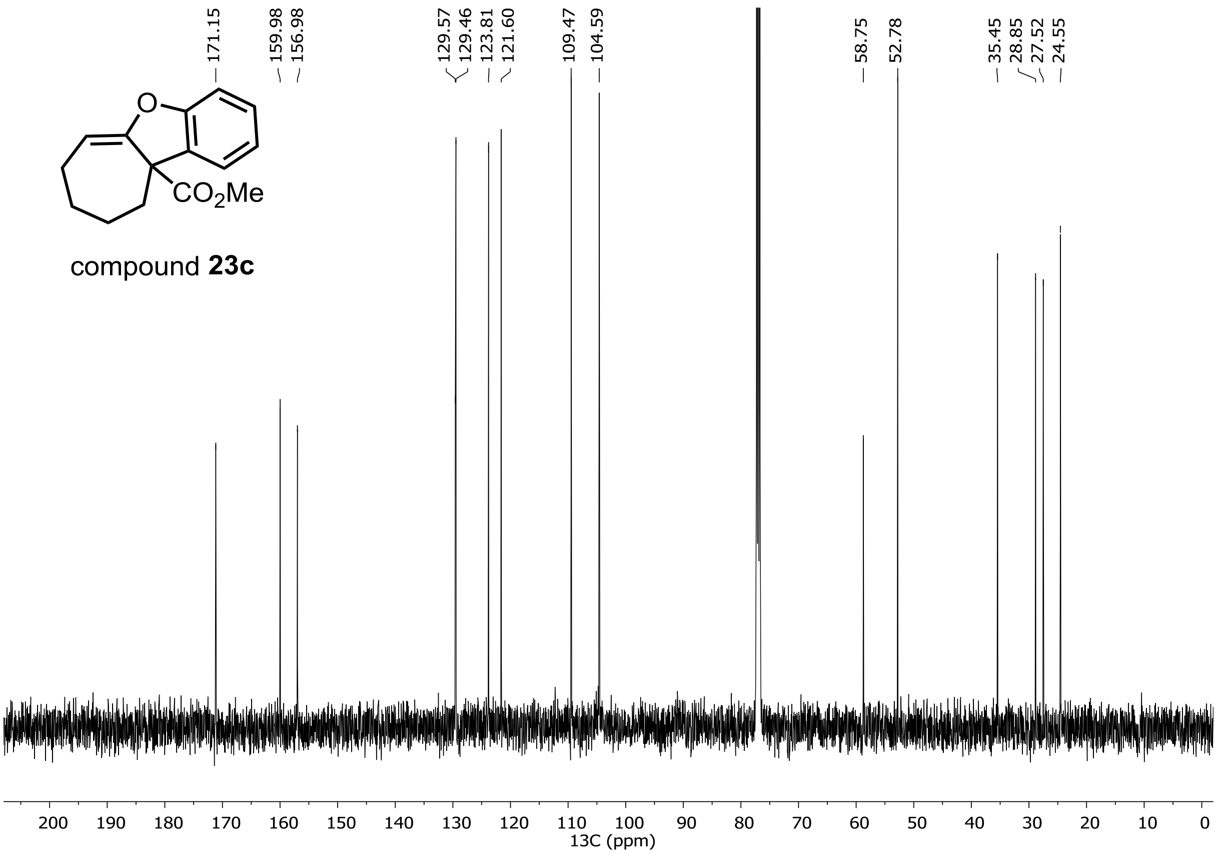
171.15
159.98
156.98
129.57
129.46
123.81
121.60
109.47
104.59
58.75
52.78
35.45
28.85
27.52
24.55
CO_2Me
compound **23c**
200 190 180 170 160 150 140 130 120 110 100 90 80 70 60 50 40 30 20 10 0
13C (ppm)

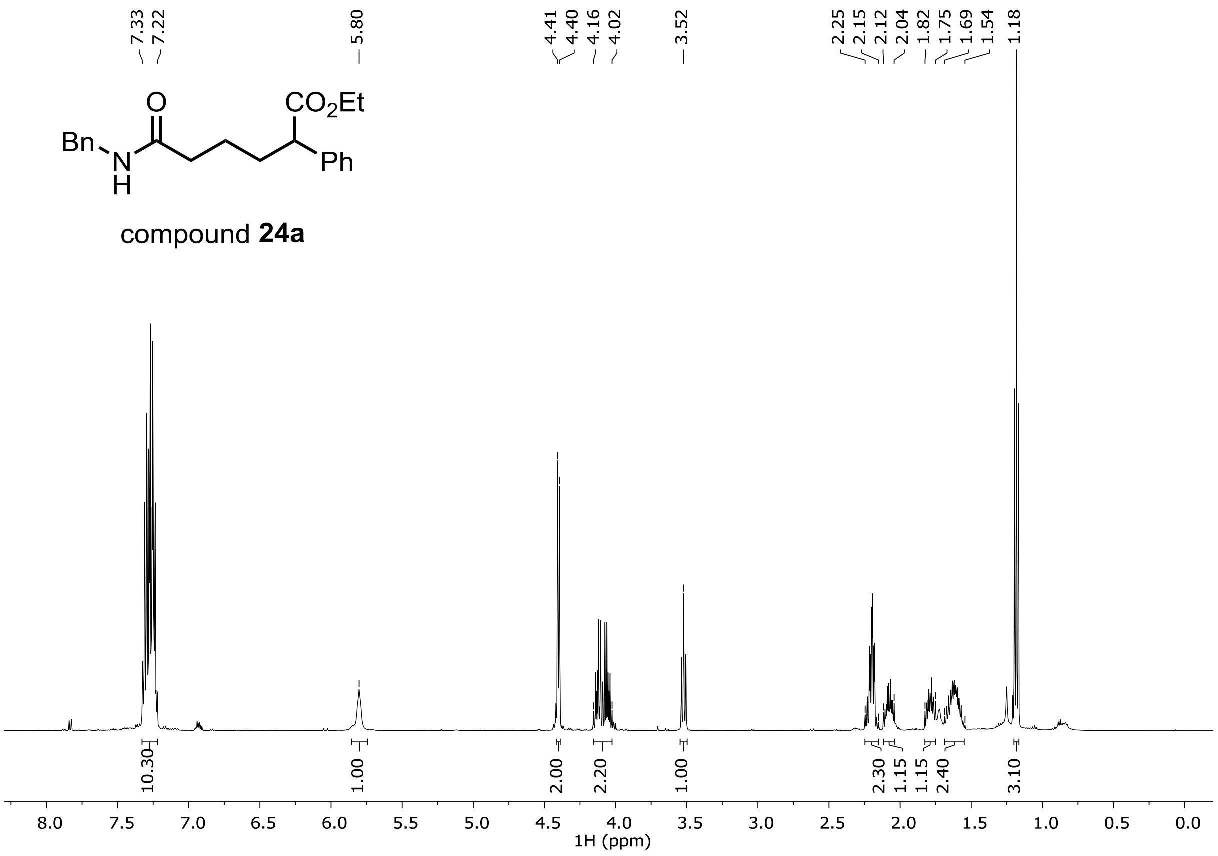
compound 24a
7.33
7.22
5.80
4.41
4.40
4.16
4.02
3.52
2.25
2.15
2.12
2.04
1.82
1.75
1.69
1.54
1.18
10.30
1.00
2.00
2.20
1.00
2.30
1.15
1.15
2.40
3.10
1H (ppm)

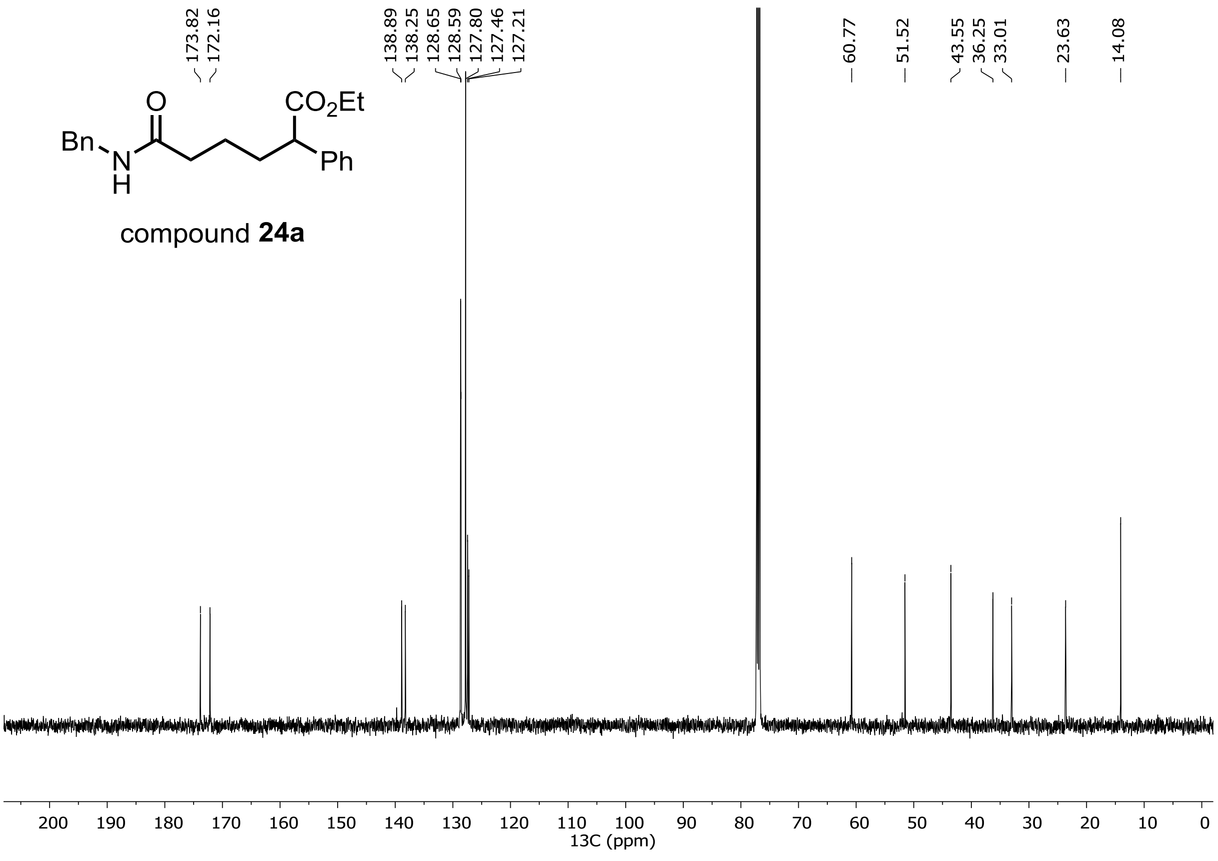
compound 24a
173.82
172.16
138.89
138.25
128.65
128.59
127.80
127.46
127.21
60.77
51.52
43.55
36.25
33.01
23.63
14.08
13C (ppm)

compound **24c**

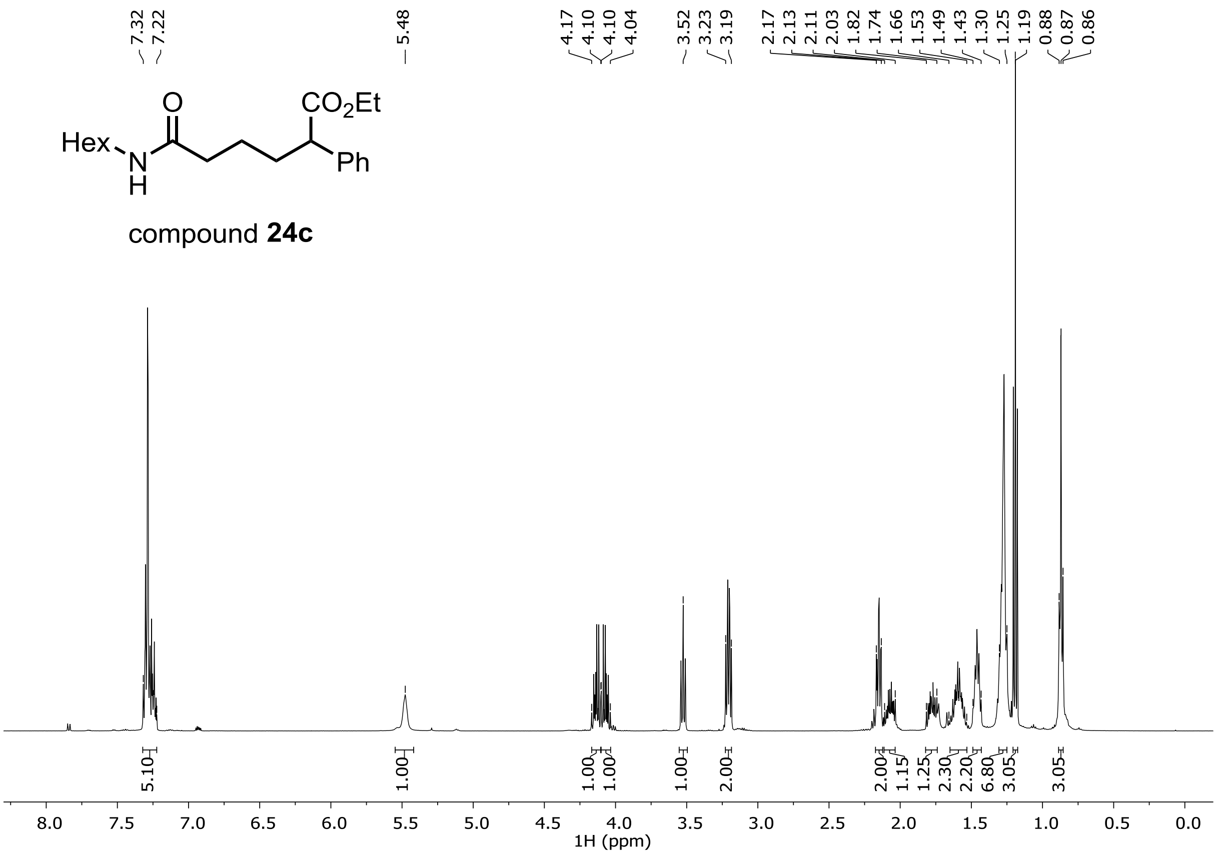

compound **24c**

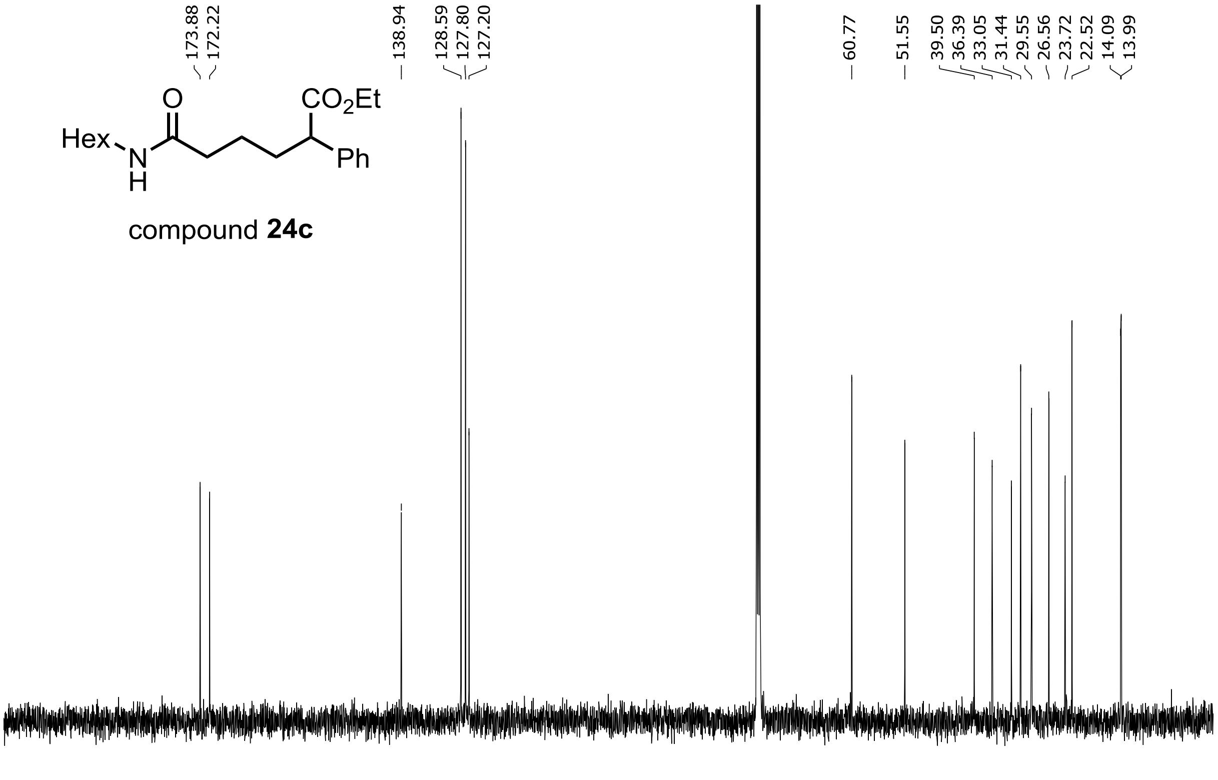

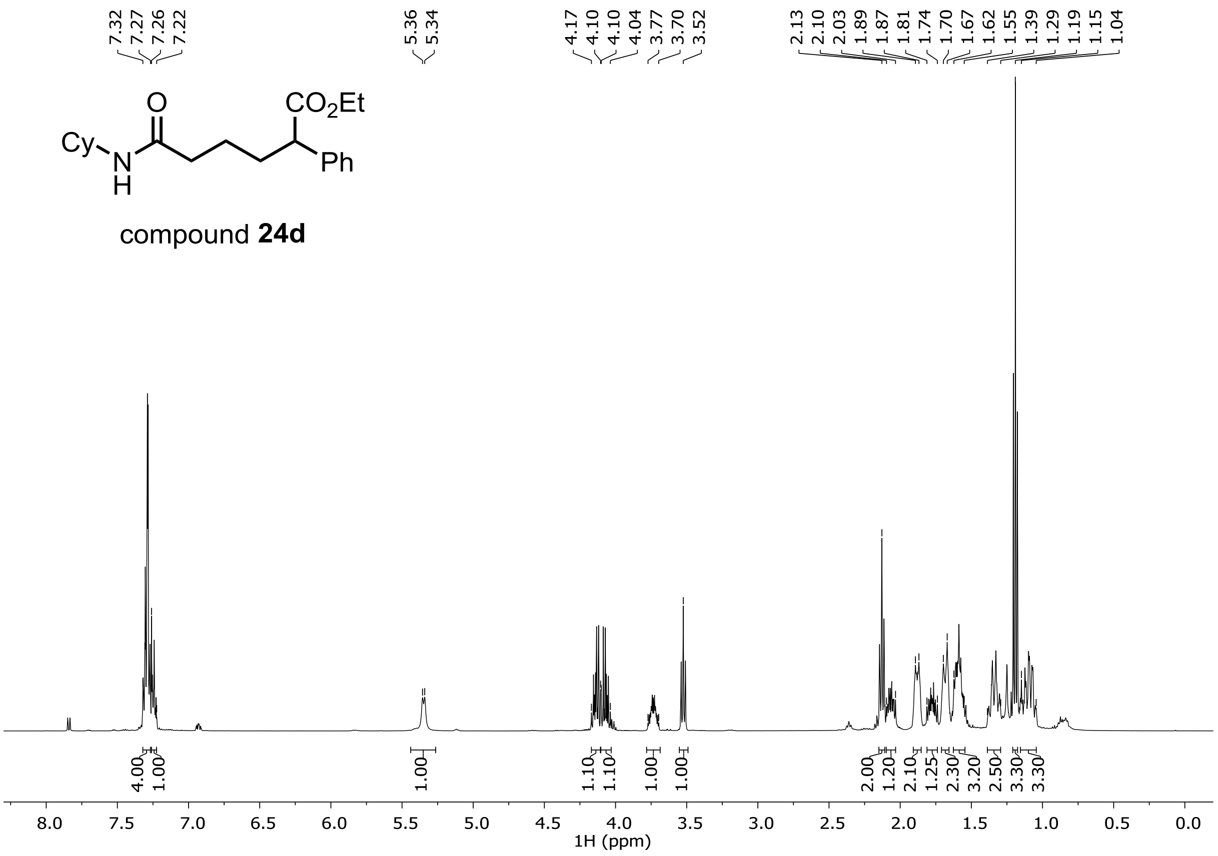

compound **24d**

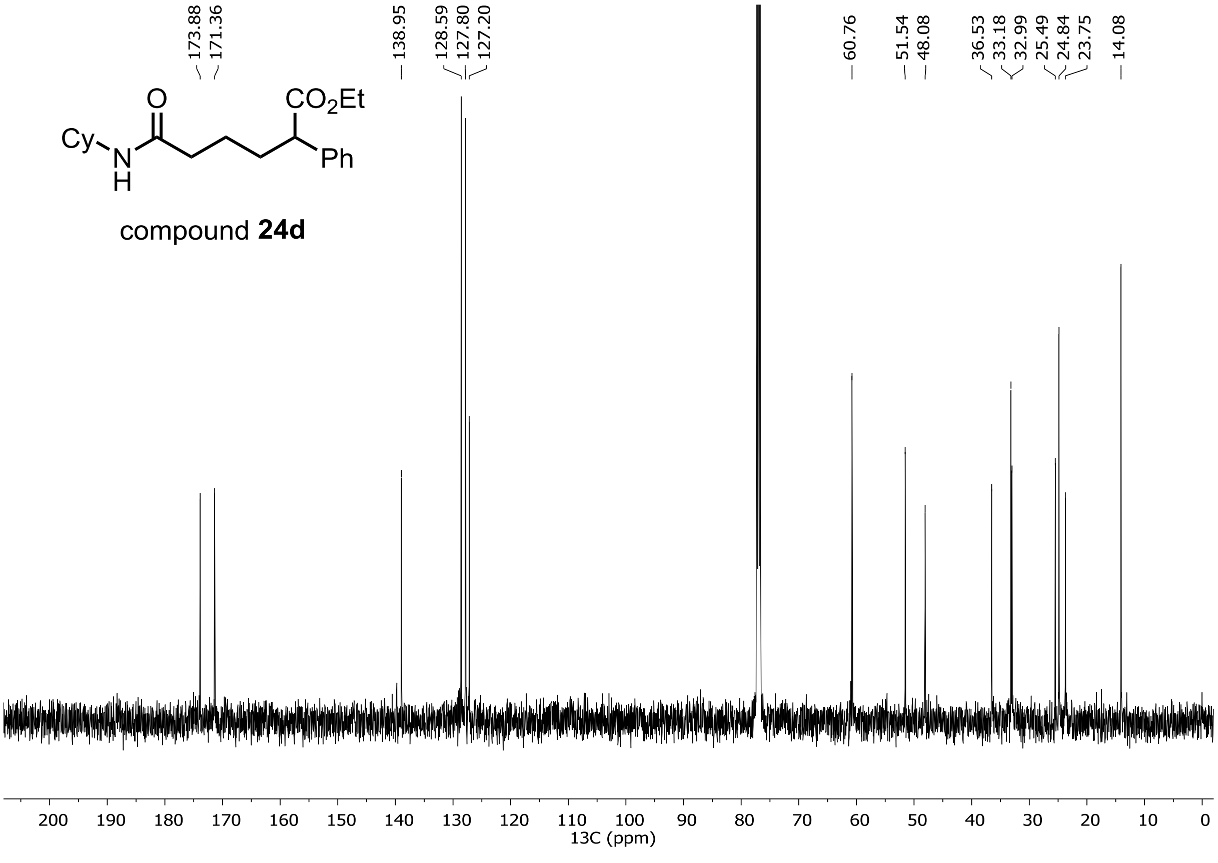

compound **24d**

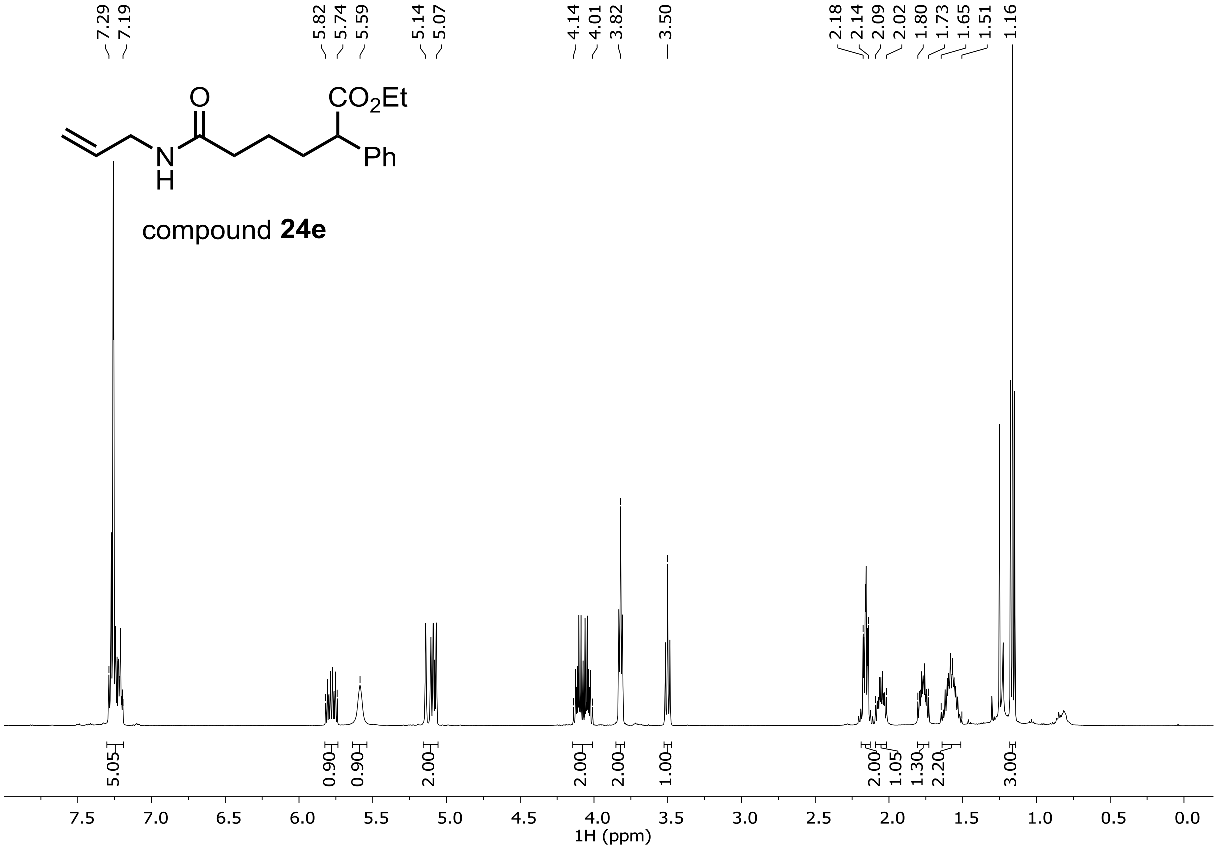
7.29
7.19
5.82
5.74
5.59
5.14
5.07
4.14
4.01
3.82
3.50
2.18
2.14
2.09
2.02
1.80
1.73
1.65
1.51
1.16
CO2Et
O
N
H
Ph
compound 24e
5.05
0.90
0.90
2.00
2.00
2.00
1.00
2.00
1.05
1.30
2.20
3.00
1H (ppm)

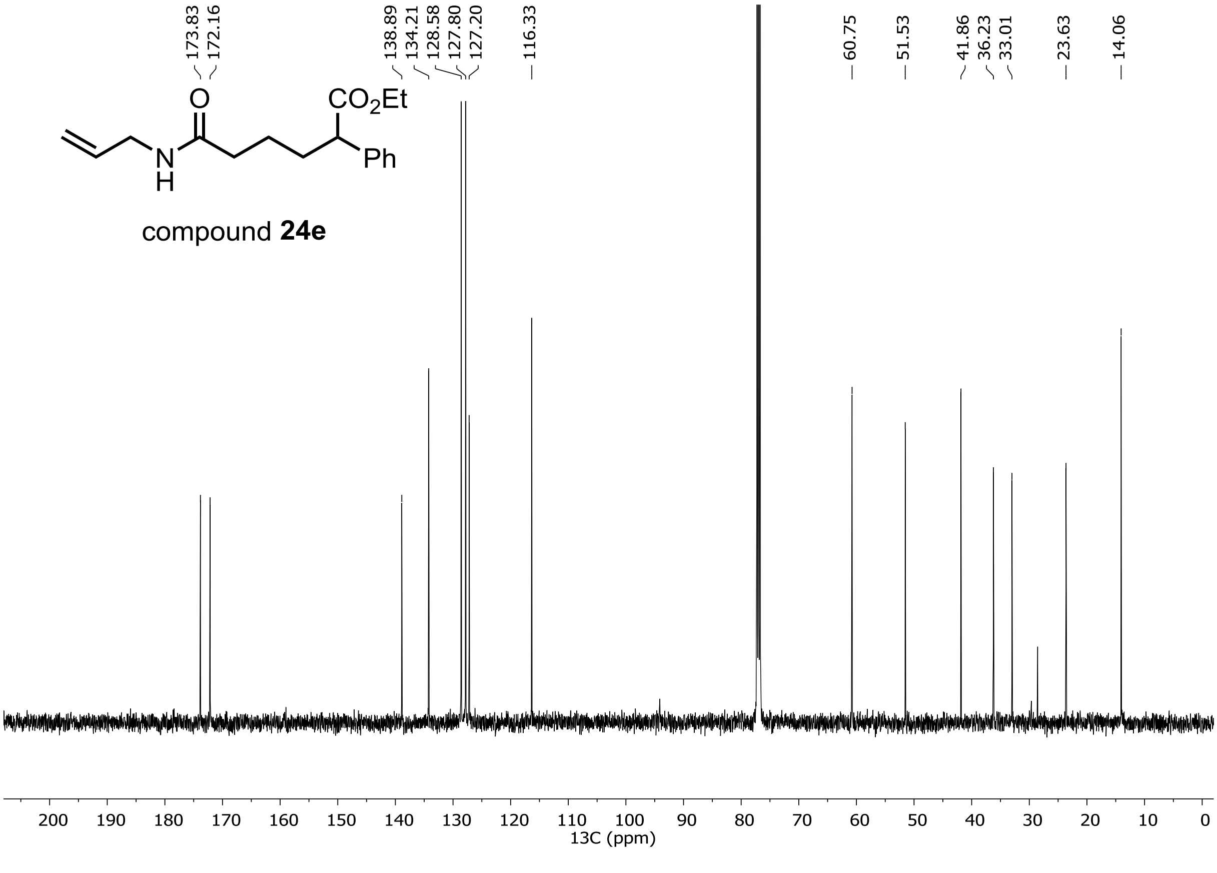
173.83
172.16
138.89
134.21
128.58
127.80
127.20
116.33
60.75
51.53
41.86
36.23
33.01
23.63
14.06
CO2Et
O
N
H
Ph
compound 24e
13C (ppm)

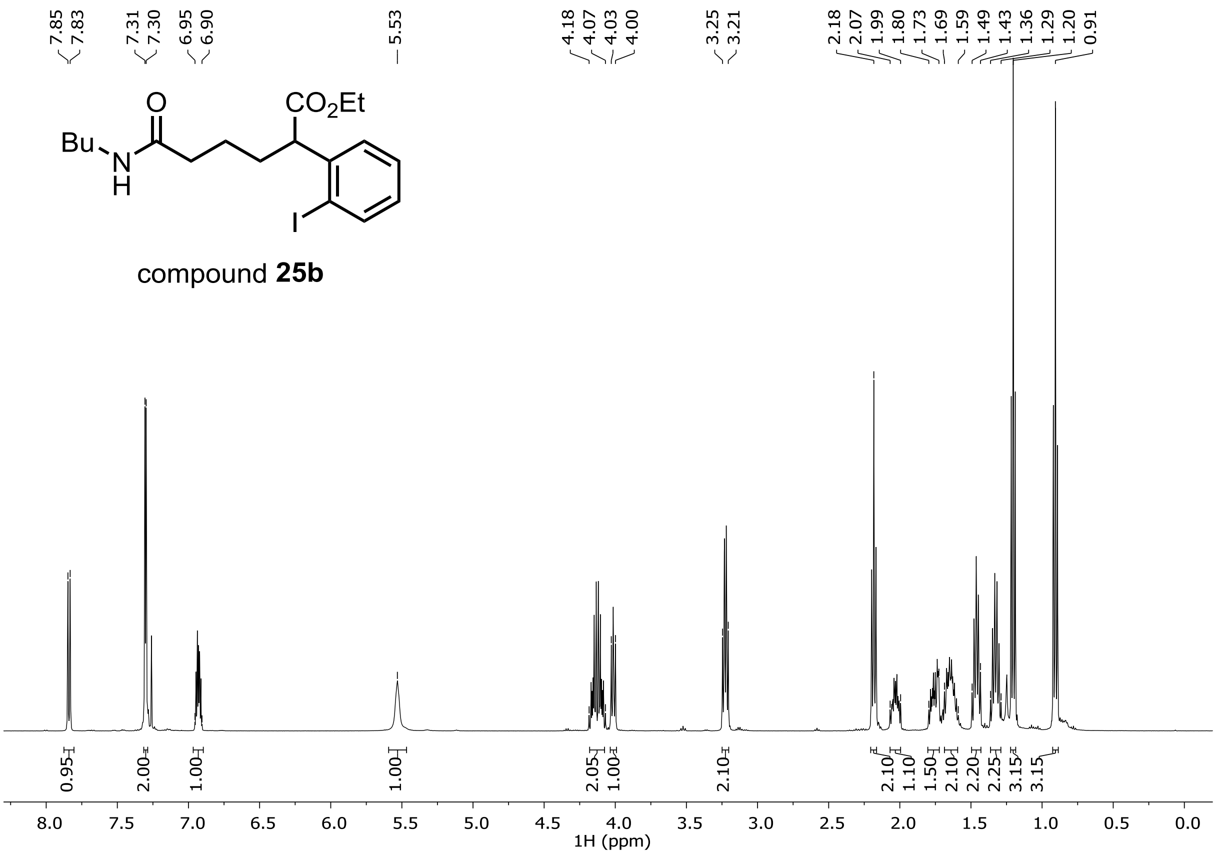
7.85 7.83 7.31 7.30 6.95 6.90 5.53 4.18 4.07 4.03 4.00 3.25 3.21 2.18 2.07 1.99 1.80 1.73 1.69 1.59 1.49 1.43 1.36 1.29 1.20 0.91
CO2Et
Bu
N
H
O
I
compound 25b
0.95 2.00 1.00 1.00 2.05 1.00 2.10 2.10 1.10 1.50 2.10 2.20 2.25 3.15 3.15
8.0 7.5 7.0 6.5 6.0 5.5 5.0 4.5 4.0 3.5 3.0 2.5 2.0 1.5 1.0 0.5 0.0
1H (ppm)

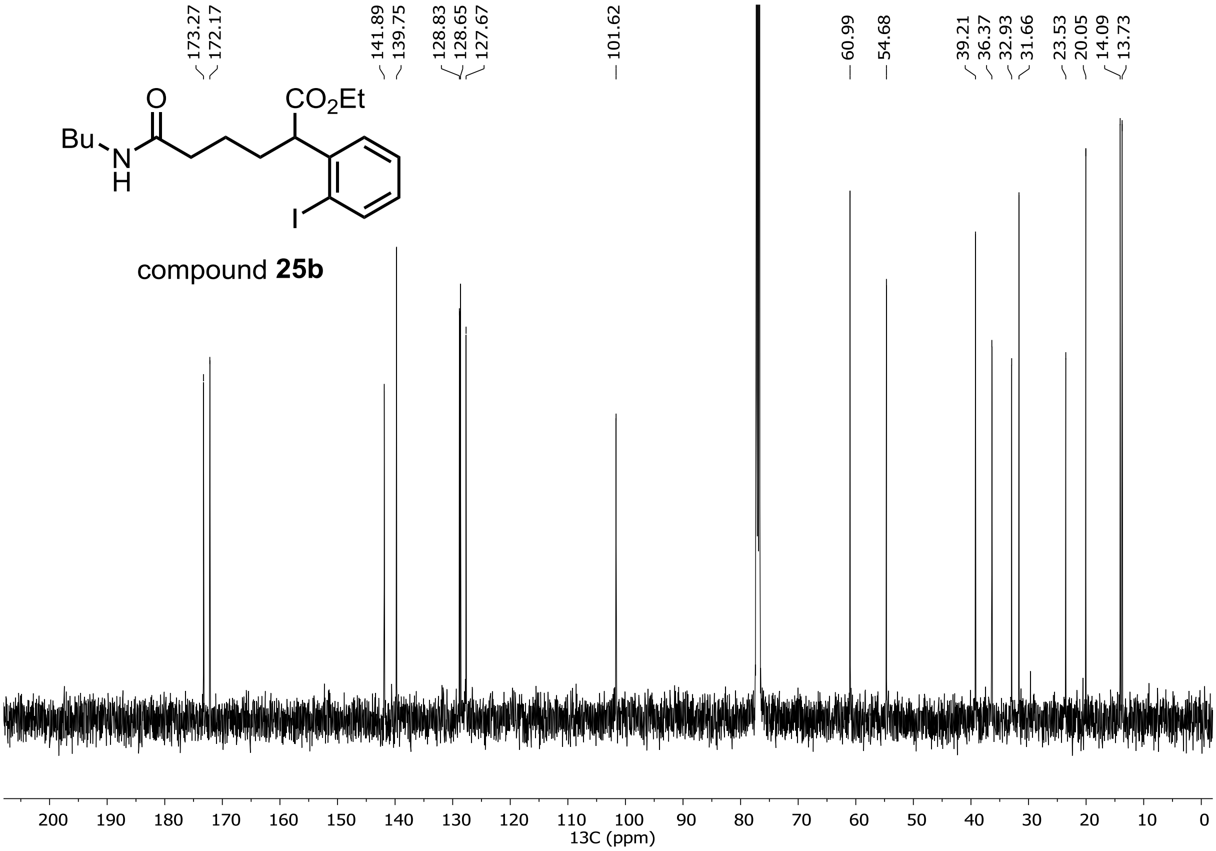
173.27 172.17 141.89 139.75 128.83 128.65 127.67 101.62 60.99 54.68 39.21 36.37 32.93 31.66 23.53 20.05 14.09 13.73
CO2Et
Bu
N
H
O
I
compound 25b
200 190 180 170 160 150 140 130 120 110 100 90 80 70 60 50 40 30 20 10 0
13C (ppm)

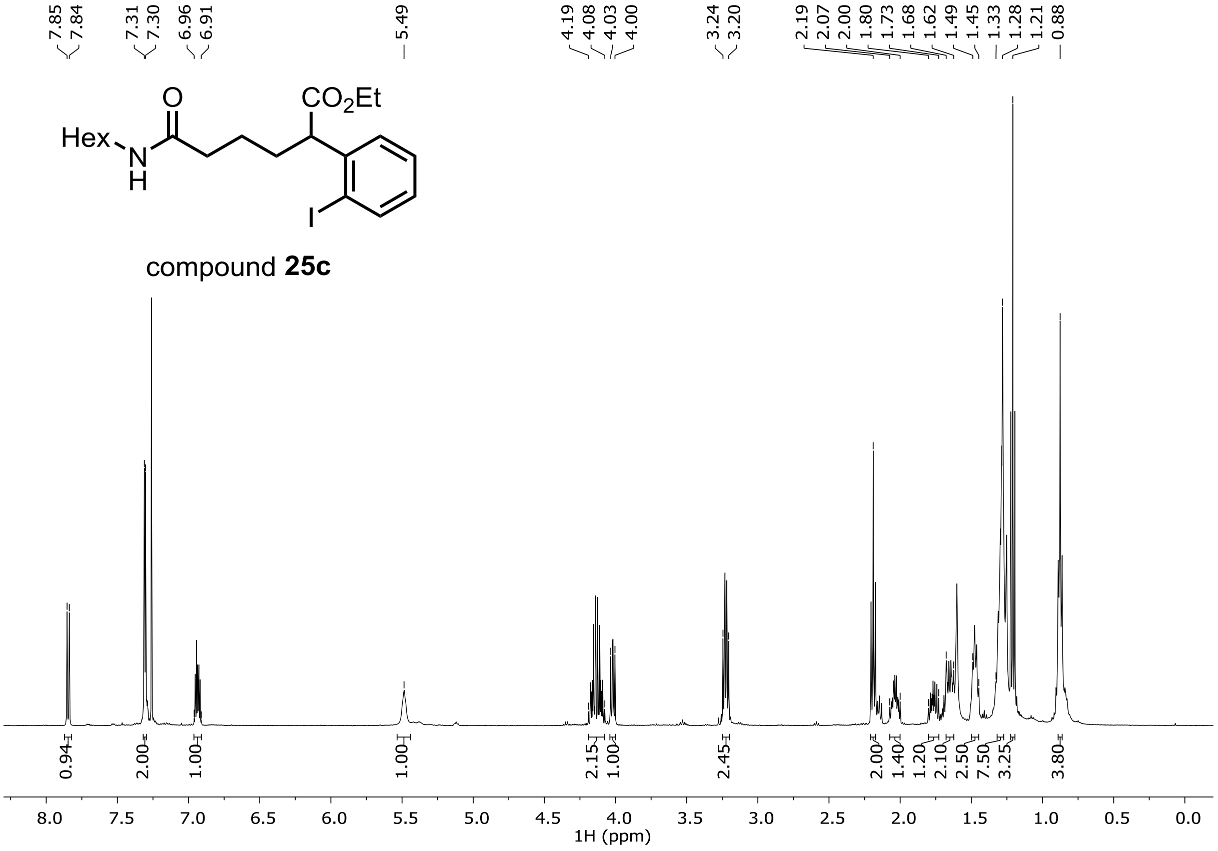
7.85
7.84
7.31
7.30
6.96
6.91
5.49
4.19
4.08
4.03
4.00
3.24
3.20
2.19
2.07
2.00
1.80
1.73
1.68
1.62
1.49
1.45
1.33
1.28
1.21
0.88
O
CO2Et
Hex
N
H
I
compound 25c
0.94
2.00
1.00
1.00
2.15
1.00
2.45
2.00
1.40
1.20
2.10
2.50
7.50
3.25
3.80
8.0 7.5 7.0 6.5 6.0 5.5 5.0 4.5 4.0 3.5 3.0 2.5 2.0 1.5 1.0 0.5 0.0
1H (ppm)

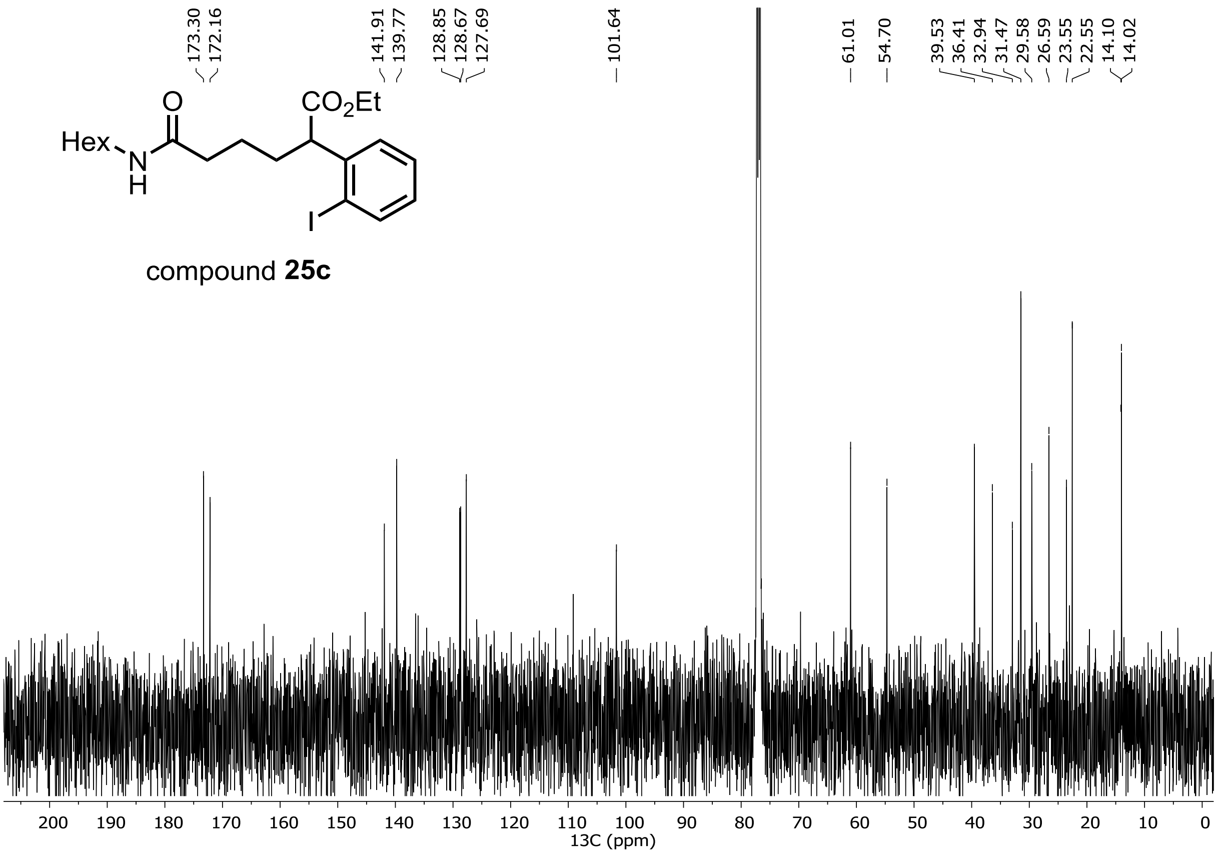
173.30
172.16
141.91
139.77
128.85
128.67
127.69
101.64
61.01
54.70
39.53
36.41
32.94
31.47
29.58
26.59
23.55
22.55
14.10
14.02
O
CO2Et
Hex
N
H
I
compound 25c
200 190 180 170 160 150 140 130 120 110 100 90 80 70 60 50 40 30 20 10 0
13C (ppm)

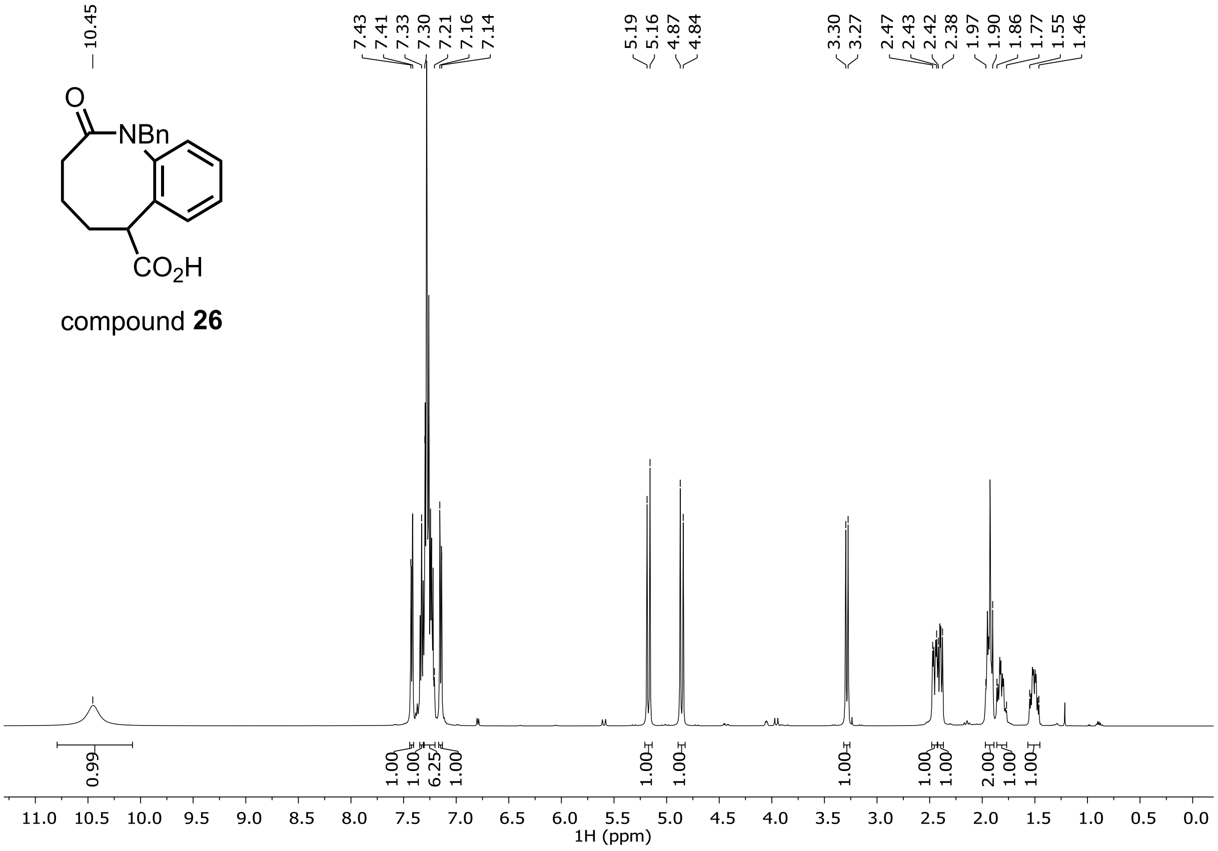

compound **26**

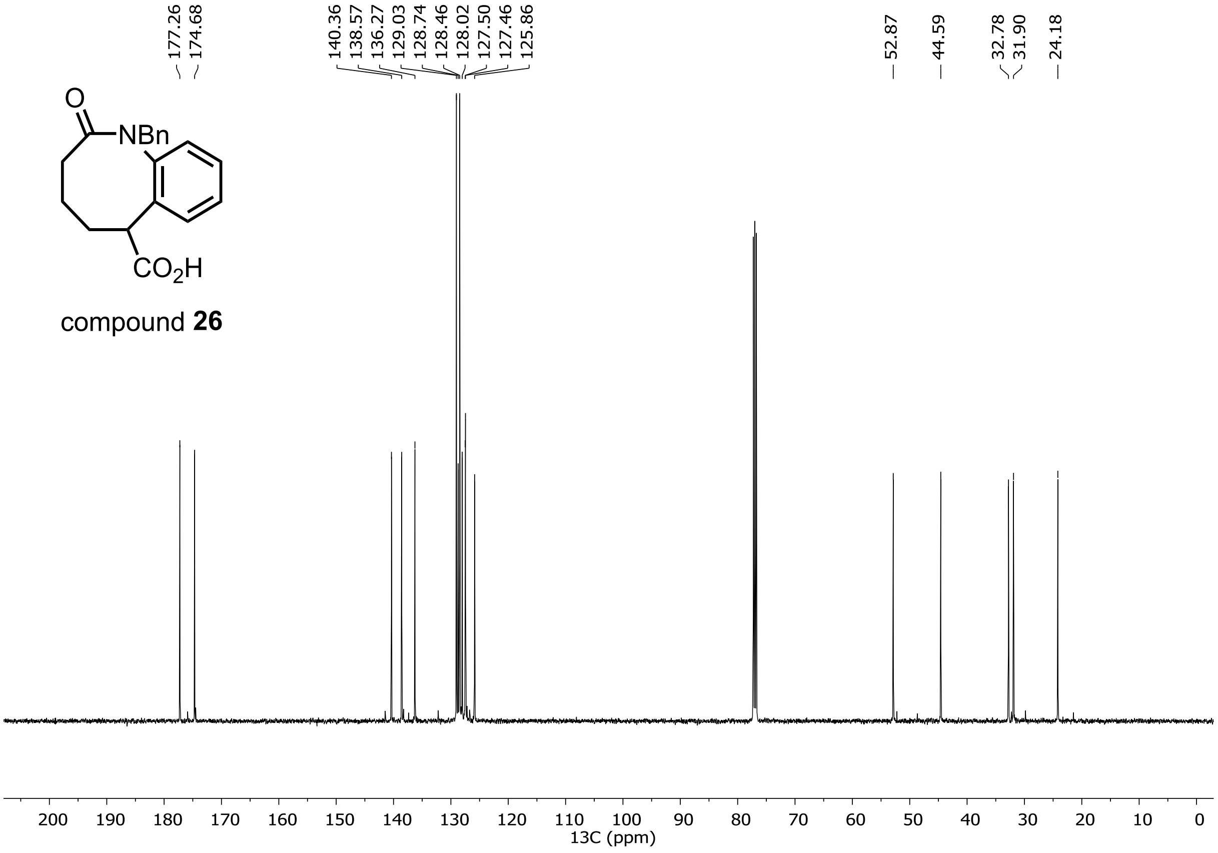

compound **26**

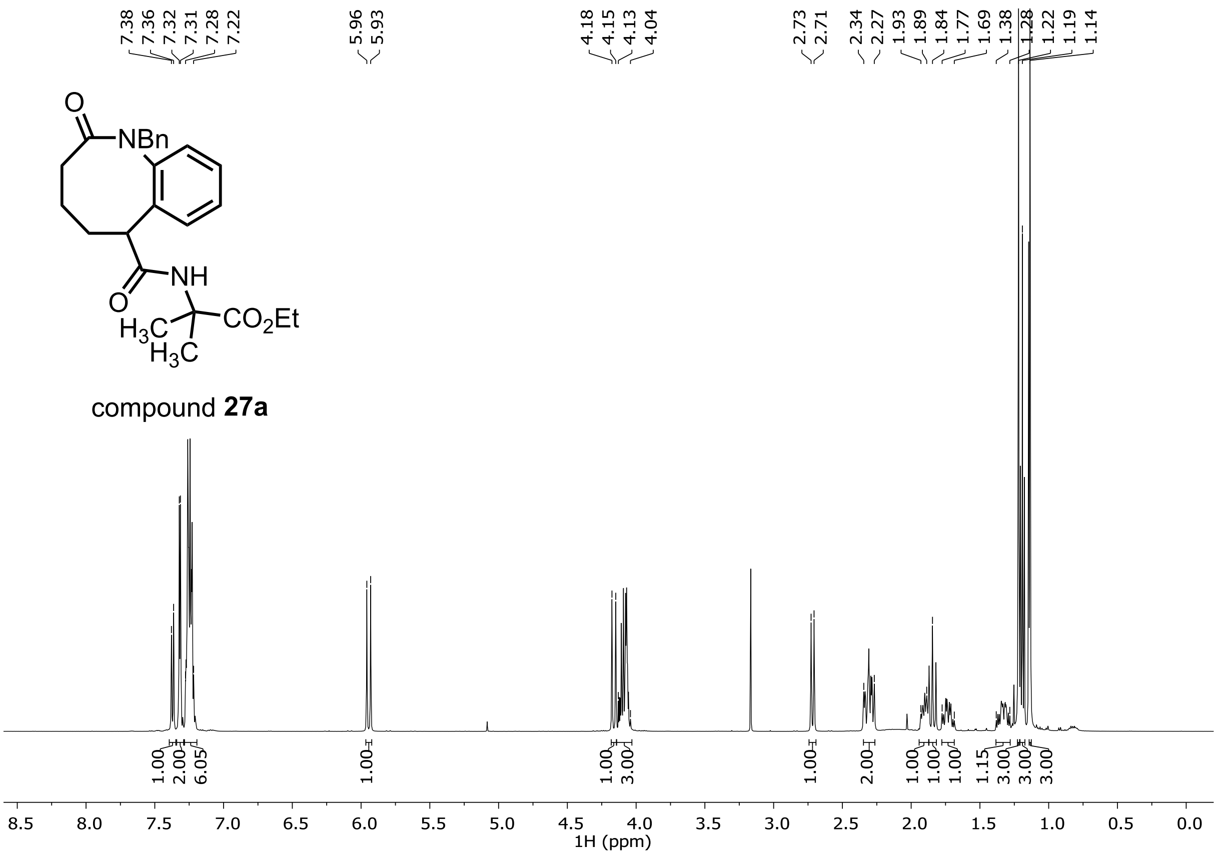

7.38 7.36 7.32 7.31 7.28 7.22
5.96 5.93
4.18 4.15 4.13 4.04
2.73 2.71
2.34 2.27 1.93 1.89 1.84 1.77 1.69 1.38 1.28 1.22 1.19 1.14
O
NBn
NH
O
H_3C
CO_2Et
H_3C
compound **27a**
1.00 2.00 6.05
1.00
1.00 3.00
1.00
2.00
1.00 1.00 1.00 1.15 3.00 3.00 3.00
8.5 8.0 7.5 7.0 6.5 6.0 5.5 5.0 4.5 4.0 3.5 3.0 2.5 2.0 1.5 1.0 0.5 0.0
1H (ppm)

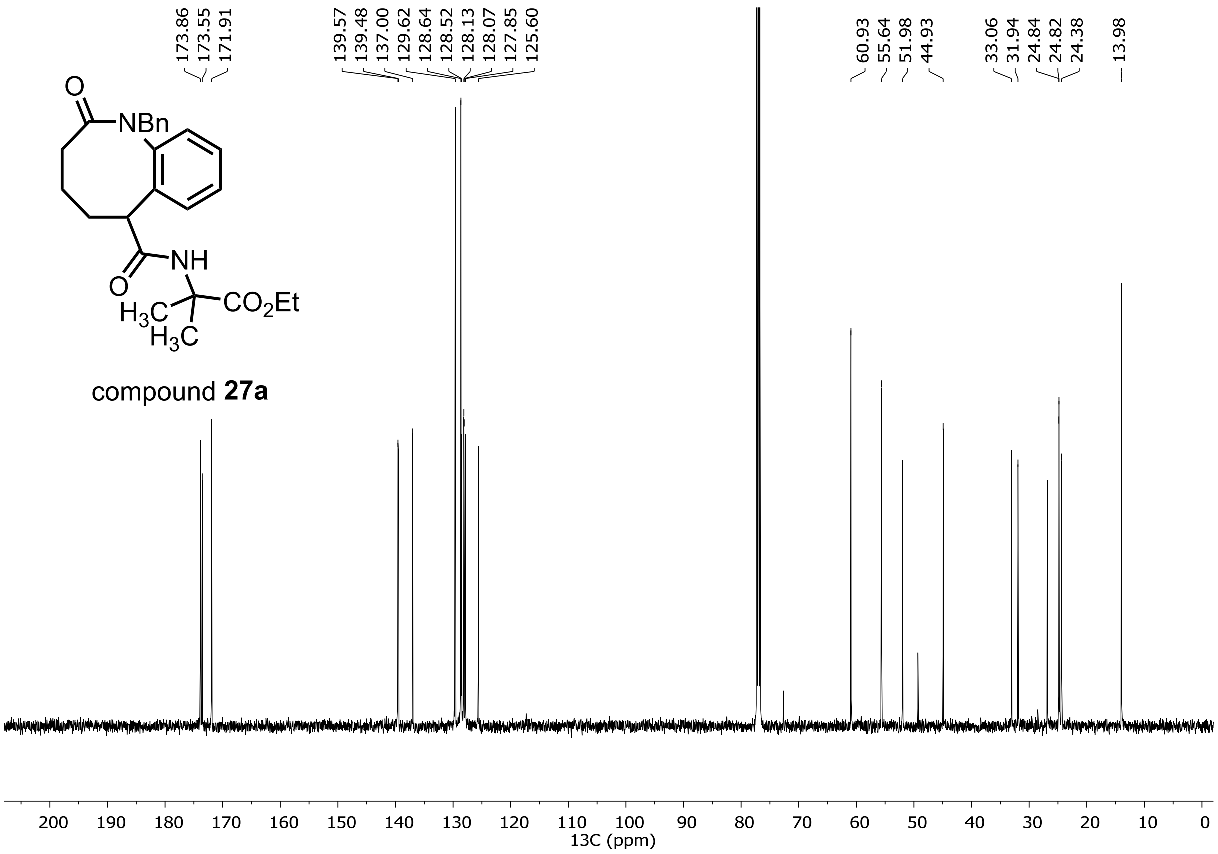

173.86 173.55 171.91
139.57 139.48 137.00 129.62 128.64 128.52 128.13 128.07 127.85 125.60
60.93 55.64 51.98 44.93
33.06 31.94 24.84 24.82 24.38
13.98
O
NBn
NH
O
H_3C
CO_2Et
H_3C
compound **27a**
200 190 180 170 160 150 140 130 120 110 100 90 80 70 60 50 40 30 20 10 0
13C (ppm)

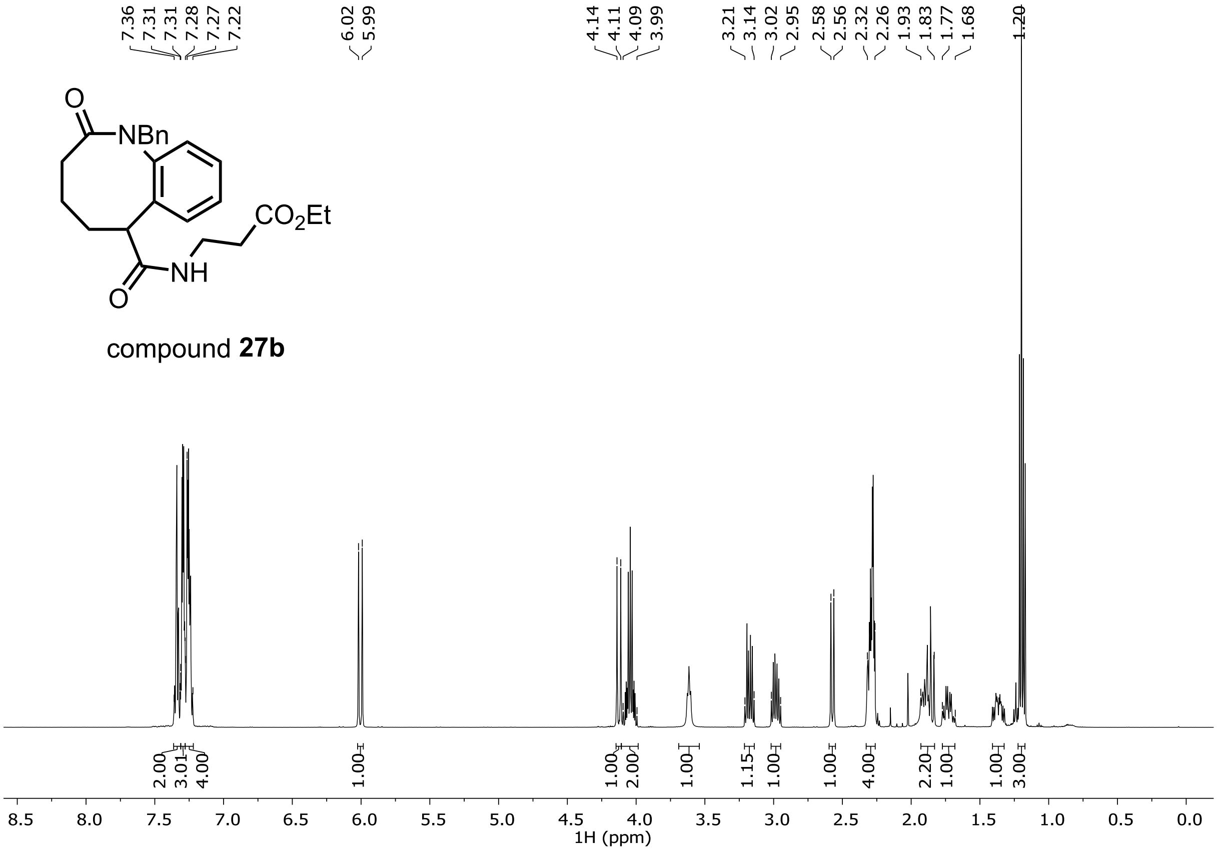

compound **27b**

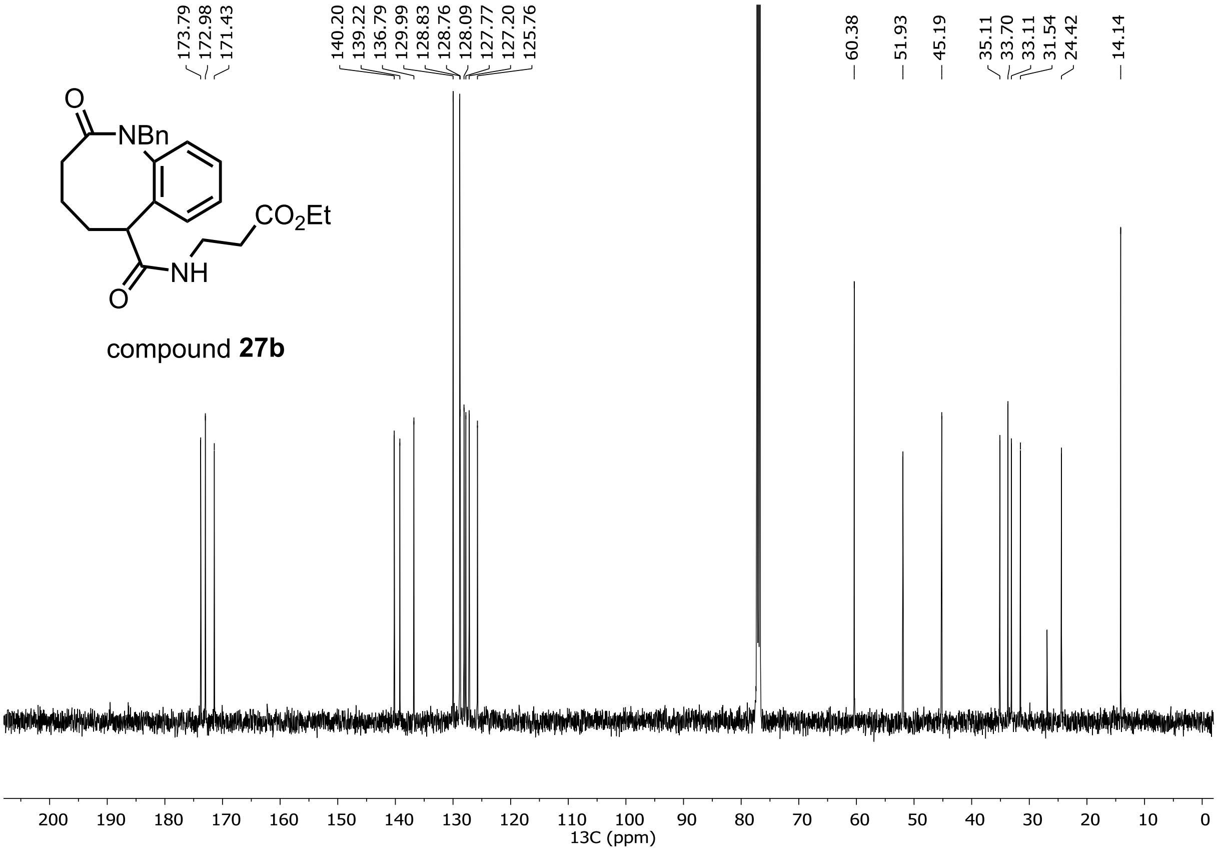

compound **27b**

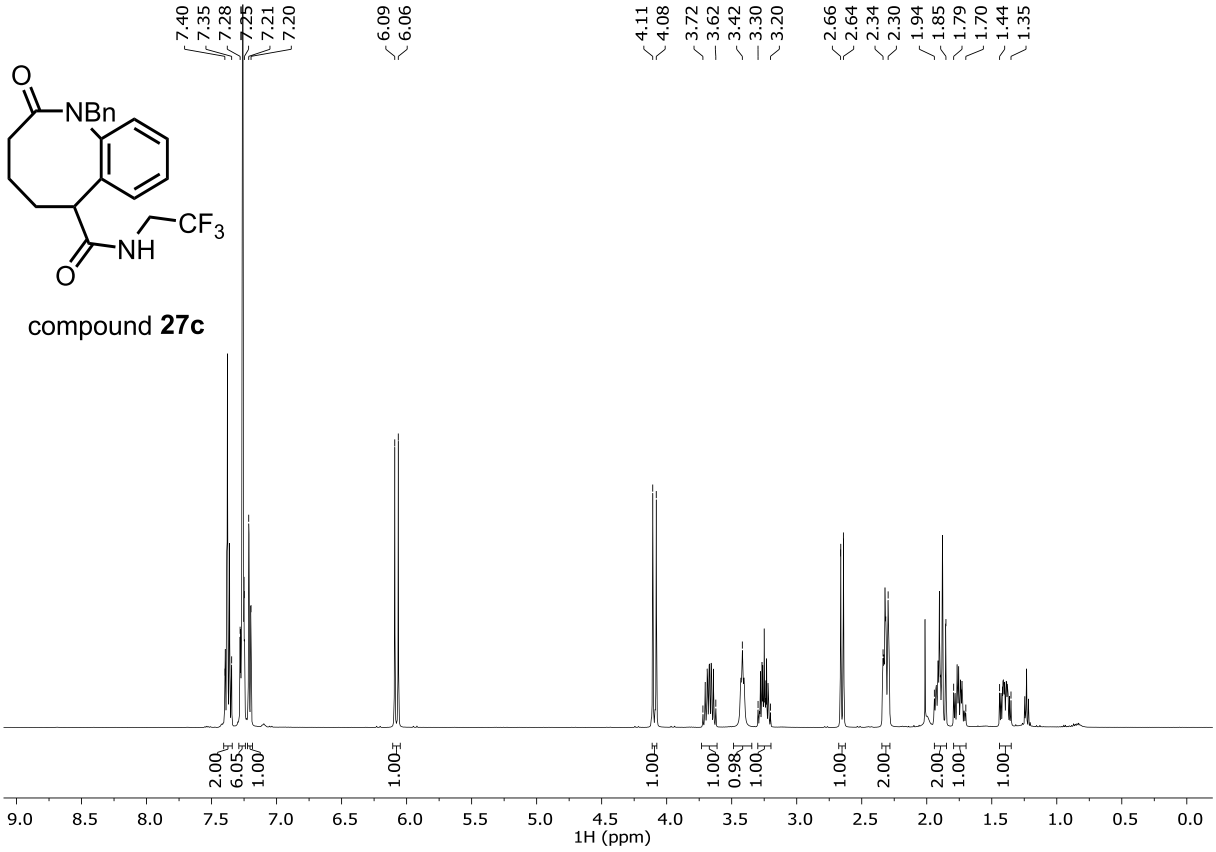

7.40 7.35 7.28 7.25 7.21 7.20 6.09 6.06 4.11 4.08 3.72 3.62 3.42 3.30 3.20 2.66 2.64 2.34 2.30 1.94 1.85 1.79 1.70 1.44 1.35
NBn
CF3
NH
compound 27c
2.00 6.05 1.00 1.00 1.00 1.00 0.98 1.00 1.00 2.00 2.00 1.00 1.00
1H (ppm)

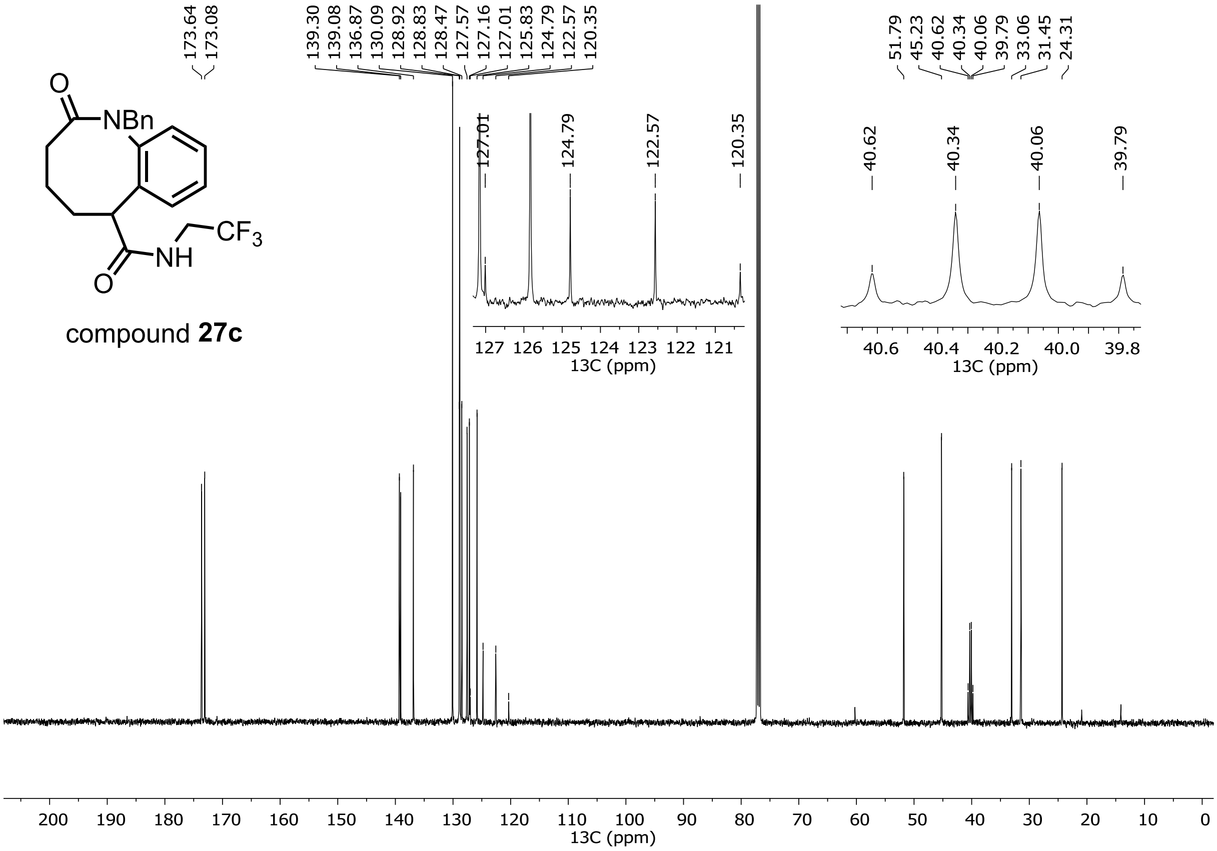

173.64 173.08 139.30 139.08 136.87 130.09 128.92 128.83 128.47 127.57 127.16 127.01 125.83 124.79 122.57 120.35 51.79 45.23 40.62 40.34 40.06 39.79 33.06 31.45 24.31
NBn
CF3
NH
compound 27c
13C (ppm)

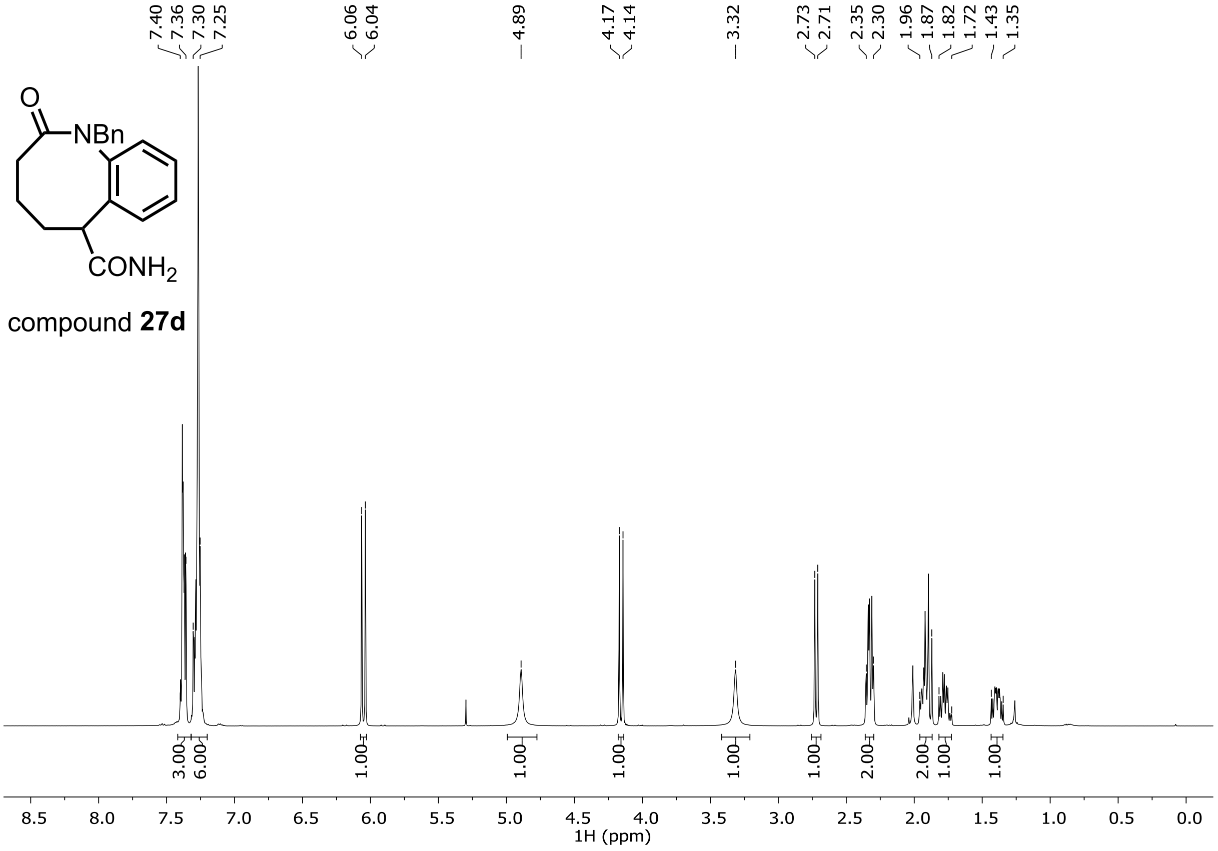
7.40
7.36
7.30
7.25
6.06
6.04
4.89
4.17
4.14
3.32
2.73
2.71
2.35
2.30
1.96
1.87
1.82
1.72
1.43
1.35
O
NBn
CONH2
compound 27d
3.00
6.00
1.00
1.00
1.00
1.00
1.00
2.00
2.00
1.00
1.00
8.5 8.0 7.5 7.0 6.5 6.0 5.5 5.0 4.5 4.0 3.5 3.0 2.5 2.0 1.5 1.0 0.5 0.0
1H (ppm)

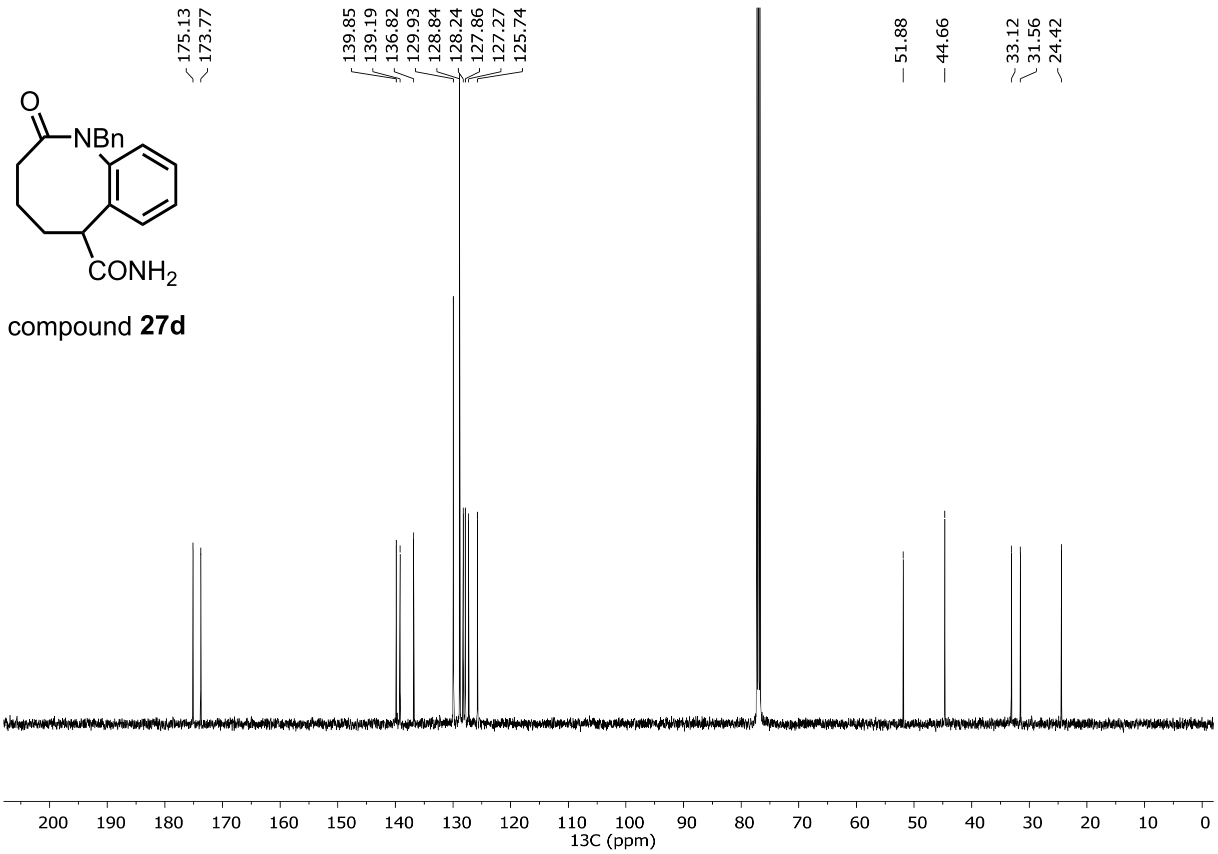
175.13
173.77
139.85
139.19
136.82
129.93
128.84
128.24
127.86
127.27
125.74
51.88
44.66
33.12
31.56
24.42
O
NBn
CONH2
compound 27d
200 190 180 170 160 150 140 130 120 110 100 90 80 70 60 50 40 30 20 10 0
13C (ppm)

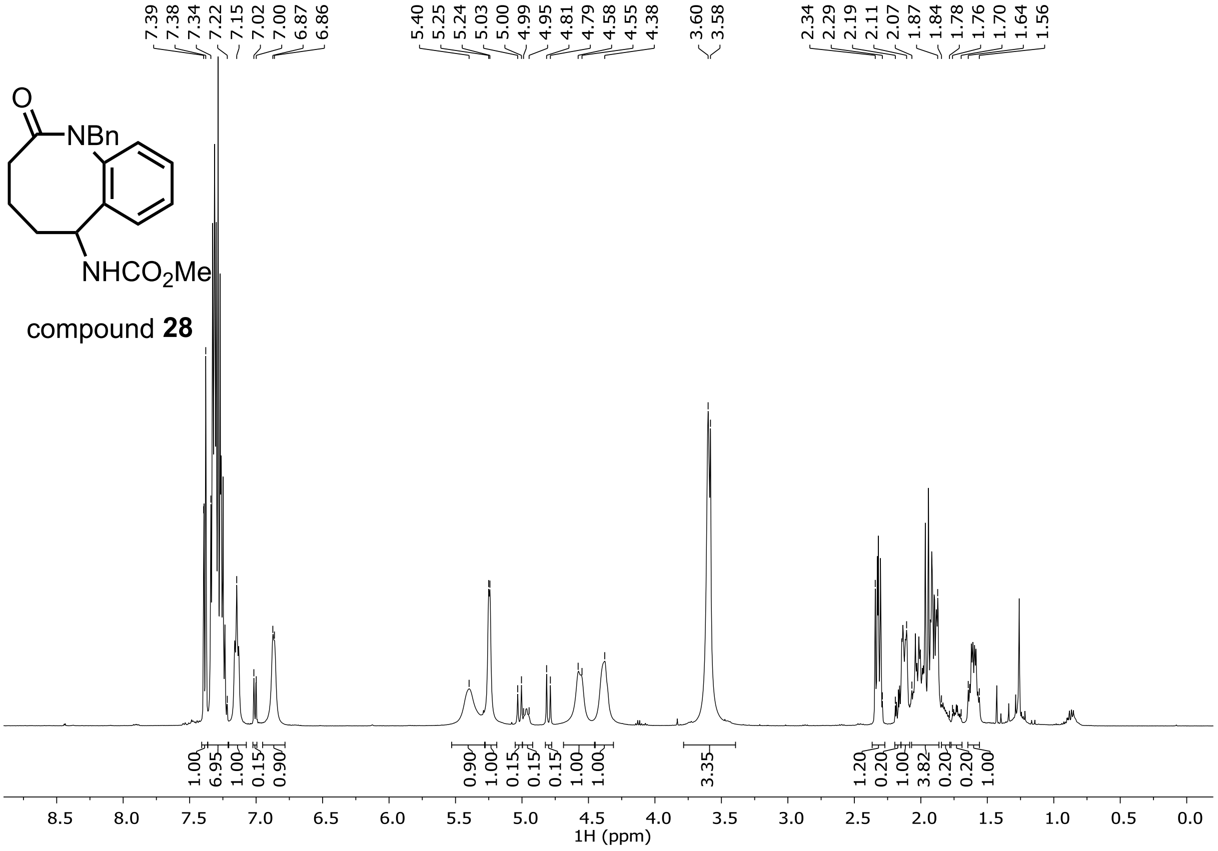
7.39 7.38 7.34 7.22 7.15 7.02 7.00 6.87 6.86
5.40 5.25 5.24 5.03 5.00 4.99 4.95 4.81 4.79 4.58 4.55 4.38
3.60 3.58
2.34 2.29 2.19 2.11 2.07 1.87 1.84 1.78 1.76 1.70 1.64 1.56
O
NBn
NHCO2Me
compound 28
1.00 6.95 1.00 0.15 0.90
0.90 1.00 0.15 0.15 0.15 1.00 1.00
3.35
1.20 0.20 1.00 3.82 0.20 0.20 1.00
8.5 8.0 7.5 7.0 6.5 6.0 5.5 5.0 4.5 4.0 3.5 3.0 2.5 2.0 1.5 1.0 0.5 0.0
1H (ppm)

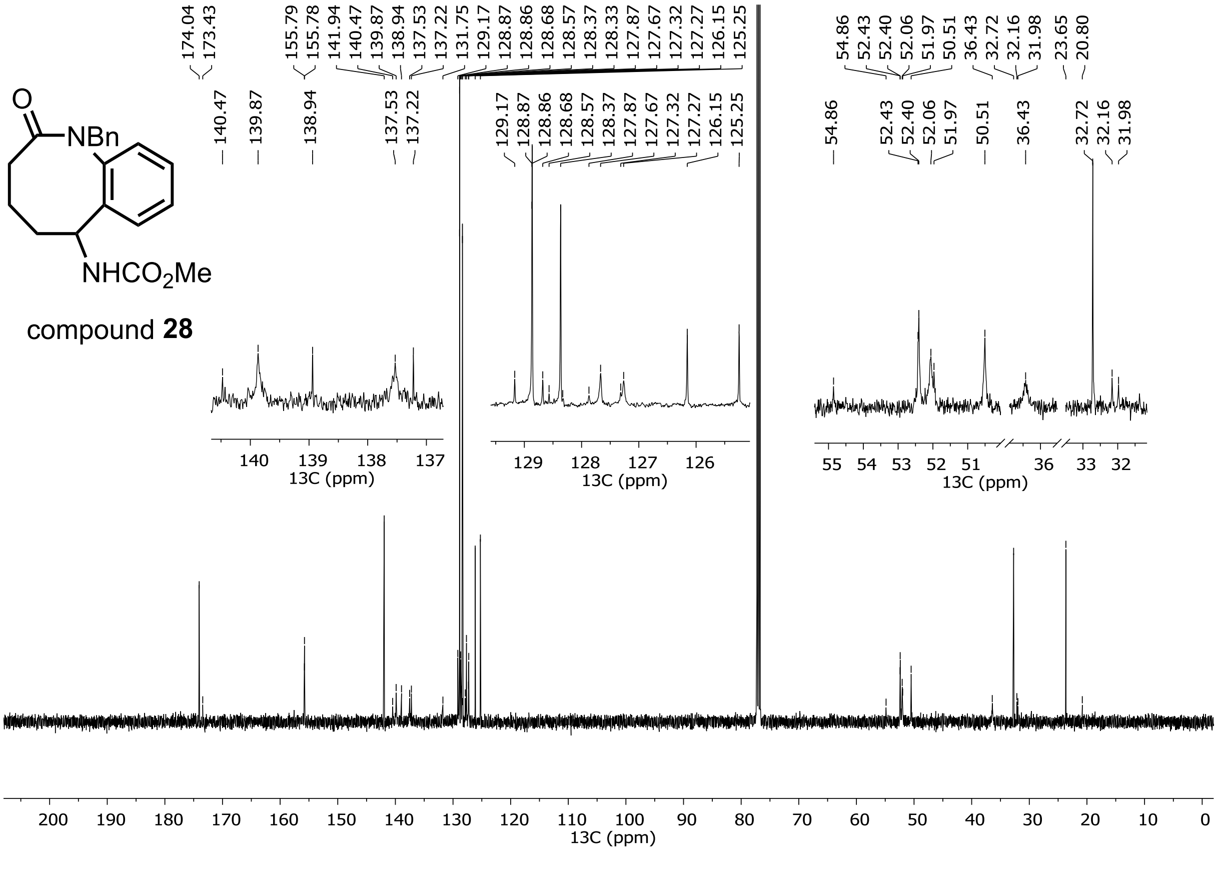
174.04 173.43
155.79 155.78 141.94 140.47 139.87 138.94 137.53 137.22 131.75 129.17 128.87 128.86 128.68 128.57 128.37 128.33 127.87 127.67 127.32 127.27 126.15 125.25
54.86 52.43 52.40 52.06 51.97 50.51 36.43 32.72 32.16 31.98 23.65 20.80
O
NBn
NHCO2Me
compound 28
140.47 139.87 138.94 137.53 137.22
140 139 138 137
13C (ppm)
129.17 128.87 128.86 128.68 128.57 128.37 127.87 127.67 127.32 127.27 126.15 125.25
129 128 127 126
13C (ppm)
54.86 52.43 52.40 52.06 51.97 50.51 36.43 32.72 32.16 31.98
55 54 53 52 51 36 33 32
13C (ppm)
200 190 180 170 160 150 140 130 120 110 100 90 80 70 60 50 40 30 20 10 0
13C (ppm)
